# Chronic Larval Exposure to Lambda-Cyhalothrin Alters Gene Expression in Both Larval and Adult Honey Bees (*Apis mellifera*)

**DOI:** 10.3390/insects16080833

**Published:** 2025-08-12

**Authors:** Bala Murali Krishna Vasamsetti, Kyongmi Chon, Juyeong Kim, Minju Choi, Bo-Seon Kim, Chang-Young Yoon, Sojeong Hwang, Kyeong-Hun Park

**Affiliations:** Toxicity and Risk Assessment Division, Department of Agro-Food Safety and Crop Protection, National Institute of Agricultural Sciences, Rural Development Administration, Iseo-myeon, Wanju-gun 55365, Jeollabuk-do, Republic of Korea; vbmk84@gmail.com (B.M.K.V.); kjy.sara@gmail.com (J.K.); chlalswn1102@gmail.com (M.C.); kbs9249@naver.com (B.-S.K.); evermoo2600@korea.kr (C.-Y.Y.); hsj102@korea.kr (S.H.); blueour@korea.kr (K.-H.P.)

**Keywords:** *Apis mellifera*, lambda-cyhalothrin, transcriptomic analysis, sublethal pesticide toxicity, Gene Ontology, KEGG pathway analysis

## Abstract

Lambda-cyhalothrin (LCY), a widely used pyrethroid insecticide, is toxic to bees—vital pollinators experiencing global declines; however, its molecular effects during early development remain poorly understood. We investigated the molecular mechanisms underlying chronic sublethal exposure to LCY in the larval and adult stages. Larvae were exposed to LCY (0.004 µg active ingredient/larva), with four groups examined: solvent-treated larvae group (SLG), solvent-treated adult group (SAG), LCY-treated larvae group (LLG), and LCY-treated adult group (LAG). We identified 1128 and 168 significantly altered genes in LLG vs. SLG and LAG vs. SAG, respectively, with 125 larval- and 25 adult-specific DEGs, indicating stage-dependent toxicity. LCY dysregulated processes such as cuticle formation, sulfur metabolism, oxidoreductase activity, and neuropeptide signaling in larvae, while adults exhibited altered redox balance, peptide receptor signaling, and monoamine transport. Neuroactive signaling disruptions were observed in both stages, with additional effects on motor function, amino acid metabolism, and glycolysis in larvae; whereas adults exhibited altered lipid biosynthesis and energy metabolism. Downregulated genes involved in chitin metabolism and antioxidant defenses in larvae suggested compromised exoskeletal integrity and increased vulnerability. Overall, our findings highlight the long-term molecular consequences of early-life exposure and emphasize the need for safer pesticide practices to protect pollinator health.

## Simple Summary

Honey bees are crucial for pollinating crops and maintaining balanced ecosystems, but their populations are declining around the world. One main cause is exposure to pesticides used in agriculture. In this study, we explored the impacts of lambda-cyhalothrin (LCY), a common insecticide used to control pests in crops. We exposed honey bee larvae to a very low dose of LCY and investigated its effects on their gene expression. The pesticide weakened their ability to defend against stress and disrupted important biological processes in the larvae. Exposure at the larval stage also showed toxic effects in adult bees, suggesting that even low levels of LCY can cause lasting harm. We found that it altered the activity of genes involved in development, energy metabolism, and brain function at both developmental stages. Our findings highlight the importance of implementing safer farming practices to protect pollinators.

## 1. Introduction

Honey bees play a critical role in maintaining the ecological balance through the pollination of various plants, making them essential for ecosystem stability [1]. Beyond their ecological significance, honey bees contribute valuable products, such as honey, royal jelly, and propolis, which have various nutritional, medicinal, and cosmetic applications. The decline in honey bee populations has emerged as a global concern, prompting significant research efforts to identify the reasons for this reduction [2,3]. Among the various factors contributing to this decline, pesticide exposure stands out as a major threat to honey bee health and survival [4,5,6].

During foraging, honey bees collect nectar and pollen that may be contaminated with pesticides and bring them back to the hive. Inside the hive, this contaminated nectar and pollen are transformed into honey and bee bread, which nurse bees feed to the larvae along with royal jelly, thereby exposing them to pesticide residues [5,7,8,9,10]. Such exposure can disrupt critical behaviors such as foraging, navigation, and learning, and can weaken the immune systems of bees, making them more susceptible to additional stressors, such as pathogens and habitat loss [4,10]. Notably, pesticide exposure has been shown to inhibit acetylcholinesterase activity, alter antioxidant enzyme levels such as those of catalase and superoxide dismutase, and disrupt the expression of genes related to neural signaling and immune regulation [10,11]. These physiological disruptions compromise the bees’ ability to cope with additional stressors, potentially contributing to colony collapse disorder and declines in honey bee populations [3,4,10]. Both larvae and adult bees are vulnerable to pesticide exposure, and studies have reported significant effects on survival, cognitive ability, and overall colony dynamics [12,13,14,15]. While neonicotinoids have been widely scrutinized and restricted owing to their off-target toxicity, pyrethroids are increasingly used as alternative pesticides, raising new concerns about their impact on honey bee health [11,16,17,18,19].

Lambda-cyhalothrin (LCY), a pyrethroid insecticide, has become widely used. Commonly applied to agricultural crops, including almonds, apples, and cherries, it protects against insect pests such as aphids, caterpillars, and beetles [20]. LCY acts on the nervous system of insects by altering voltage-gated sodium, calcium, and chloride channels [21]. Beyond its intended targets, LCY poses toxic risks to humans and non-target animals. In humans, in vitro exposure to LCY has been associated with chromosomal aberrations and DNA damage in lymphocytes, indicating genotoxic potential [22]. LCY also disrupts antioxidant enzyme activities and induces oxidative stress in erythrocytes [22,23]. In human astrocytes, it disrupts calcium homeostasis and triggers apoptosis [24]. Animal studies further revealed hepatotoxicity in rats, with toxicity increasing in a dose- and time-dependent manner but mitigated by ascorbic acid [25,26,27]. The underlying mechanism of LCY toxicity involves the disruption of ionic conduction across sodium, calcium, and chloride channels, leading to oxidative tissue damage [27,28], highlighting its potential for neurotoxicity, oxidative damage, and genomic changes across a range of non-target organisms.

Environmentally, LCY residues have been detected in pollen at concentrations ranging from 3.2 to 1750 µg/kg, and at 314.3 µg/kg in bee bread [10,29,30]. LCY has been shown to cause severe toxicological effects in non-target pollinators like honey bees, even at sublethal concentrations. Acute exposure significantly reduces foraging behavior, impairs learning and memory, and affects homing ability, indicating neurotoxic impacts [31,32]. Furthermore, LCY exposure during the larval stage increases mortality, causes deformities, and disrupts key developmental enzymes [11]. Laboratory assessments confirmed that both contact and oral exposure led to high mortality rates, with LD_50_ values highlighting substantial toxicity to *Apis cerana indica* [14]. Field assessments provided additional insights; semi-field and field studies found that LCY exposure (contact) reduces colony foraging activity, adversely affects brood development, and depletes stored nectar and pollen reserves [15]. Additionally, research has shown that LCY alters the gut microbiota, immune responses, and detoxification pathways, compounding concerns about its physiological impacts [6,11,33]. Furthermore, LCY disrupts foraging activities, shortens lifespan, impairs memory, and damages critical bee organs, such as the brain, hypopharyngeal glands, and midgut [32,34,35].

The Pesticide Properties Database lists LCY as an insecticide with notable toxicity to honey bees, with an acute contact LD_50_ of 0.038 μg/bee and an acute oral LD_50_ of 0.91 μg/bee [36]. Our previous research established the acute and chronic LD_50_ values of LCY in honey bee larvae as 0.058 µg a.i./larva and 0.040 µg a.i./larva, respectively [11]. These lower values compared to the adult oral LD_50_ indicate that larvae are more sensitive to LCY exposure. Furthermore, sublethal larval exposure to LCY causes wing and antenna deformities in emerged adult bees [11], pointing to lasting developmental toxicity. This highlights the need for further investigation into the molecular mechanisms underlying LCY’s impact on honey bee development.

Toxicity assessments in honey bees often focus on mortality, phenotypic changes (antenna and wing deformities), and behavioral alterations [11,12,37]. Given the chemical complexity of pesticides, the phenotypic changes observed in honey bees following exposure are likely driven by intricate molecular disruptions that traditional techniques, such as ELISA and qRT-PCR, fail to fully elucidate. Considering these limitations, next-generation whole-transcriptome sequencing offers a powerful approach to uncover complex molecular changes [38,39,40,41]. To broaden our understanding of the molecular pathways involved in LCY-induced toxicity, we used this technology to analyze global transcriptomic changes in honey bees following chronic larval LCY exposure. Differentially expressed genes (DEGs) were identified and validated using qRT-PCR, followed by functional enrichment analyses to uncover the mechanistic basis of LCY-induced toxicity.

## 2. Materials and Methods

### 2.1. Larval Exposure to LCY

#### 2.1.1. In Vitro Rearing of First-Instar Honey Bee Larvae

First-instar honey bee (*Apis mellifera*) larvae used in the experiments were obtained from an experimental apiary at the National Institute of Agricultural Sciences, Rural Development Administration, Republic of Korea (35.591° N, 126.278° E). The colonies were maintained in a controlled environment for four weeks prior to the study, ensuring that no chemical substances were applied to the hives during this period. Procedures for larval collection, grafting, feeding, and experimental conditions followed established protocols [11,12]. Briefly, to obtain age-matched larvae, the queen was confined to an empty comb for 24 h to induce egg laying. After 72 h, combs were transferred to the laboratory, and first-instar larvae were grafted into sterile 48-well plates (#32024, SPL, Pocheon-si, Republic of Korea) and incubated at 35 °C, 95 ± 5% relative humidity. The larval diet, consisting of royal jelly, glucose, fructose, yeast extract, and distilled water, was freshly prepared, stored at 4 °C, and brought to 35 °C prior to feeding. For detailed protocols, refer to the Supplementary File.

#### 2.1.2. Chronic Sublethal Larval LCY Exposure, and Collection of Larval and Adult Honey Bee Samples

LCY, with a purity of 95%, sourced from Sigma (St. Louis, MO, USA), was initially prepared in acetone and mixed with the diet to obtain the desired concentration. Honey bee larvae were subjected to LCY in accordance with the protocols of previous studies [11,12] (see Supplementary Files for more details). The dosage of LCY chosen for larval exposure was 0.004 µg a.i./larva, representing one-tenth of the chronic LD_50_ value as previously established in our laboratory [11]. From days 3 to 6 post-grafting, each larva was exposed to 0.004 µg LCY/larva. The acetone concentration was maintained at 0.5% in the LCY exposure group. The LCY exposure was performed in triplicate, with each replicate containing 36 larvae. In the control group, the larvae were fed a diet containing 0.5% acetone. Dead larvae were removed daily. Larvae were collected on day 8 after grafting, and newly emerged adult bees were collected from day 19. For each experimental condition, three replicate samples were collected, with each sample consisting of three individual larvae or adults. The samples were rapidly frozen in liquid nitrogen and stored at –80 °C. Separate sets of samples were collected for transcriptomic analysis (n = 3) and qRT-PCR validation (n = 3). In total, four experimental groups were established based on treatment type and developmental stage: the solvent-treated larvae group (SLG), the solvent-treated adult group (SAG), the LCY-treated larvae group (LLG), and the LCY-treated adult group (LAG).

### 2.2. Transcriptome Analysis

#### 2.2.1. cDNA Library Construction

RNA isolation, RNA quantification, RNA integrity assessment, and cDNA library construction followed previously established methodologies [42], with comprehensive procedures provided in the Supplementary File. Briefly, total RNA was extracted using the RNeasy^®^ Mini Kit (#74106; QIAGEN, Hilden, Germany). RNA quality was confirmed using an RNA Screentape (#5067-5576; Agilent, Santa Clara, CA, USA), and samples with an RNA integrity number (RIN) >7 were used for cDNA library preparation.

The indexed cDNA libraries were subjected to sequencing on the NovaSeq platform by Illumina, Inc. (San Diego, CA, USA) using a paired-end setup (2 × 100 bp). To improve data quality, adapter sequences were removed, and low-quality bases were trimmed employing Trimmomatic v0.38 [43]. Subsequently, the cleaned reads were mapped to the *A. mellifera* genome (Amel_HAv3.1) utilizing HISAT v2.1.0, which was developed upon the frameworks of HISAT and Bowtie2 [44]. After alignment, reads were processed with SAMtools v1.9 [45]. Transcript assembly and quantification, including raw counts, FPKM, and TPM, were performed with StringTie v2.1.3b [46].

#### 2.2.2. DEG Filtering

DESeq2 v1.24.0 [47] was used to analyze differential gene expression, with raw count data serving as input. During quality control, genes with nonzero counts across all replicates of at least one group were retained. Principal component analysis and multidimensional scaling plots were generated to assess sample clustering and expression pattern consistency. To account for differences in library size, relative log expression normalization was applied. Differential expression analysis was performed using DESeq2’s negative binomial Wald test, yielding fold changes (FCs) and *p*-values [47]. To minimize false positives, *p*-values were adjusted using the Benjamini–Hochberg method for false discovery rate control. Genes were considered significantly differentially expressed if they met the criteria of |FC| ≥ 1.5 and raw *p*-value < 0.05. Hierarchical clustering of significant genes was carried out on log-transformed data, employing the Euclidean distance as the metric and complete linkage as the clustering method.

#### 2.2.3. DEG Enrichment Analysis

Functional annotation and gene enrichment analyses of significant genes were performed using gProfiler [48] by referencing the Gene Ontology (GO) and Kyoto Encyclopedia of Genes and Genomes (KEGG) pathway databases. Statistical significance was assessed using a one-sided hypergeometric test, and *p*-values were adjusted using the Benjamini–Hochberg correction to control for false discovery rate.

### 2.3. DEG Validation

RNA extraction, cDNA synthesis, and qRT-PCR were conducted as previously described [42]. Briefly, RNA was extracted from 50 mg larval or adult tissue using TRIzol Plus (#PG1117; Progen Life Sciences, New York, NY, USA) and the Direct-zol RNA Miniprep Kit (#R2052; Zymo research, Irvine, CA, USA), following the manufacturer’s protocols. RNA quality was verified using a Nanodrop 2000 (Thermo Fisher Scientific, Waltham, MA, USA), and 1500 ng of RNA was reverse-transcribed into cDNA using a kit (#K2205; Bioneer, Daejon, Republic of Korea). qRT-PCR analyses were conducted using the K6251 kit from Bioneer (Daejeon, Republic of Korea) on a CFX96 Dx system (Bio-Rad, Hercules, CA, USA). The fold differences in expression were determined via the 2^−ΔΔCT^ method [49], utilizing the average CT values from three housekeeping genes (*RPL13a* (*LOC108003218*)*, GAPDH* (*LOC108001468*)*,* and *RPS5* (*LOC107998344*)) for improved normalization. For detailed protocols and oligonucleotide primer sequences (Table S1), refer to the Supplementary File. Statistical analysis of qRT-PCR data was conducted using the Mann–Whitney U test in GraphPad Prism software (version 6, GraphPad Software, Boston, MA, USA).

A schematic overview of the workflow, including larval grafting, LCY exposure, staged sampling, and transcriptome analysis, is provided in Figure S1.

## 3. Results

### 3.1. DEGs in Honey Bee Larvae and Adults Exposed to LCY

**Figure 1:**
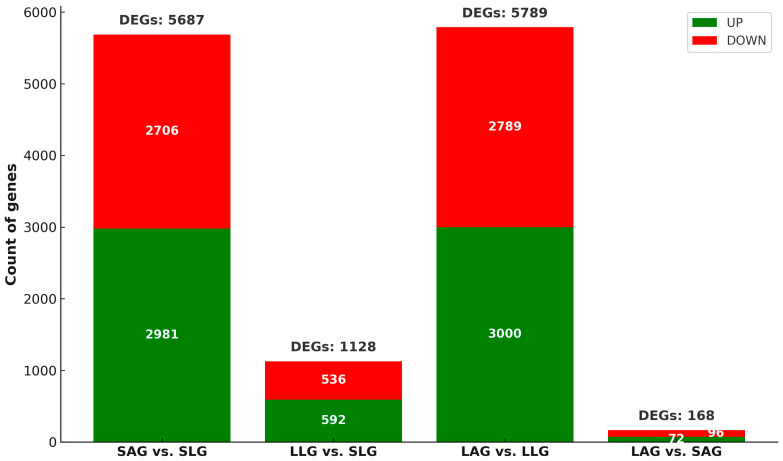
Differential gene expression across experimental conditions. The bar chart illustrates the number of differentially expressed genes (DEGs) (|FC| ≥ 1.5, *p* < 0.05), classified as upregulated (green) or downregulated (red), across four comparisons: SAG vs. SLG, LLG vs. SLG, LAG vs. LLG, and LAG vs. SAG. SLG (solvent-treated larvae group), SAG (solvent-treated adult group), LLG (LCY-treated larvae group), and LAG (LCY-treated adult group).

To identify DEGs in response to chronic sub-lethal LCY exposure, paired-end transcriptome sequencing was conducted using the Illumina platform across the four experimental groups: SLG, SAG, LLG, and LAG. Overall, 56.21 million raw reads were generated. After quality control, 97.47% of the reads were retained as clean reads, yielding an average of 54.79 million clean reads across all samples (Table 1). The guanine and cytosine (GC) content of the clean reads was approximately 38.43%, and the quality scores were notably high, with Q20 at 98.65% and Q30 at 97.70%. The high-quality reads were mapped to the *A. mellifera* reference genome Amel_HAv3.1 (NCBI_20180910), achieving an average alignment rate exceeding 97.16% across all sample groups. At an FC ≥ 1.5 and a raw *p*-value < 0.05, the comparisons of SAG vs. SLG, LAG vs. LLG, LLG vs. SLG, and LAG vs. SAG yielded 5687, 5789, 1128, and 168 DEGs, respectively (Figure 1). The list of DEGs across all comparisons is included in Supplementary File S1. Heat maps were constructed using two-way hierarchical clustering based on the Z-scores of log2-transformed normalized values, allowing visualization of gene expression similarities and differences across samples (Figure S2). Volcano plots were generated for each comparison (Figure S3), identifying significantly upregulated and downregulated genes, with the most pronounced changes appearing in the upper-right (upregulated) and upper-left (downregulated) regions.

### 3.2. Distinct and Overlapping DEGs in LCY-Exposed Honey Bee Larvae and Adult Groups

**Figure 2:**
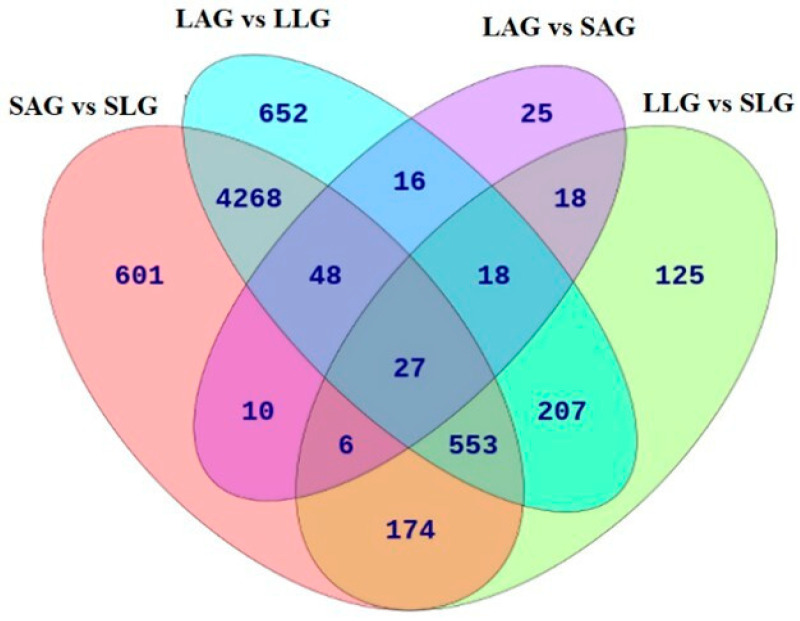
Venn diagram depicting shared and unique DEGs among experimental groups. The Venn diagram displays the unique and shared DEGs among the following comparisons, offering insights into gene expression alterations across different treatments: SAG vs. SLG, LAG vs. LLG, LLG vs. SLG, and LAG vs. SAG. SLG (solvent-treated larvae group), SAG (solvent-treated adult group), LLG (LCY-treated larvae group), and LAG (LCY-treated adult group).

A Venn diagram was generated to examine the overlap and distinctiveness of the DEGs among the experimental groups (Figure 2). The analysis identified 4896 DEGs shared between the SAG vs. SLG and LAG vs. LLG comparisons, emphasizing their essential roles in the larval-to-adult transition and normal honey bee development. In addition, 791 DEGs were specific to the SAG vs. SLG comparison, whereas 893 DEGs were exclusive to the LAG vs. LLG comparison.

Following LCY exposure, 125 distinct DEGs were identified in larvae, including genes linked to stress response (e.g., *hsp70*, *cyp6as14*) and neurodevelopment (e.g., *map2*, *Sog*). In adults, 25 unique DEGs were identified, such as glucose dehydrogenase [*FAD*, quinone], and deleted in autism protein 1 homolog, which are implicated in energy metabolism and neural function. A complete list of shared and condition-specific DEGs is provided in Supplementary File S2.

### 3.3. GO Enrichment of Significant DEGs

GO enrichment analysis of the significant DEGs was conducted to categorize them into three groups: biological processes (BPs), cellular components (CCs), and molecular functions (MFs).

#### 3.3.1. LLG vs. SLG

**Figure 3:**
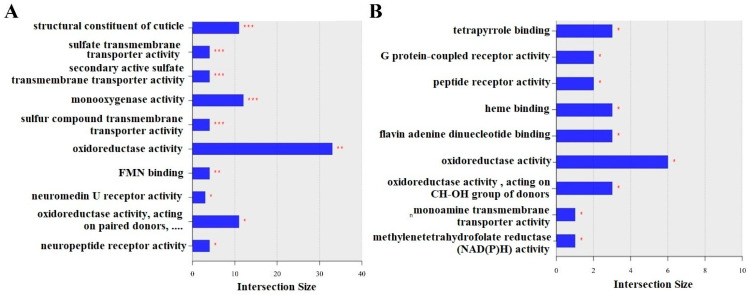
Top enriched GO terms for molecular function in DEGs between treatment groups. (**A**) Top 10 enriched MFs in LLG vs. SLG. (**B**) Top nine enriched BPs in LAG vs. SAG. SLG: solvent-treated larvae group; SAG: solvent-treated adult group; LLG: LCY-treated larvae group; LAG: LCY-treated adult group. Statistical significance: * *p* < 0.05, ** *p* < 0.01, *** *p* < 0.001.

The top enriched MFs included structural constituents of the cuticle, sulfate transmembrane transporter activity, secondary active sulfate transmembrane transporter activity, monooxygenase activity, sulfur compound transmembrane transporter activity, oxidoreductase activity, FMN binding, neuromedin U receptor activity, oxidoreductase activity acting on paired donors, and neuropeptide receptor activity (Figure 3A). These enriched functions point to significant alterations in cuticle formation, transport systems, redox reactions, and signaling pathways, suggesting a potential impact of LCY on larval development and physiology. Notably, genes related to cuticle structure (e.g., *cpr* family genes) and sulfate transporters (e.g., *LOC413816*, *LOC410507*), as well as multiple detoxification-related genes such as *Cyp314a1* and *nos*, were significantly affected. The genes related to these terms are listed in Table S2.

#### 3.3.2. LAG vs. SAG

The top enriched MFs included tetrapyrrole binding, G protein-coupled peptide receptor activity, peptide receptor activity, heme binding, flavin adenine dinucleotide binding, oxidoreductase activity, oxidoreductase activity acting on the CH–OH group of donors, monoamine transmembrane transporter activity, and methylenetetrahydrofolate reductase (NAD(P)H) activity (Figure 3B). These functions indicate disruptions in binding activities, redox reactions, and signaling pathways, which may have lasting developmental consequences extending into adulthood, potentially influencing neural function and behavior. Notably, genes involved in redox reactions and cofactor binding (e.g., *LOC408452*, *LOC551179*, *LOC551044*) and neuropeptide signaling (e.g., *akhr*, *LOC411672*) were differentially expressed. Additionally, *LOC100578936*, associated with folate metabolism, may reflect altered one-carbon metabolism under LCY exposure. These results suggest that LCY exposure changes neurometabolic gene expression, potentially leading to lasting behavioral and neurological issues in adult bees. The list of genes for these terms is included in Table S3.

#### 3.3.3. LAG vs. LLG and SAG vs. SLG

A significant number of DEGs overlapped in the SAG vs. SLG and LAG vs. LLG comparisons (Figure 2), which led to shared GO categories.

In the CCs category, eight of the top ten terms were shared across both comparisons (Figure S4), highlighting the key cellular structures essential for mitochondrial function and energy homeostasis. While the respirasome and proton-transporting two-sector ATPase complexes were exclusive to the SAG vs. SLG comparison, the cytoplasmic and organelle membranes were specific to the LAG vs. LLG comparison. These findings suggest that LCY exposure may differentially affect mitochondrial integrity and cellular compartmentalization during honey bee development.

In the MFs category, eight of the top ten terms overlapped between the comparisons (Figure S5), highlighting the importance of membrane transport and structural integrity in honey bees. While proton transmembrane transporter activity and monoatomic ion transmembrane transporter activity were unique to the SAG vs. SLG comparison, electron transfer activity and oxidoreductase activity were specific to the LAG vs. LLG comparison. This indicates that LCY exposure may selectively alter membrane transport processes and redox homeostasis, potentially disrupting cellular functions during development.

In the BPs category, eight of the top ten terms were common across both comparisons (Figure S6), emphasizing the pathways that are critical for cellular energy balance, essential functions, and honey bee growth and development. Notably, cellular respiration and ATP synthesis coupled electron transport were specific to the SAG vs. SLG comparison, whereas purine-containing compound metabolic process and nucleotide metabolic process were unique to the LAG vs. LLG comparison. This suggests that LCY exposure disrupts cellular energy production and genetic material metabolism, potentially affecting honey bee development from larvae to adults. The lists of genes associated with these CC, MF, and BP terms are shown in Tables S4–S9.

### 3.4. KEGG Enrichment Analysis

The enriched KEGG pathways were grouped into six major categories, and a full list of identified biological pathways is provided in Figure S7.

#### 3.4.1. LLG vs. SLG

**Figure 4:**
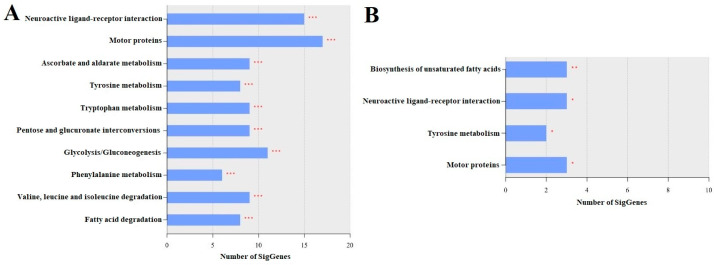
Top 10 enriched KEGG pathways across different transcriptome comparisons. The panels represent the most enriched KEGG pathways identified in the following comparisons: (**A**) LLG vs. SLG and (**B**) LAG vs. SAG. SLG: solvent-treated larvae group; SAG: solvent-treated adult group; LLG: LCY-treated larvae group; LAG: LCY-treated adult group. Statistical significance is indicated as follows: * *p* < 0.05, ** *p* < 0.01, *** *p* < 0.001.

The top 10 enriched pathways in the LLG included neuroactive ligand–receptor interaction; motor proteins; ascorbate and aldarate metabolism; tyrosine metabolism; tryptophan metabolism; pentose and glucuronate interconversions; glycolysis/gluconeogenesis; phenylalanine metabolism; valine, leucine, and isoleucine degradation; and fatty acid degradation (Figure 4A). These results suggest that exposure to LCY causes significant disruptions in nervous system function, energy metabolism, oxidative stress response, and overall metabolic health, potentially affecting the normal growth and development of larvae. Specifically, LCY exposure appears to dysregulate genes involved in nervous system function (e.g., *nAChRa9*, *dop1*, *sifr*), energy metabolism (e.g., *tpi*, *LOC408559*), oxidative stress response (e.g., *LOC408650*, *LOC411140*), and overall metabolic health (e.g., *LOC551465*, *LOC551837*), potentially impacting larval development. A list of the genes for each pathway is presented in Table S10.

#### 3.4.2. LAG vs. SAG

The top enriched pathways in the LAG included the biosynthesis of unsaturated fatty acids, neuroactive ligand–receptor interaction, tyrosine metabolism, and motor proteins (Figure 4B). These enriched pathways suggest that LCY exposure may disrupt lipid metabolism, neural signaling, and muscle function, potentially impairing essential behaviors and colony health in adult honey bees. Specifically, genes such as *LOC727166* and *LOC724226*, involved in fatty acid synthesis, may influence energy storage and flight performance, whereas *akhr* and *nAChRa9*, linked to neuropeptide signaling and sensory function, and *LOC551465*, associated with neurotransmitter production, indicate neural impacts. Additionally, motor-related genes such as *LOC100578129* and *LOC551109* affect muscle function. Changes in the expression levels of these genes suggest that LCY exposure could impair individual performance and, cumulatively, affect colony-level stability and productivity. A list of genes that belong to each enriched pathway is presented in Table S11.

#### 3.4.3. LAG vs. LLG and SAG vs. SLG

KEGG pathway analysis revealed key developmental pathways in both control and LCY-treated groups. In the controls (SAG vs. SLG), 10 pathways were enriched, reflecting metabolic and regulatory processes (Figure S8A). Upon LCY exposure (LAG vs. LLG), eight of these pathways remained enriched, whereas peroxisome function and nucleocytoplasmic transport were uniquely affected (Figure S8B), suggesting cellular stress and impaired molecular transport. In addition, neuroactive ligand–receptor interaction and pyruvate metabolism, which were enriched in the controls, were absent in the LCY-treated group, indicating disruptions in neurotransmission and energy balance. These results suggest that LCY exposure alters metabolic and signaling pathways beyond normal developmental changes, with potential long-term effects on physiology and behavior. The list of genes of each enriched pathway is included in Tables S12 and S13.

### 3.5. Transcriptome Data Validation

**Figure 5:**
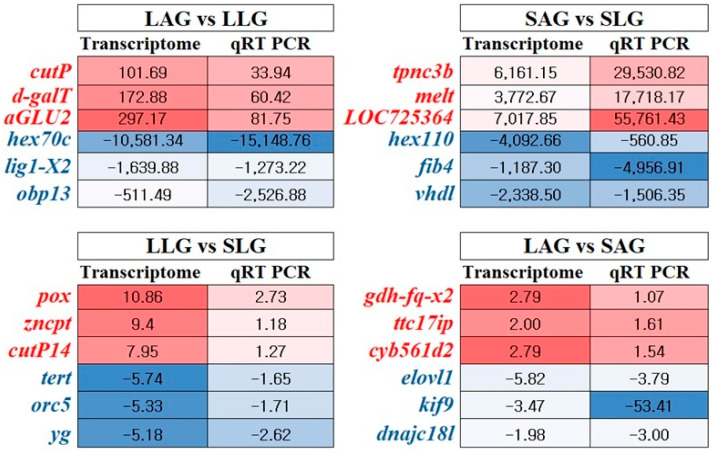
Comparison of gene expression data between transcriptome analysis and qRT-PCR across honey bee sample comparisons. The graphs illustrate the fold changes (FCs) in expression levels of selected genes, with qRT-PCR results displayed alongside corresponding transcriptome data. SLG (solvent-treated larvae group), SAG (solvent-treated adult group), LLG (LCY-treated larvae group), and LAG (LCY-treated adult group). Gene abbreviations: *cutp* (Cuticular protein), *d-galt* (D-galactonate transporter), *aglu2* (Alpha-glucosidase 2), *hex70c* (Hexamerin 70c), *lig1-x2* (DNA ligase 1, variant X2), *obp13* (Odorant-binding protein 13), *pox* (Peroxidase), *zncpt* (Zinc carboxypeptidase), *cutp14* (Cuticular protein 14), *tert* (Telomerase reverse transcriptase), *orc5* (Origin recognition complex subunit 5), *yg* (Yellow-g), *gdh-fq-x2* (Glucose dehydrogenase [FAD, quinone], variant X2), *melt* (Melittin), *LOC725364* (Flightin), *hex110* (Hexamerin 110), *fib4* (Silk fibronin 4), *vdhl* (Larva-specific very high-density lipoprotein), *ttc17ip* (TTC17-interacting protein), *cyb561d2* (Cytochrome b561 domain-containing protein 2), *elovl1* (Elongation of very long-chain fatty acids protein 1), *kif9* (Kinesin-like protein 9), and *dnajc18l* (DNAJ homolog subfamily C member 18-like). Blue indicates downregulation, and red indicates upregulation, of gene expression levels in LCY-treated samples relative to solvent-treated controls.

Three upregulated and three downregulated genes from each comparison were randomly selected and analyzed via qRT-PCR to validate the reliability of the transcriptome data. All genes tested using qRT-PCR exhibited similar trends to those observed in the transcriptome analysis data (Figure 5). Selected genes and their expression statistics are provided in Table S14. The direction and trend of gene expression were found to be similar in both qRT-PCR and transcriptome analysis. A scatter plot showed a strong correlation between transcriptome and qRT-PCR FCs (R^2^ = 0.658) (Figure S9), confirmed by Pearson (r = 0.811, *p* < 0.0001) and Spearman (ρ = 0.951, *p* < 0.0001) analyses.

## 4. Discussion

The adverse effects of LCY on honey bees are well-documented, including increased mortality, morphological deformities in larvae, and behavioral disruptions in adults [11,14,15,31,32,50]. We employed next-generation transcriptome analysis to investigate LCY’s molecular impact on bees, revealing that it interferes with crucial signaling pathways in both larvae and adults. In larvae, LCY disrupted carbohydrate and fatty acid metabolism and pathways related to structural integrity, thereby compromising energy homeostasis and normal growth. In adults, persistent neurotoxicity was evident, with enrichment in pathways related to neuroactive ligand–receptor interactions, tyrosine metabolism, and synaptic transmission impairments. Despite these differences, both groups exhibited systemic disruptions in oxidative stress and detoxification pathways, highlighting the pervasive impact of LCY toxicity. Collectively, these molecular alterations reinforce the link between LCY exposure and previously reported developmental and behavioral impairments in honey bees.

### 4.1. Metabolic and Neurodevelopmental Impacts of LCY Exposure in Honey Bee Larvae

Chronic exposure to LCY may disrupt crucial metabolic, neurological, oxidative, and developmental processes in larvae by dysregulating genes that are vital for energy metabolism, neurotransmitter biosynthesis, structural integrity, and detoxification. These molecular disruptions align with previously observed phenotypic and biochemical impairments, contributing to long-term developmental defects, diminished adult fitness, and overall colony decline [11,15,32,50].

Metabolic dysfunction appears to be a notable consequence of LCY exposure, as demonstrated by widespread transcriptional changes in key mitochondrial enzymes, detoxification systems, and energy metabolism pathways [51,52,53,54]. KEGG enrichment of glycolysis/gluconeogenesis, pentose and glucuronate interconversions, and fatty acid degradation pathways suggests metabolic stress and a shift in energy sourcing. Notably, the upregulation of core glycolytic genes including hexokinase-1, fructose-bisphosphate aldolase, mitochondrial enolase superfamily member 1, and glyceraldehyde-3-phosphate dehydrogenase supports enhanced glycolytic activity [52,53,54]. Additionally, the upregulation of glucose dehydrogenase (FAD, quinone) and 6-phosphogluconate dehydrogenase suggests increased glucose and sugar derivative flux through glycolysis and the pentose phosphate pathway, likely supporting ATP and NADPH production to maintain redox balance under LCY-induced oxidative stress [52,53,54].

Elevated expressions of mitochondrial enzymes such as 2-oxoglutarate dehydro-genase, succinate semialdehyde dehydrogenase, aldehyde dehydrogenase, and L-threonine 3-dehydrogenase suggest an attempt to preserve energy metabolism and redox balance through enhanced TCA cycle function and amino acid catabolism [53,55,56,57,58]. Concurrent upregulation of short/branched chain-specific acyl-CoA dehydrogenase and isovaleryl-CoA dehydrogenase implies increased fatty acid and branched-chain amino acid oxidation as an alternative energy source [59,60]. Additionally, enrichment in the fatty acid degradation pathway along with the upregulation of NPC intracellular cholesterol transporters 1 and 2, lipase 3, and pancreatic triacylglycerol lipase-like suggests mobilization of lipid reserves as part of a broader metabolic adjustment [59,60]. This coordinated activation of glycolysis, lipid, and amino acid catabolism likely reflects a flexible strategy to meet elevated energetic demands imposed by LCY exposure during larval development.

Furthermore, detoxification pathways appear strongly activated, as evidenced by pronounced upregulation of cytochrome P450 family members (*cyp6a14, cyp314A1, cyp6k1, cyp9e2*) and various oxidoreductases, including short-chain dehydrogenase/reductase and retinal dehydrogenase 1, reflecting intensified metabolic processing of LCY and oxidative byproducts [51,61]. However, the marked downregulation of UDP-glucuronosyltransferase 1–3 and components of the mitochondrial electron transport chain (cytochrome b-c1 complex subunit 9, NADH–cytochrome b5 reductase 1) indicates impaired phase II detoxification and compromised mitochondrial respiration, potentially reducing ATP generation and exacerbating oxidative stress [62]. The overexpression of peroxidase points to a heightened oxidative burden, as this enzyme plays a crucial role in scavenging reactive oxygen species (ROS) generated during metabolic stress [61,63]. These observations were further supported by biochemical evidence showing reduced ATP production, increased lipid peroxidation (elevated MDA levels), and elevated oxidative stress markers in LCY-exposed larvae [11,50]. Taken together, our DEG data indicate that LCY exposure triggers a state of metabolic reprogramming in honey bee larvae, involving coordinated activation of glycolysis, the pentose phosphate pathway, amino acid catabolism, and lipid β-oxidation to meet increased energy and redox demands under oxidative stress. However, despite these adaptive responses, persistent mitochondrial dysfunction, impaired phase II detoxification, and elevated oxidative burden suggest that the cumulative stress exceeds the larvae’s metabolic capacity, ultimately leading to adverse developmental outcomes such as reduced pupation success, lower adult emergence rates, and increased morphological deformities [11,32].

Thus, oxidative stress likely represents one of the key mechanisms of LCY toxicity. This is supported by KEGG enrichment in the ascorbate and aldarate metabolism pathway and GO terms such as oxidoreductase activity and FMN binding, all of which are involved in ROS metabolism and detoxification [61]. Notably, an 11-fold induction of peroxidase, a key antioxidant enzyme, indicates an acute oxidative response in LCY-exposed larvae, likely aimed at neutralizing excess hydrogen peroxide and related ROS [61]. This response is further supported by the upregulation of glucose oxidase and hydroxyacid oxidase 1, both of which generate or metabolize ROS as part of redox homeostasis [61]. Additionally, the increased expression of pyridoxine/pyridoxamine 5′-phosphate oxidase and pyridoxine-5′-phosphate oxidase, both essential for vitamin B6 metabolism, suggests an enhanced requirement for pyridoxal phosphate, a coenzyme involved in antioxidant processes and amino acid metabolism [61,64]. Despite these apparent protective mechanisms, LCY exposure is associated with the downregulation of mitochondrial RNA pseudouridine synthase and DNAJ homolog subfamily C member 18-like, implying mitochondrial dysfunction and impaired protein-folding capacity, which could compromise ATP production and activate apoptosis pathways [11,32,65]. Thus, while transcriptional upregulation of redox-related genes reflects an effort to mitigate LCY-induced oxidative damage, it may be insufficient to fully prevent mitochondrial impairment and cell death. These findings warrant further investigation into the balance between protective antioxidant responses and irreversible oxidative damage under LCY exposure.

Additionally, LCY exposure significantly disrupts sulfur metabolism in honey bee larvae, as evidenced by enriched GO terms such as sulfate transmembrane transporter activity and oxidoreductase activity, along with differential expression of key sulfur-related genes. Sulfur metabolism is vital for protein folding, antioxidant defense, and enzyme activity, and its dysregulation can impair detoxification efficiency and exacerbate cellular stress [61,63]. The transcriptional changes upon LCY exposure suggest a dual response involving the compensatory upregulation of protective genes alongside the suppression of essential mitochondrial functions. Notably, the upregulation of sodium-independent sulfate anion transporter isoforms and bifunctional 3′-phosphoadenosine 5′-phosphosulfate synthase 2 indicates an attempt to increase sulfate import and activation to meet the heightened sulfur demand during toxic stress [63]. The induction of homocysteine S-methyltransferase YbgG and 3-ketoacyl-CoA thiolase further supports an adaptive metabolic response aimed at maintaining methylation balance and enhancing lipid catabolism [63]. Despite these compensatory responses, the observed molecular disruptions suggest that larvae may have a limited capacity to detoxify LCY effectively [11]. However, these potentially protective mechanisms are undermined by the downregulation of key mitochondrial sulfur-handling and redox-related proteins, including iron-sulfur cluster co-chaperone protein HscB, S-adenosylmethionine mitochondrial carrier protein, and CDGSH iron-sulfur domain-containing protein 3, which are vital for cofactor biogenesis, electron transport, and oxidative phosphorylation [56,57]. Collectively, these changes indicate that although LCY-exposed larvae activate sulfur-based detoxification and metabolic responses, concurrent disruption of mitochondrial sulfur handling and redox balance likely limits their effectiveness, leading to persistent stress and overall reduction in detoxification capacity.

Neurotoxicity due to LCY exposure was evident through disruptions in the metabolism of tyrosine, tryptophan, and phenylalanine, which are essential for neurotransmitter biosynthesis [66]. These changes potentially alter dopamine and serotonin production, thereby affecting motor control, learning, and stress responses and likely contributing to the observed impairments in learning, olfactory memory, and foraging efficiency [14,15,31,66,67]. Such behavioral disruptions, if persistent, can potentially contribute to colony collapse [68,69]. Notably, the downregulation of leucine-rich repeat transmembrane neuronal protein 4-like and glutamate receptor ionotropic delta-2 suggests impaired excitatory signaling and reduced synaptic responsiveness [15,70]. Suppressed expression of acetylcholine receptor subunit alpha-10 and dopamine receptor D1 indicates further disruption of cholinergic and dopaminergic pathways crucial for cognitive and motor control [69,71,72,73]. Simultaneously, reduced expression of tropomyosin-2-like compromises neuronal structure and further impairs neuronal function [74]. Conversely, the upregulation of neurofilament heavy polypeptide, nicotinic acetylcholine receptor (alpha9), sensory neuron membrane protein 2, metabotropic glutamate receptor 7, leucine-rich repeat neuronal protein 1, and dopa-mine N-acetyltransferase reflects compensatory efforts to preserve synaptic function and neurotransmitter balance [69,72,75,76,77]. The enrichment in neuropeptide and neuromedin U receptor activity further supports an adaptive response by larvae to counteract the synaptic dysfunction induced by LCY [78]. However, these responses may be inadequate to fully mitigate toxicity. Notably, behavioral impairments such as disorientation and abnormal foraging have also been reported in adult honey bees directly exposed to LCY, raising the possibility that similar molecular disruptions may underlie these outcomes despite differences in exposure stage [32,69].

Structural integrity appears compromised in LCY-exposed larvae, as evidenced by GO terms related to cuticle structure. Notably, the upregulation of cuticular proteins 13 and 14, which are integral to exoskeletal formation and rigidity [79,80], likely reflects a compensatory response to LCY-induced stress aimed at reinforcing the exoskeleton. However, despite this apparent molecular adaptation, LCY exposure results in malformed wings and antennae, along with reduced body size [11]. This discrepancy suggests that the protective upregulation of cuticular proteins may be insufficient to fully preserve structural integrity. Notably, the substantial downregulation of keratin-associated protein 19-2, along with collagen alpha-1(I) and collagen alpha-2(IV) chain-like, suggests a weakening of the extracellular scaffold necessary for tensile strength and elasticity of the cuticle [81,82]. These findings suggest that the downregulation of keratin-associated protein 19-2 and collagen alpha chains weakens the cuticle’s supporting structure, likely reducing the effectiveness of the upregulated cuticular proteins. Nonetheless, this hypothesis requires further functional validation.

Overall, LCY exposure triggers widespread transcriptional changes in honey bee larvae, impacting neural activity, metabolic processes, structural development, and detoxification pathways. While several upregulated genes suggest the activation of compensatory responses, the overall pattern of dysregulation points to reduced physiological resilience and developmental fitness under LCY stress.

### 4.2. Persistent Neurobehavioral and Metabolic Dysfunctions in Adult Honey Bees Exposed to LCY

Chronic exposure to LCY during the larval stage appears to have lasting molecular effects that persist into adulthood, affecting key pathways related to neurobehavioral function and metabolism. The data highlight a complex interplay between disrupted neural signaling, impaired metabolic processes, and compromised detoxification mechanisms. These alterations may contribute to energy imbalances and cognitive vulnerabilities, potentially impacting individual fitness and overall colony stability [11,14,15,31,32,50].

Neurotoxicity may be a major and persistent toxic effect of LCY, particularly via the disruption of the neuroactive ligand–receptor interaction pathway (NLRP). Since the NLRP regulates synaptic transmission and neural communication, its disruption could impair learning and foraging behaviors in bees [31,32,83]. Furthermore, GO analysis highlighted the enrichment of G protein-coupled peptide receptor activity and peptide receptor activity, both of which play critical roles in neuromodulation, sensory perception, and hormone signaling, and are key components of the NLRP [84,85]. This suggests that LCY exposure not only disrupts neurotransmission but also alters stress and sensory response mechanisms.

Additionally, the upregulation of neuropeptides, including CAPA receptor-like protein, the nicotinic acetylcholine receptor (nAChR) alpha9 subunit, and the adipokinetic hormone receptor, suggests that LCY exposure alters neural signaling pathways, stress responses, and energy metabolism [71,84]. Notably, the upregulation of the nAChR alpha9 subunit, a key mediator of cholinergic neurotransmission, serves as a compensatory response to synaptic disruption but may heighten neural excitability and impair memory formation [11,71]. These molecular changes, indicative of disruptions in the NLRP, align with the observed learning deficits, as LCY-exposed bees exhibit poor performance in proboscis extension response learning assays [32]. Furthermore, the effect of LCY on peptide receptor activity may alter sensory processing and neurodevelopment, potentially explaining the disorientation and foraging inefficiencies observed in the exposed bees. This aligns with reports of a 90% decline in foraging activity following LCY exposure [15,31].

LCY exposure also disrupted tyrosine metabolism, a crucial pathway for neurotransmitter synthesis. The increased activity of homogentisate 1,2-dioxygenase, an enzyme essential for tyrosine breakdown, suggests either tyrosine depletion or a shift in metabolic flux. Furthermore, reduced aromatic-L-amino-acid decarboxylase activity indicates decreased biogenic amine production, which may exacerbate synaptic transmission deficits caused by LCY’s direct impact on the NLRP. KEGG pathway enrichment analysis confirms that LCY specifically targets tyrosine metabolism and may reduce the availability of neurotransmitter precursors [66,86].

These metabolic disturbances, combined with LCY’s direct effects on neuropeptide and acetylcholine receptors, likely contribute to the observed 90% decline in foraging activity [31] by impairing sensory processing, neuromodulation, and energy metabolism. Thus, LCY-induced neurotoxicity appears to result from both direct interference with neural receptors and indirect disruption of neurotransmitter balance via altered tyrosine metabolism. These pathways are also closely linked to mitochondrial function, ATP production, and detoxification processes, highlighting oxidative stress as a central mechanism in LCY toxicity [54,58,87]. The upregulation of cytochrome b5, glucose dehydrogenase (FAD, quinone), and homogentisate 1,2-dioxygenase suggests that bees activate antioxidant defense mechanisms to mitigate ROS accumulation [87]. However, the downregulation of NADH dehydrogenase subunit 6 and NADH-cytochrome b5 reductase 1 indicates mitochondrial dysfunction, energy deficit, and impaired detoxification capacity, which may contribute to premature senescence and colony collapse [15,87]. These findings align with biochemical evidence of elevated lipid peroxidation and reduced antioxidant enzyme activities following LCY exposure [11,15,50].

Metabolic disruptions, particularly in lipid biosynthesis and transport, may further exacerbate LCY-induced neurotoxicity. GO analysis highlighted the enrichment in monoamine transmembrane transporter activity, which is essential for regulating dopamine and serotonin levels [88,89]. These neurotransmitters play critical roles in behavioral responses, stress adaptation, reproduction, and cognitive function [89,90,91]. The downregulation of NAD(P)H and monoamine-related transport genes suggests potential impairments in neurotransmitter metabolism, which may contribute to mood dysregulation, abnormal locomotion, and reduced foraging motivation [15,31,32]. Additionally, the observed enrichment of NAD(P)H activity is noteworthy because this enzyme is integral to DNA methylation and epigenetic regulation [92]. This suggests that LCY exposure influences gene expression beyond immediate physiological responses, potentially leading to long-term molecular and behavioral consequences [11,32,50].

The enrichment of the biosynthesis of unsaturated fatty acids pathway in LAG provides further insights into LCY-induced metabolic disruption. Downregulation of the elongation of very long-chain fatty acids protein 1 and acyl-CoA DeLCA(11) desaturase variant X3, alongside the upregulation of acyl-CoA DeLCA(11) desaturase variant X1, suggests impairments in membrane integrity, lipid storage, and stress resilience [15,93,94]. As unsaturated fatty acids are essential for cellular functions, hormone signaling, and immune defense, their disruption may weaken colony health, increase pathogen susceptibility, and delay development and behavior [11,15]. Additional metabolic disturbances were evident in enriched GO terms related to energy metabolism (FAD binding, heme binding, and oxidoreductase activity) and neurotransmitter function (monoamine transmembrane transporter activity) in LAG. Mitochondrial dysfunction, reflected in alterations to electron transport chain components, can directly affect fatty acid synthesis and vice versa [58]. Moreover, changes in lipid composition may disrupt the function of membrane-bound receptors, including G protein-coupled peptide receptors and nicotinic acetylcholine receptors, potentially exacerbating LCY’s neurotoxic effects [95]. Ultimately, the disruption of unsaturated fatty acid biosynthesis is a key factor linking metabolic dysfunction, oxidative stress, and neurotoxicity in LAG. Thus, the observed deficits in foraging activity, learning and memory, pollen and nectar storage, gut microbiota composition, and detoxification processes may collectively result from these widespread molecular disruptions [11,15,31,32].

Alterations in the detoxification pathways highlighted the limited ability of honey bees to metabolize LCY. The upregulation of cytochrome P450 9e2 and UDP-glucuronosyltransferase 2B17-like suggests an attempt to clear pesticide residues, whereas the downregulation of cytochrome P450 305a1 implies reduced LCY clearance efficiency [11,50]. As pyrethroids accumulate in tissues and disrupt multiple organ systems, these findings align with the systemic effects observed in adults exposed to LCY [96,97]. These molecular disruptions correspond with colony decline, reduced nectar collection, and weakened brood development, indicating prolonged pesticide retention and toxicity [15].

Developmental impairments in LCY-exposed honey bees suggest persistent structural and physiological deficiencies [11]. One notable alteration was the upregulation of cuticular protein 4, indicating potential disruption in exoskeleton formation, possibly due to LCY-induced cuticle damage or developmental stress [98]. Simultaneously, the downregulation of kinesin-like proteins (KIF9, KIN-7L, and K5A) suggests impairment of intracellular transport and structural maintenance, as these motor proteins are essential for cellular organization and function [99]. As intracellular transport is critical for proper cell growth, differentiation, and tissue integrity, such disruptions may contribute to the developmental abnormalities reported in LCY-exposed bees [11,99].

Endocrine-related disruptions in LAG were evident through the downregulation of juvenile hormone acid O-methyltransferase and methyl farnesoate epoxidase, suggesting dysregulation of hormonal signaling [100]. As these hormones regulate caste differentiation, reproduction, and colony organization, their disruption may lead to developmental impairments such as reduced pupation success, delayed emergence, and shortened adult lifespans [11,15]. Such effects may weaken colony stability and reproductive success, thereby contributing to population decline. Additionally, compromised immune and stress response pathways may increase susceptibility to environmental challenges. The upregulation of TOX high mobility group box family member 3-like suggests an adaptive immune activation response to LCY-induced physiological stress [101]. However, downregulation of the DNA repair protein RAD51 homolog 4 raises concerns about genomic instability, as impaired DNA repair capacity may increase vulnerability to DNA damage and long-term genetic impairments [102]. These interconnected molecular disruptions collectively threaten the health and resilience of honey bee colonies.

This study primarily presents transcriptomic analysis with qRT-PCR validation of selected DEGs, providing a solid foundation for understanding the molecular responses of honey bee larvae and adults to sublethal larval LCY exposure. However, transcriptome data inherently reflect gene expression changes at the RNA level and may not fully correspond to protein abundance or activity. Therefore, additional functional assays such as protein-level validation, enzymatic activity measurements, and gene-function studies are needed to further elucidate the mechanisms underlying LCY-induced phenotypic effects. Despite these limitations, the dataset offers valuable insights and serves as a critical resource for guiding future mechanistic research.

## 5. Conclusions

This study revealed significant transcriptomic disruptions in honey bee larvae and adults following LCY exposure, affecting key biological processes such as energy metabolism, neurotransmitter biosynthesis, oxidative stress responses, and detoxification pathways. These molecular alterations impair larval development, reduce pupation success, and compromise adult fitness, highlighting the systemic effects of LCY on honey bee physiology. Disruption of neuroactive ligand–receptor interactions and tyrosine metabolism suggests a potential link to the observed deficits in learning, foraging efficiency, and behavioral responses, while metabolic stress contributes to energy imbalances and oxidative damage. Structural deficiencies, such as weakened exoskeleton formation, may compromise survival. The limited detoxification capacity of honey bees underscores their vulnerability to LCY, as impaired enzymatic defenses appear insufficient to effectively mitigate toxicity. These findings provide molecular insights into the mechanisms underlying previously reported biochemical and behavioral impairments. Understanding these disruptions is crucial for refining pesticide risk assessments, developing targeted mitigation strategies, and promoting sustainable pest management practices to protect honey bee populations and maintain the ecological balance.

## Figures and Tables

**Table 1:** Summary of transcriptome data, including read counts, sequence quality, and mapping rates.

Samples	Total Reads	Clean Reads	GC (%)	Q20 (%)	Q30 (%)	Mapped Reads	Unmapped Rate (%)	Mapped Rate (%)
LAG-1	58,406,928	56,777,182	38.40	98.48	97.65	55,333,933	2.54	97.46
LAG-2	53,953,234	52,345,476	38.85	98.46	97.62	50,921,510	2.67	97.28
LAG-3	54,812,774	53,237,224	38.06	98.49	97.64	51,798,939	2.70	97.30
LLG-1	56,396,336	55,070,232	39.61	98.79	97.68	53,431,764	2.98	97.02
LLG-2	50,705,676	49,305,624	39.42	98.55	97.73	47,920,635	2.81	97.19
LLG-3	60,087,126	58,930,328	39.39	98.91	97.95	57,267,666	2.82	97.18
SAG-1	62,326,854	60,905,516	37.71	98.75	97.73	59,163,527	2.86	97.14
SAG-2	57,545,836	56,071,312	37.18	98.72	97.48	54,287,287	3.18	96.82
SAG-3	55,289,460	54,121,612	37.86	98.83	97.78	52,478,090	3.04	96.96
SLG-1	62,347,274	60,872,770	37.62	98.77	97.70	59,142,506	2.84	97.16
SLG-2	50,206,390	48,908,502	38.75	98.53	97.73	47,621,910	2.63	97.37
SLG-3	52,455,156	50,888,552	38.27	98.50	97.67	49,363,367	3.00	97.00

SLG, solvent-treated larvae group; SAG, solvent-treated adult group; LLG, LCY-treated larvae group; LAG, LCY-treated adult group; GC, guanine and cytosine content; Q20, proportion of bases with a quality score of 20 or higher; Q30, proportion of bases with a quality score of 30 or higher.

## Data Availability

The datasets generated and/or analyzed in this study are included in this paper and provided as Supplementary Information. Raw sequencing files used in this analysis are available upon request from the corresponding author.

## Supplementary Materials

The following supporting information can be downloaded at: https://www.mdpi.com/article/10.3390/insects16080833/s1, Figure S1: Schematic representation of the experimental workflow for LCY exposure and transcriptome analysis in *Apis mellifera*. First-instar larvae were grafted into 48-well plates (36 larvae per replicate) and reared at 35 °C and 95% relative humidity. From Day 3 to Day 6, larvae were chronically exposed to LCY (0.004 µg/larva; *n* = 3) or acetone control (0.5%; *n* = 3). Samples were collected at the larval stage (SLG: solvent-treated larvae group; LLG: LCY-treated larvae group; Day 8; three larvae per replicate) and adult stage (SAG: solvent-treated adult group; LAG: LCY-treated adult group; Day 19; three bees per replicate). Total RNA was extracted (RIN > 7), followed by cDNA library construction, Illumina paired-end sequencing (2 × 100 bp), read alignment to the *Apis mellifera* reference genome (*Amel_HAv3.1*), differential gene expression (DEG) analysis using DESeq2, Gene Ontology (GO) and Kyoto Encyclopedia of Genes and Genomes (KEGG) annotation, and qRT-PCR validation of selected DEGs. Figure S2: Comparative transcriptomic analyses of honey bee responses to lambda-cyhalothrin in honey bee larvae and adults. The heat map illustrates the differential expression of genes between four treatment comparisons: (A) SAG vs. SLG, (B) LAG vs. LLG, (C) LLG vs. SLG, and (D) LAG vs. SAG. The data are presented as z-scores of normalized log2-transformed expression values. Two-way hierarchical clustering was applied to organize the data. Gene expression differences are considered significant at a fold change of 1.5 with significance threshold *p*-values applied. SLG, solvent-treated larvae group; SAG, solvent-treated adult group; LLG, LCY-treated larvae group; LAG, LCY-treated adult group. Figure S3: Comparative transcriptomic analyses of honey bee responses to lambda-cyhalothrin in honey bee larvae and adults. Volcano plots illustrate the differential expression of genes between four treatment comparisons: (A) SAG vs. SLG, (B) LAG vs. LLG, (C) LLG vs. SLG, and (D) LAG vs. SAG. The data are presented as log2 fold changes against −log10 raw *p*-values. Gene expression differences are considered significant at a fold change (FC) of 1.5 and a raw *p*-value < 0.05, with upregulated genes shown in yellow and downregulated genes in blue. SLG, solvent-treated larvae group; SAG, solvent-treated adult group; LLG, LCY-treated larvae group; LAG, LCY-treated adult group. Figure S4: Top 10 cellular component terms of Gene Ontology (GO) functional analysis. (A) SAG vs. SLG and (B) LAG vs. LLG. Each bar represents the intersection size, with significance levels denoted by asterisks: a single asterisk (*) for *p*-values less than 0.05, double asterisks (**) for *p*-values less than 0.01, and triple asterisks (***) for *p*-values less than 0.001. SLG, solvent-treated larvae group; SAG, solvent-treated adult group; LLG, LCY-treated larvae group; LAG, LCY-treated adult group. Figure S5: Top 10 molecular function terms of Gene Ontology (GO) functional analysis. (A) SAG vs. SLG and (B) LAG vs. LLG. Each bar represents the intersection size, with significance levels denoted by asterisks: a single asterisk (*) for *p*-values less than 0.05, double asterisks (**) for *p*-values less than 0.01, and triple asterisks (***) for *p*-values less than 0.001. SLG, solvent-treated larvae group; SAG, solvent-treated adult group; LLG, LCY-treated larvae group; LAG, LCY-treated adult group. Figure S6: Top 10 biological processes terms of Gene Ontology (GO) functional analysis. (A) SAG vs. SLG and (B) LAG vs. LLG. Each bar represents the intersection size, with significance levels denoted by asterisks: a single asterisk (*) for *p*-values less than 0.05, double asterisks (**) for *p*-values less than 0.01, and triple asterisks (***) for *p*-values less than 0.001. SLG, solvent-treated larvae group; SAG, solvent-treated adult group; LLG, LCY-treated larvae group; LAG, LCY-treated adult group. Figure S7: Comparative transcriptomic analysis of honey bee biological responses to lambda-cyhalothrin in larvae and adults. The heatmap shows the enrichment of various biological pathways across four treatment comparisons: SAG vs. SLG, LAG vs. LLG, LLG vs. SLG, and LAG vs. SAG. Pathways are categorized into different biological processes, indicated by different colors: Metabolism (red), Genetic Information Processing (orange), Environmental Information Processing (green), Cellular Processing (blue), Organismal Systems (purple), and Human Diseases (pink). The intensity of the blue color in each cell represents the significance level (*p*-value) of the enrichment test, with darker shades indicating more significant enrichment (*p* ≤ 0.001) and lighter shades indicating less significant enrichment (*p* > 0.05). SLG, solvent-treated larvae group; SAG, solvent-treated adult group; LLG, LCY-treated larvae group; LAG, LCY-treated adult group. Figure S8: Top 10 KEGG pathways in different transcriptome comparisons. (A) SAG vs. SLG and (B) LAG vs. LLG. SLG, solvent-treated larvae group; SAG, solvent-treated adult group; LLG, LCY-treated larvae group; LAG, LCY-treated adult group. * *p* < 0.05, ** *p* < 0.01, *** *p* < 0.001. Figure S9: Correlation between transcriptome and qRT-PCR fold changes. A scatter plot showing the relationship between gene expression fold changes obtained from RNA-Seq (*x*-axis) and qRT-PCR (*y*-axis) analyses. The dotted line represents the linear regression fit (R^2^ = 0.6576). Table S1: Oligo primers used for the validation of transcriptome data. Table S2: List of DEGs for GO_MF category in LLG compared to SLG. Table S3: List of DEGs for GO_MF category in LAG compared to SAG. Table S4: List of DEGs for GO_CC category in LAG compared to LLG. Table S5: List of DEGs for GO_CC category in SAG compared to SLG. Table S6: List of DEGs for GO_MF category in LAG compared to LLG. Table S7: List of DEGs for GO_MF category in SAG compared to SLG. Table S8: List of DEGs for GO_BP category in LAG compared to LLG. Table S9: List of DEGs for GO_BP category in SAG compared to SLG. Table S10: List of significant DEGs for each enriched KEGG pathway in LLG compared to SLG. Table S11: List of significant DEGs for each enriched KEGG pathway in LAG compared to SAG. Table S12: List of significant DEGs for each enriched KEGG pathway in SAG compared to SLG. Table S13: List of significant DEGs for each enriched KEGG pathway in LAG compared to LLG. Table S14: Summary of qRT-PCR validation results for selected differentially expressed genes across treatment comparisons. File S1: Comprehensive list of differentially expressed genes (DEGs) identified across all comparisons of honey bee samples at a fold change (FC) ≥ 1.5 and raw *p*-value < 0.05, including SAG vs. SLG, LAG vs. LLG, LLG vs. SLG, and LAG vs. SAG; File S2: Detailed list of shared and common condition-specific DEGs following LCY exposure.

## Abbreviations

The following abbreviations are used in this manuscript:

(NAD(P)H)	Nicotinamide Adenine Dinucleotide Phosphate
a.i.	Active Ingredient
acyl-CoA DeLCA	Acyl-Coenzyme A Dehydrogenase, Long Chain
akhr	Adipokine tic Hormone Receptor
ATP	Adenosine Triphosphate
BP	Biological Processes
CC	Cellular Components
cDNA	Complementary DNA
cpr	Cytochrome P450 Reductase
cyp	Cytochrome P450
Cyp314a1	Cytochrome P450 314a1
cyp314A1	Cytochrome P450 314A1
cyp6a14	Cytochrome P450 6a14
cyp6k1	Cytochrome P450 6k1
cyp9e2	Cytochrome P450 9e2
DEGs	Differentially Expressed Genes
DNA	Deoxyribonucleic Acid
dop1	Dopamine Receptor 1
ELISA	Enzyme Linked Immunosorbent Assay
FAD	Flavin Adenine Dinucleotide
FC	Fold Change
FMN	Flavin Mononucleotide
FPKM	Fragments Per Kilobase of transcript per Million mapped reads
gapdh	Glyceraldehyde-3-Phosphate Dehydrogenase
GC	Guanine and Cytosine
GO	Gene Ontology
HISAT	Hierarchical Indexing Spliced Alignment of Transcripts
hsp70	Heat Shock Protein 70
K5A	Kinesin Family Member 5A
KEGG	Kyoto Encyclopedia of Genes and Genomes
KIF9	Kinesin Family Member 9
KIN-7L	Kinesin-like protein KIN-7L
LAG	LCY-treated Adult Group
LCY	Lambda-cyhalothrin
LD50	Median Lethal Dose
LLG	LCY-treated Larvae Group
LOC100578129	Kinesin-like protein KIN-7I, transcript variant X2
LOC100578936	Methylenetetrahydrofolate reductase, transcript variant X1
LOC408452	Cytochrome P450 9e2
LOC408559	Retinal dehydrogenase 1
LOC408650	Inositol oxygenase
LOC410507	Sodium-independent sulfate anion transporter, transcript variant X1
LOC411140	Putative aldehyde dehydrogenase family 7 member A1 homolog
LOC411672	Neuropeptides capa receptor-like protein
LOC413816	Sodium-independent sulfate anion transporter, transcript variant X2
LOC551044	Glucose dehydrogenase [FAD, quinone], transcript variant X2
LOC551109	Kinesin 5A, transcript variant X1
LOC551179	Methyl farnesoate epoxidase
LOC551465	Homogentisate 1,2-dioxygenase
LOC551837	Long-chain-fatty-acid–CoA ligase ACSBG2, transcript variant X1
LOC727166	Acyl-CoA Delta(11) desaturase, transcript variant X1
map2	Microtubule Associated Protein 2
MDA	Malondialdehyde
MF	Molecular Functions
nAChR	Nicotinic Acetylcholine Receptor
nAChRa9	Nicotinic Acetylcholine Receptor Alpha 9
NCBI	National Center for Biotechnology Information
NLRP	Neuroactive Ligand–Receptor Interaction Pathway
nos	Nitric Oxide Synthase
NPC	Niemann-Pick C
Q20	Quality Score of 20
Q30	Quality Score of 30
qRT-PCR	Quantitative Real-time Polymerase Chain Reaction
RIN	RNA Integrity Number
Rpl13a	Ribosomal Protein L13a
rps5	Ribosomal Protein S5
SAG	Solvent-treated Adult Group
sifr	SIFamide Receptor
SLG	Solvent-treated Larvae Group
Sog	Short Gastrulation
TOX	Thymocyte Selection Associated High Mobility Group Box Protein
tpi	Triosephosphate Isomerase
TPM	Transcript Per Million
UDP	Uridine Diphosphate
