# Supplementary material for: Chronic Larval Exposure to Lambda-Cyhalothrin Alters Gene Expression in Both Larval and Adult Honey Bees (Apis mellifera)
Source: Insects. 2025 Aug 12;16(8):833. doi: 10.3390/insects16080833 (PMC12387069; doi:10.3390/insects16080833)
Supplement: Supplementary file 1 [file insects-16-00833-s001.zip › Supplimentory file.pdf]

Chronic Larval Exposure to Lambda-Cyhalothrin Alters Gene Expression in Both Larval and Adult Honey Bees (*Apis mellifera*)

Bala Murali Krishna Vasamsetti, Kyongmi Chon*, Juyeong Kim, Minju Choi, Bo-Seon Kim,

Chang-Young Yoon, Sojeong Hwang and Kyeong-Hun Park

Detailed Methods

Age-matched larvae selection:

Honeybee (*Apis mellifera*) larvae were obtained from managed colonies at the National Institute of Agricultural Sciences, RDA, Korea. The queen was confined to an empty comb for 24 hours to induce egg-laying. After 72 hours, the combs were carefully transferred to the laboratory for the collection of first-instar larvae for *in vitro* rearing.

Grafting procedure:

First-instar larvae were carefully grafted into sterile 48-well tissue culture plates (SPL, Korea) containing grafting cell cups (Nicotplast). A fine grafting tool was used to transfer the larvae from brood combs into the wells. Plates were immediately placed inside an incubator set at 35°C and 95% ± 5% relative humidity (RH).

Larval rearing and feeding regimen:

The larval diet consisted of a mixture of royal jelly, glucose, fructose, yeast extract, and distilled water following standardized protocols as shown below

Compositions of diets for honey bee larvae feeding

Diet Name	Composition
Diet A	50% royal jelly, 6% D-glucose, 6% D-fructose, 37% distilled water, and 1% yeast extract
Diet B	50% royal jelly, 7.5% D-glucose, 7.5% D-fructose, 33.5% distilled water, and 1.5% yeast extract
Diet C	50% royal jelly, 9% D-glucose, 9% D-fructose, 30% distilled water, and 2.0% yeast extract

The diets were prepared and stored at 4°C, then brought to 35°C before feeding to ensure consistency in the volume.

Feeding schedule:

- Day 1: 20 µL of Diet A
- Day 2: No feeding
- Day 3: 20 µL of Diet B
- Day 4: 30 µL of Diet C
- Day 5: 40 µL of Diet C
- Day 6: 50 µL of Diet C

LCY Chronic Exposure and Experimental Conditions:

The LCY stock was dissolved in an appropriate diet. LCY exposure commenced on Day 3 post-grafting and continued until Day 6 post-grafting. During the entire exposure period, each larva was exposed to a total of 0.004 μg. For the solvent control group, the larvae were fed a diet containing 0.5% acetone. The acetone concentration was maintained at 0.5% in the LCY exposure group.

On Day 3 post-grafting, each larva received 20 μL of Diet B containing 0.00057 μg of LCY. On Day 4 post-grafting, each larva received 30 μL of Diet C containing 0.00086 μg of LCY. On Day 5 post-grafting, each larva received 40 μL of Diet C containing 0.0011 μg of LCY, and on Day 6 post-grafting, each larva received 50 μL of Diet C containing 0.00143 μg of LCY.

The diets were carefully released using a pipette through the wall of the cup, ensuring that the larvae were not submerged in the diet. Feeding was conducted quickly in a sterile environment to avoid contamination and desiccation. Dead larvae were removed during feeding.

Larval rearing was conducted at a controlled temperature of 35°C and a humidity level of 95% ± 5% RH. Humidity was regulated using desiccators filled with a saturated solution of potassium sulfate, creating optimal conditions for larval development. On Day 8, the larvae were transferred to pupal rearing environments, maintaining the same temperature but adjusting the humidity to 80% ± 5% RH using a saturated sodium chloride solution. By Day 15, the pupae were moved to emergence boxes containing a 50% sucrose solution, which served as feed and allowed the newly emerged bees to move freely.

Quantitative Reverse Transcription Polymerase Chain Reaction (qRT-PCR) Methodology

For the qRT-PCR process, RNA extraction was carried out in triplicates for every experimental condition. Initially, samples were defrosted and maintained on ice. Using fine scissors, samples were finely chopped and homogenized with a Kimble® Pellet Pestle® Cordless Motor (DWK Life Sciences, Millville, NJ, USA) along with a matching pestle (SL.Tub3101.1, Scilab, Daejon, Chungcheongnam-do, Republic of Korea). Approximately 50 mg of either larval or adult tissue homogenate was transferred into a 1.5 mL Eppendorf tube (Eppendorf, Hamburg, Germany) and lysed with 500 μL of Trizol-plus solution (Progen Life Sciences, New York, NY, USA; #PG1117). This mixture was then centrifuged at 8000× g for 3 minutes at 4°C. The clear supernatant was moved to a column in the Direct-zol RNA Miniprep Kit (R2052, Zymo Research, Irvine, CA, USA), and RNA extraction proceeded as per the kit's instructions. RNA quality was checked using a Nanodrop 2000 spectrophotometer (Thermo Fisher Scientific, Waltham, MA, USA).

For cDNA synthesis, 1500 ng of RNA per reaction was used with the AccuPower® RocketScript™ Cycle RT PreMix (K-2205, Bioneer, Daejeon, Chungcheongnam-do, Republic of

Korea). Since the Direct-zol RNA Miniprep Kit includes a DNase treatment step, no further DNase application was necessary.

Quantitative PCR was performed using AccuPower® 2X GreenStar™ qPCR Master Mix (K6251, Bioneer, Daejeon, Chungcheongnam-do, Republic of Korea) on a CFX96 Dx real-time PCR detection system (Bio-Rad, Hercules, CA, USA), following the supplier's protocol. Each PCR reaction contained 50 ng of cDNA in a total volume of 20 μL. The oligonucleotide primers were used at 10 picomoles per reaction, with annealing temperatures set based on the average melting temperatures of the primers (refer to Table S8). Each reaction setup included no-template controls in triplicate.

The qPCR conditions were as follows: an initial denaturation at 95°C for 5 minutes, followed by 45 cycles of 95°C for 10 seconds, annealing for 10 seconds at 60°C, and extension at 60°C for 10 seconds. A melting curve analysis from 65°C to 95°C, increasing by 0.5°C increments, confirmed the specificity of the amplification. Ct values were calculated from three biological and three technical replicates per condition. Gene expression levels were quantified using the $2^{-\Delta\Delta CT}$ method, normalizing to the average Ct values of RPL13a, GAPDH, and RPS5 as reference genes. Details of the oligonucleotide primers are provided in Table S1.

Figures

Figure S1

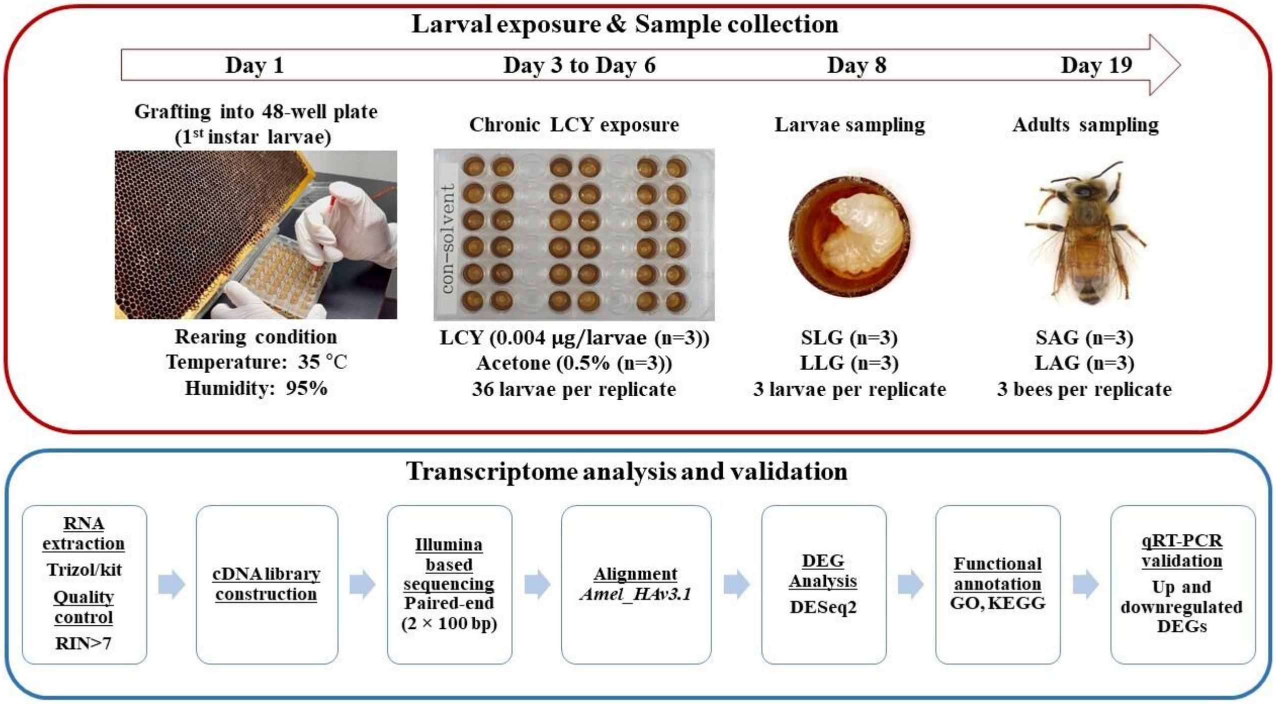

Figure S1: Schematic representation of the experimental workflow for LCY exposure and transcriptome analysis in *Apis mellifera*. First instar larvae were grafted into 48-well plates (36 larvae per replicate) and reared at 35 °C and 95% relative humidity. From Day 3 to Day 6, larvae were chronically exposed to LCY (0.004 µg/larva; $n = 3$) or acetone control (0.5%; $n = 3$). Samples were collected at the larval stage (SLG: solvent-treated larvae group; LLG: LCY-treated larvae group; Day 8; 3 larvae per replicate) and adult stage (SAG: solvent-treated adult group; LAG: LCY-treated adult group; Day 19; 3 bees per replicate). Total RNA was extracted (RIN > 7), followed by cDNA library construction, Illumina paired-end sequencing (2 × 100 bp), read alignment to the *Apis mellifera* reference genome (*Amel_HAv3.1*), differential gene expression (DEG) analysis using DESeq2, Gene Ontology (GO) and Kyoto Encyclopedia of Genes and Genomes (KEGG) annotation, and qRT-PCR validation of selected DEGs.

Figure S2A

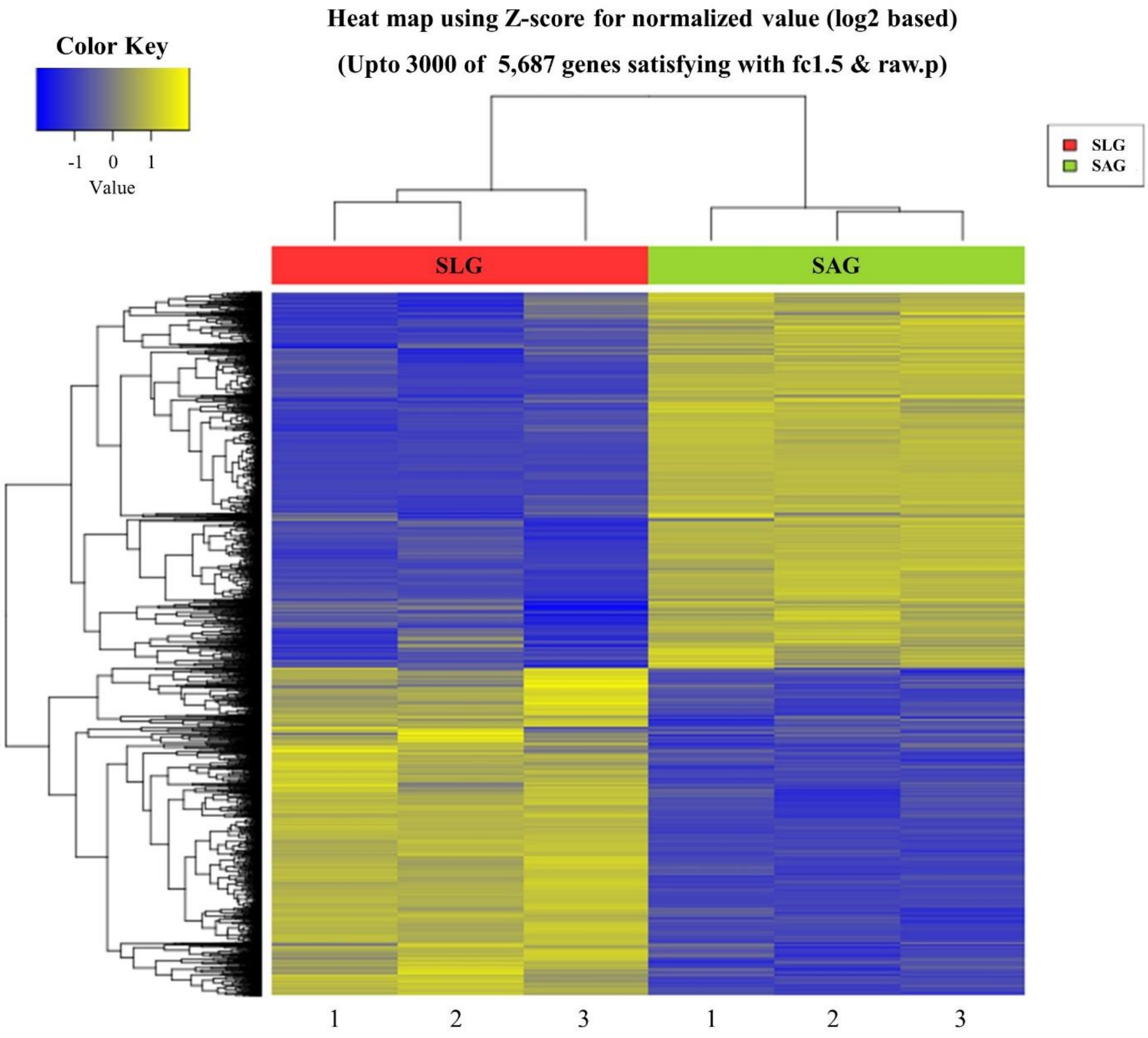
Heat map using Z-score for normalized value (log2 based)
(Upto 3000 of 5,687 genes satisfying with fc1.5 & raw.p)
Color Key
-1 0 1
Value
SLG
SAG
SLG
SAG
1 2 3 1 2 3

Figure S2B

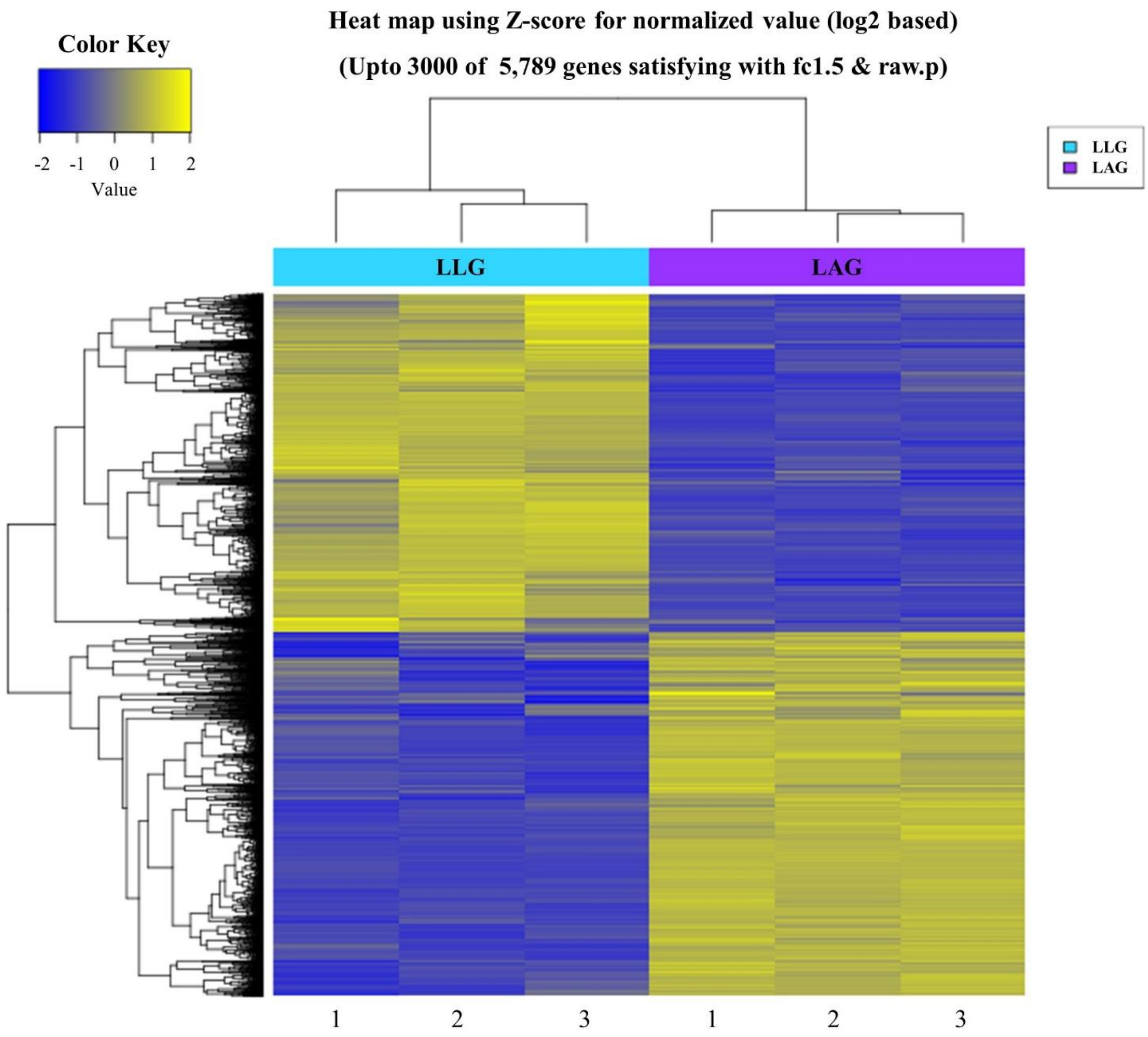
Color Key
-2 -1 0 1 2
Value
Heat map using Z-score for normalized value (log2 based)
(Upto 3000 of 5,789 genes satisfying with fc1.5 & raw.p)
LLG
LAG
LLG
LAG
1 2 3 1 2 3

Figure S2C

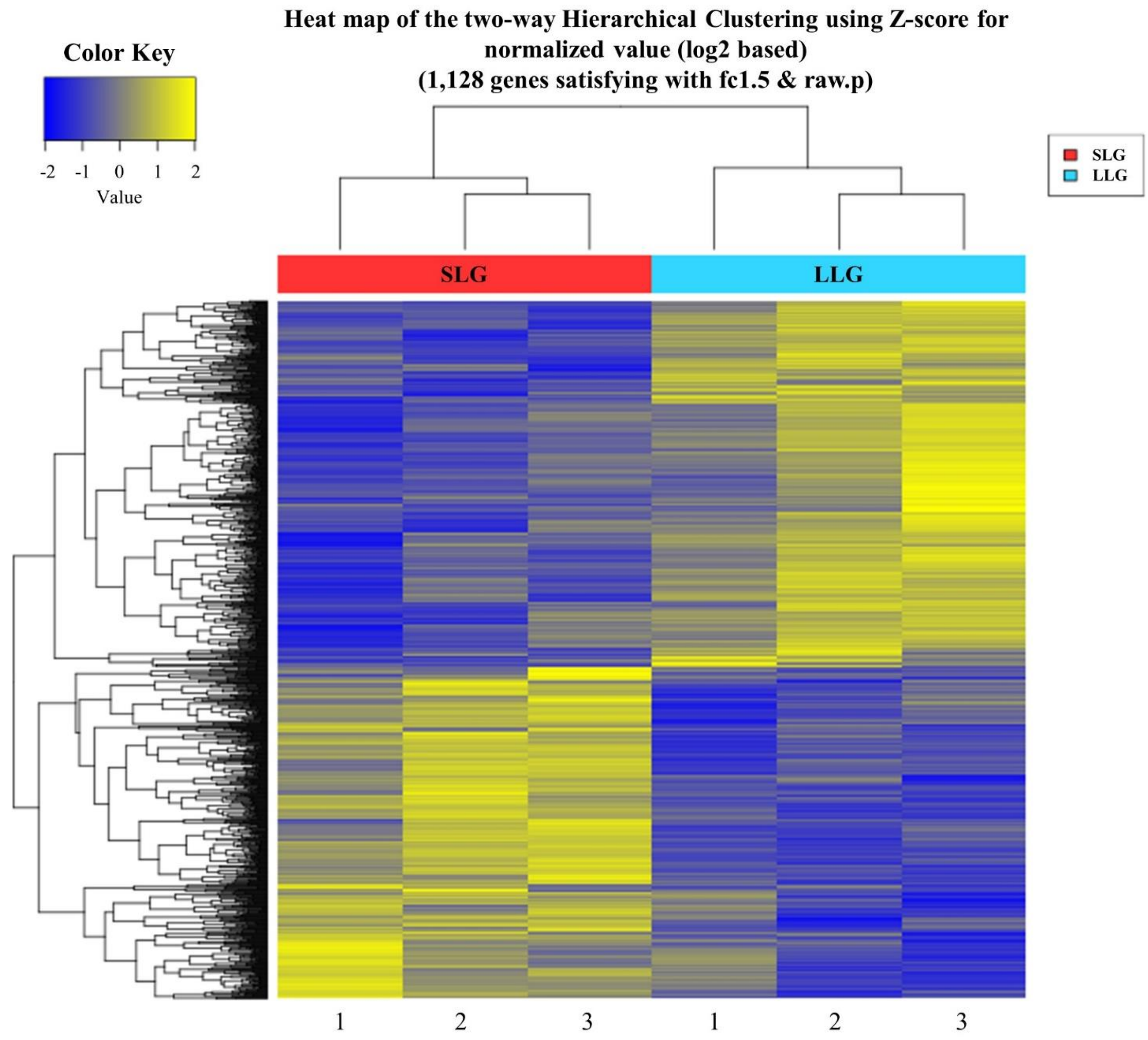
Heat map of the two-way Hierarchical Clustering using Z-score for normalized value (log2 based)
(1,128 genes satisfying with fc1.5 & raw.p)
Color Key
-2 -1 0 1 2
Value
SLG
LLG
SLG
LLG
1
2
3
1
2
3

Figure S2D

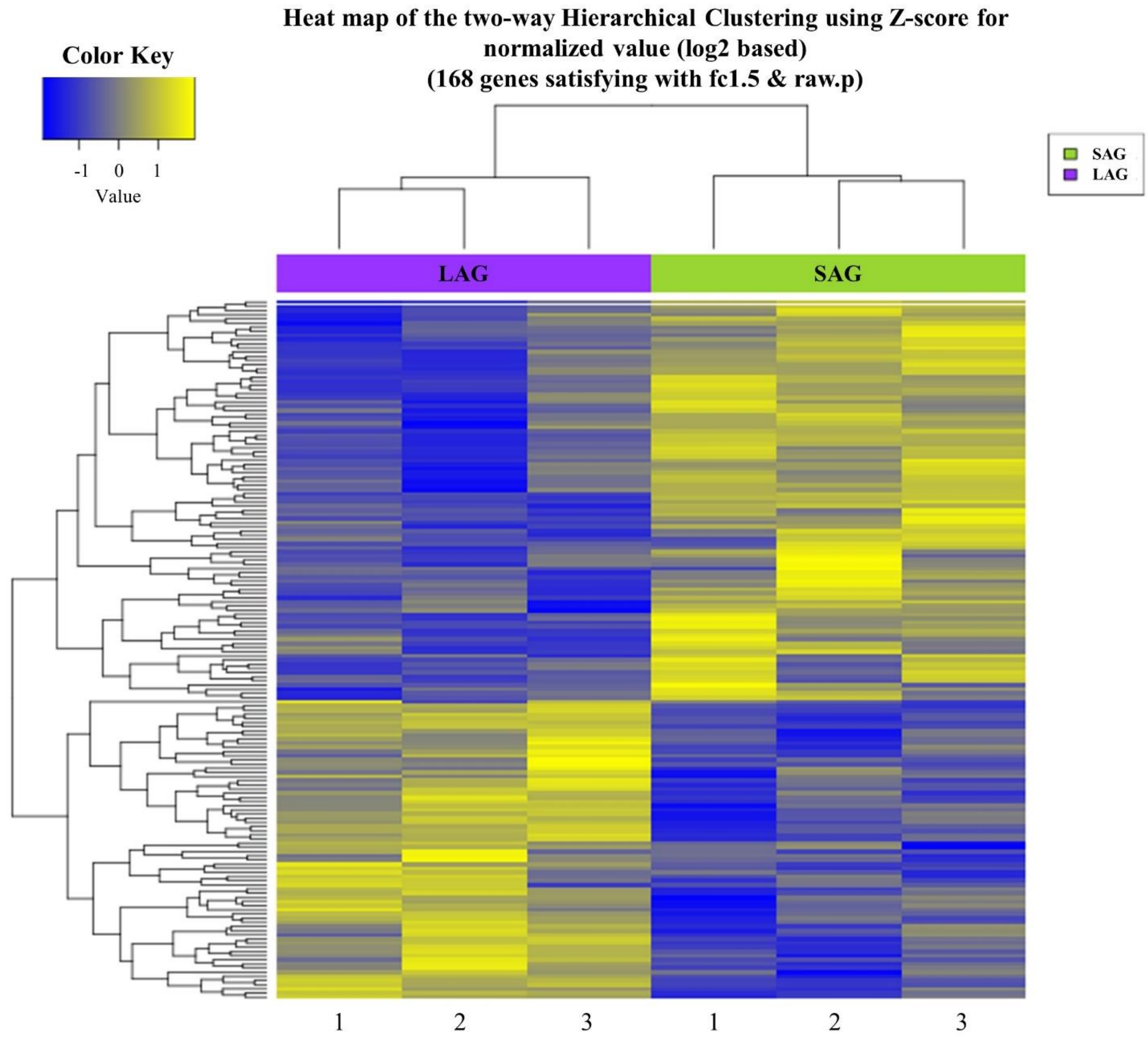

Figure S2: Comparative transcriptomic analyses of honeybee responses to lambda-cyhalothrin in honeybee larvae and adults. Heat map illustrates the differential expression of genes between four treatment comparisons: (A) SAG vs. SLG, (B) LAG vs. LLG, (C) LLG vs. SLG, and (D) LAG vs. SAG. The data are presented as z-scores of normalized log2-transformed expression values. Two-way hierarchical clustering was applied to organize the data. Gene expression differences are considered significant at a fold change of 1.5 and with significance threshold p-values applied. SLG, solvent-treated larvae group; SAG, solvent-treated adult group; LLG, LCY-treated larvae group; LAG, LCY-treated adult group.

Figure S3A

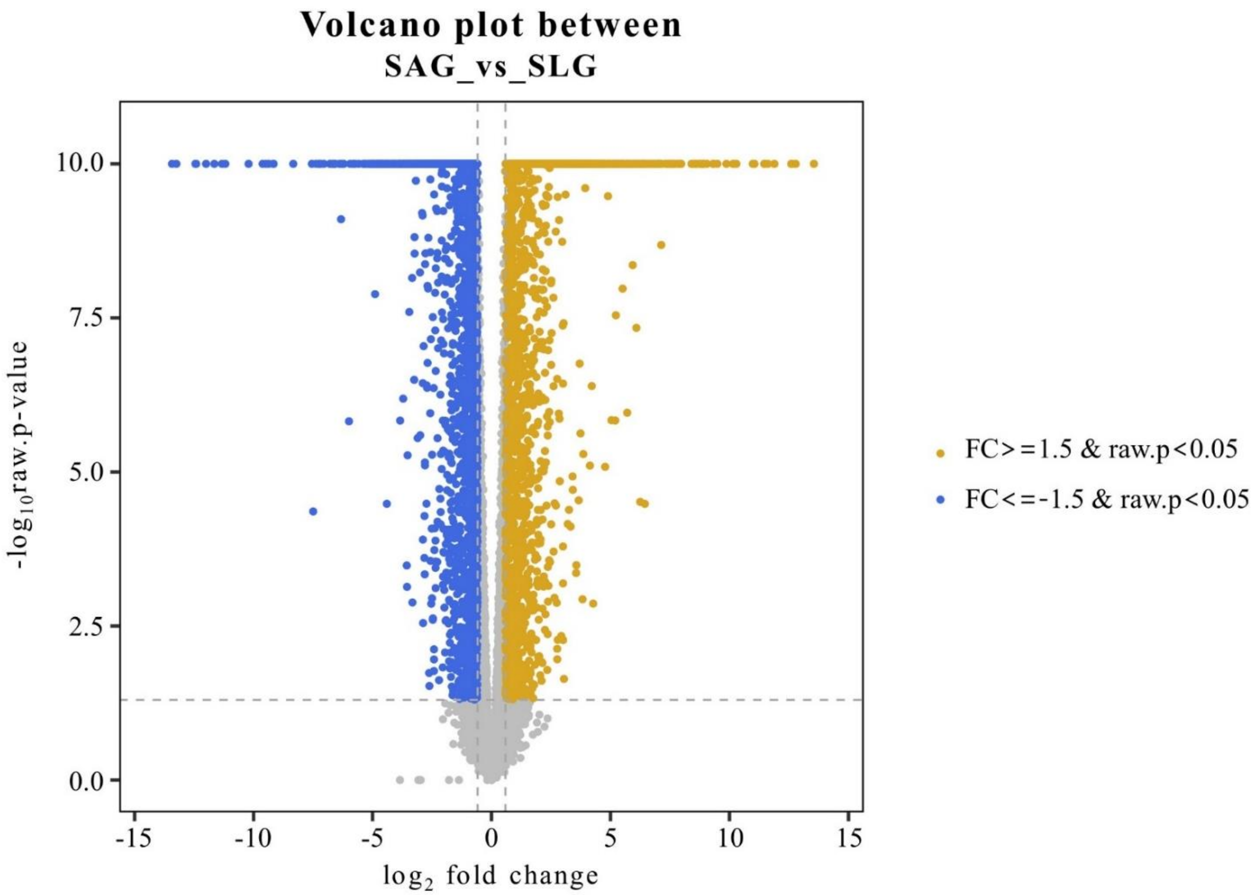

Volcano plot between
SAG_vs_SLG
10.0
7.5
5.0
2.5
0.0
-log10raw.p-value
-15
-10
-5
0
5
10
15
log2 fold change
FC>=1.5 & raw.p<0.05
FC<=-1.5 & raw.p<0.05

Figure S3B

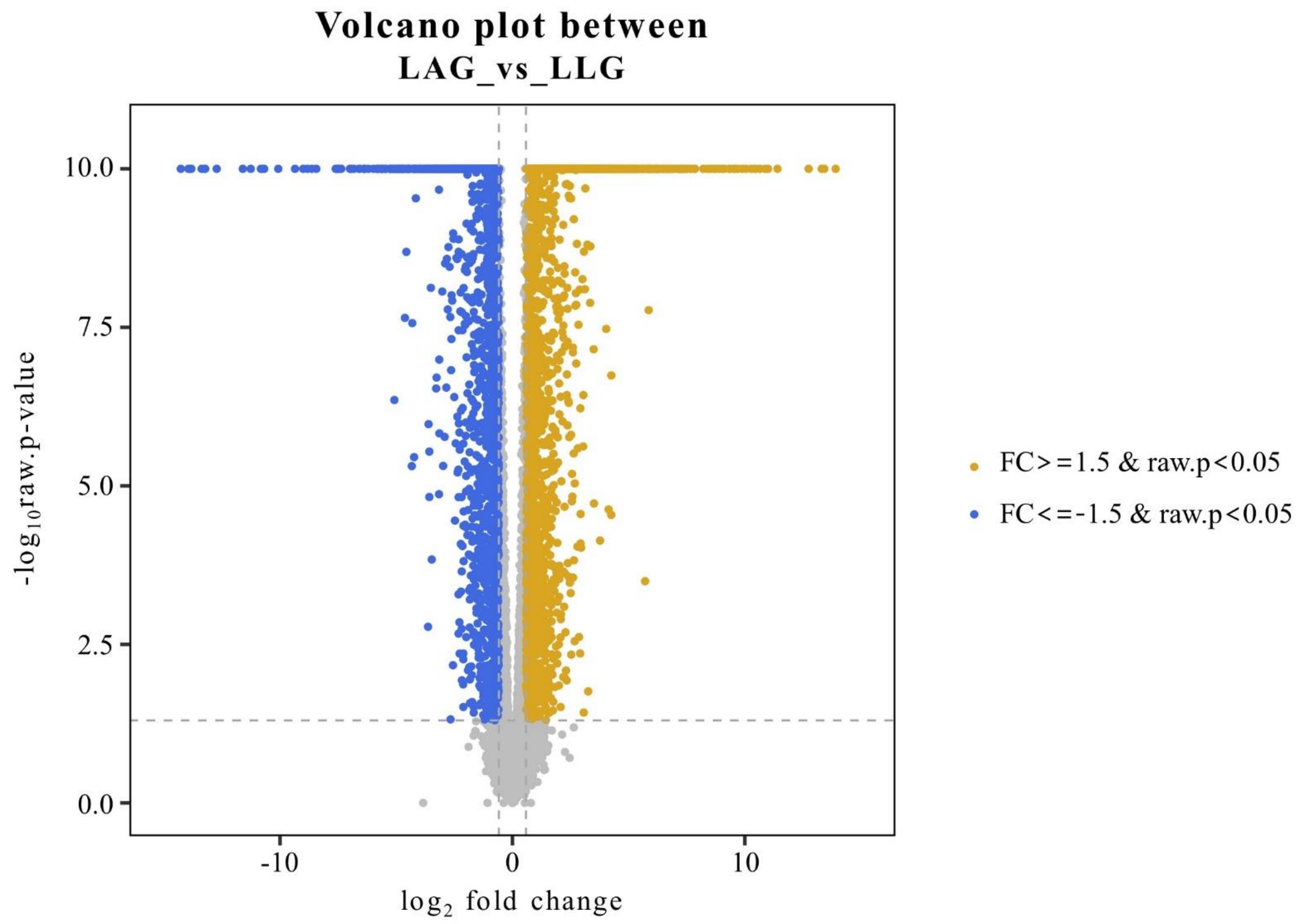

Volcano plot between
LAG_vs_LLG
10.0
7.5
5.0
2.5
0.0
-log10raw.p-value
-10
0
10
log2 fold change
FC>=1.5 & raw.p<0.05
FC<=-1.5 & raw.p<0.05

Figure S3C

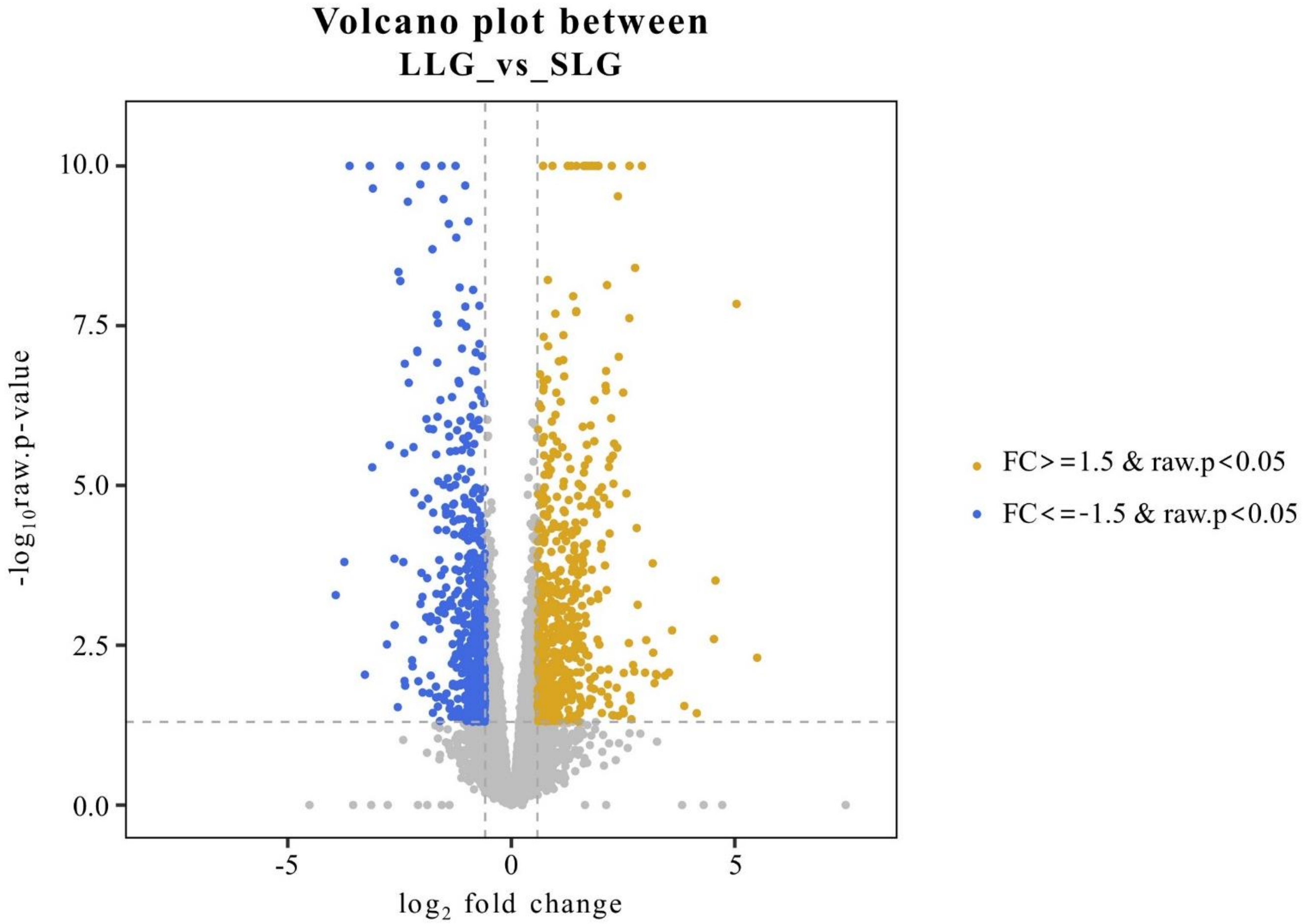

Volcano plot between
LLG_vs_SLG
10.0
7.5
5.0
2.5
0.0
-5
0
5
$-log_{10}$raw.p-value
log_2 fold change
FC>=1.5 & raw.p<0.05
FC<=-1.5 & raw.p<0.05

Figure S3D

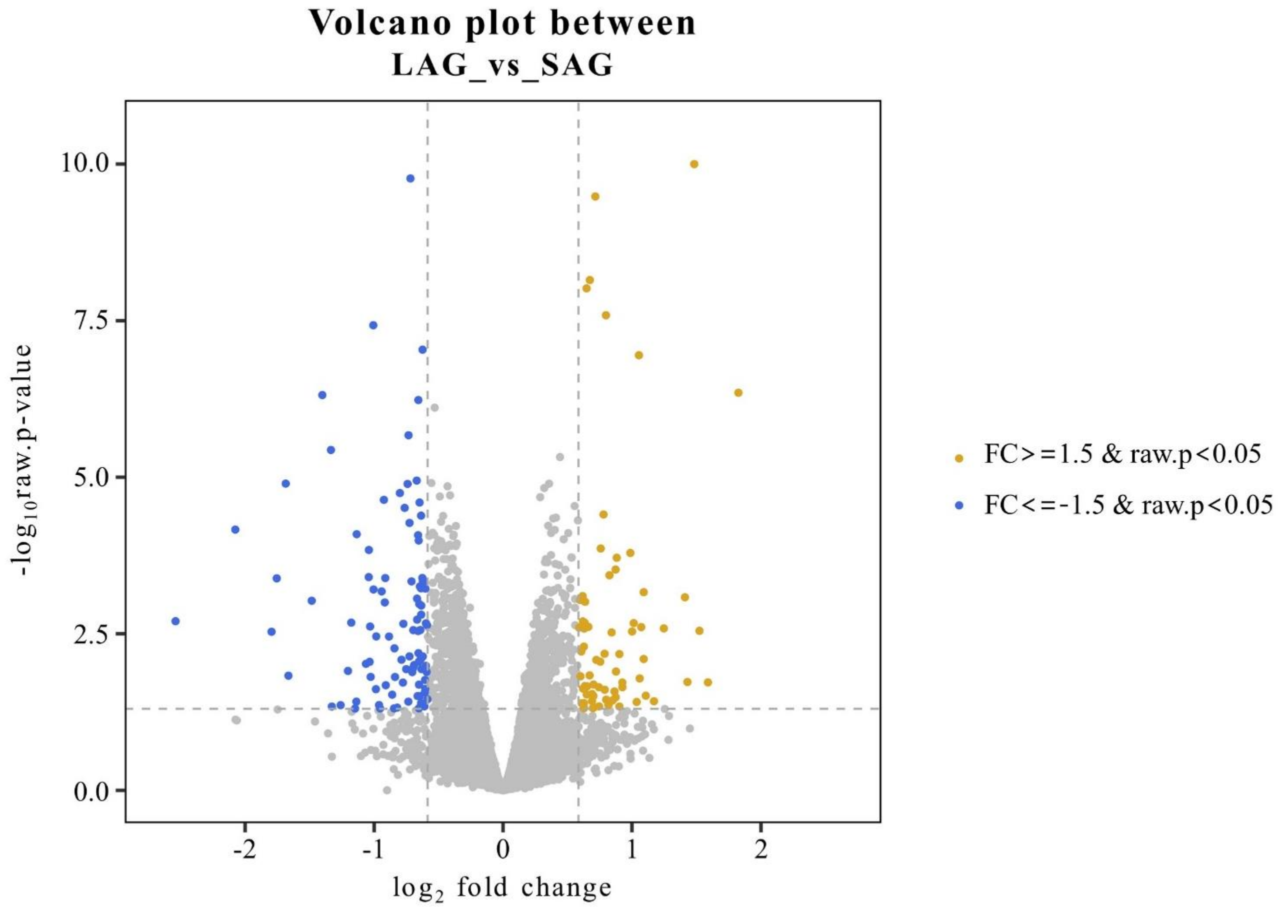

Figure S3: Comparative transcriptomic analyses of honeybee responses to lambda-cyhalothrin in honeybee larvae and adults. Volcano plots illustrate the differential expression of genes between four treatment comparisons: (A) SAG vs. SLG, (B) LAG vs. LLG, (C) LLG vs. SLG, and (D) LAG vs. SAG. The data are presented as log2 fold changes against -log10 raw p-values. Gene expression differences are considered significant at a fold change (FC) of 1.5 and a raw p-value < 0.05, with upregulated genes shown in yellow and downregulated genes in blue. SLG, solvent-treated larvae group; SAG, solvent-treated adult group; LLG, LCY-treated larvae group; LAG, LCY-treated adult group.

Figure S4

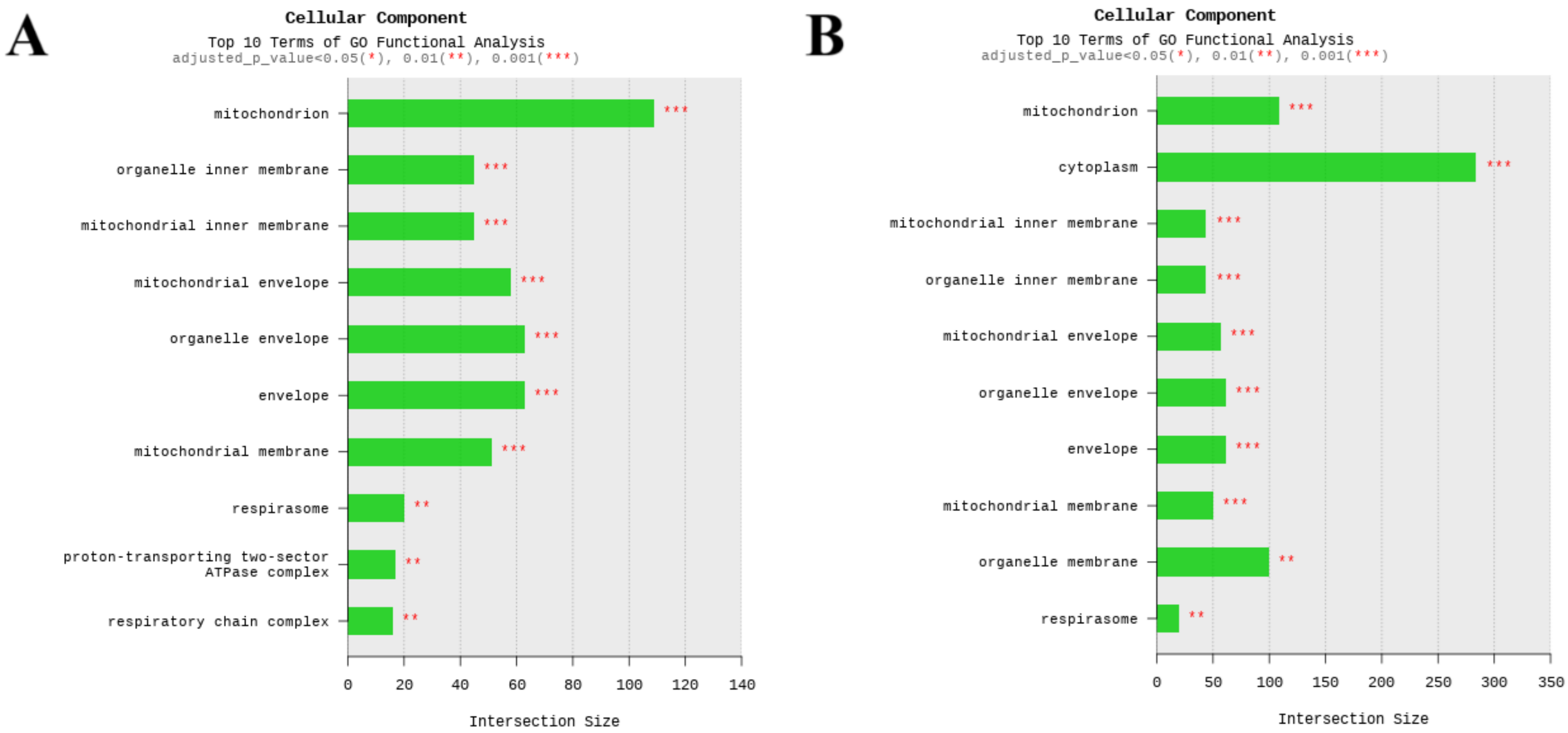

Figure S4: Top 10 cellular component terms of Gene Ontology (GO) functional analysis. (A) SAG vs SLG and (B) LAG vs LLG. Each bar represents the intersection size, with significance levels denoted by asterisks: a single asterisk (*) for p-values less than 0.05, double asterisks (**) for p-values less than 0.01, and triple asterisks (***) for p-values less than 0.001. SLG, solvent-treated larvae group; SAG, solvent-treated adult group; LLG, LCY-treated larvae group; LAG, LCY-treated adult group.

Figure S5

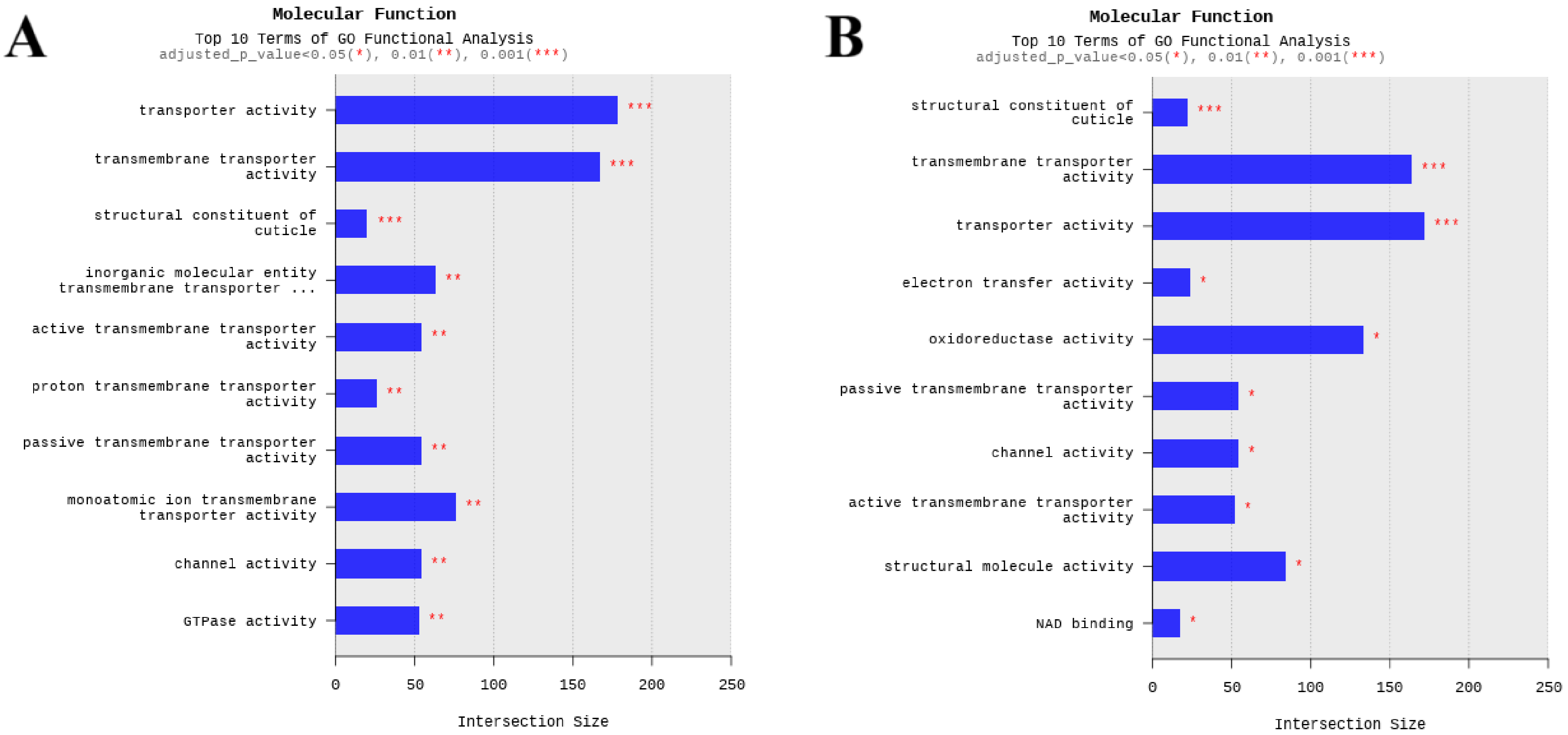

Figure S5: Top 10 molecular function terms of Gene Ontology (GO) functional analysis. (A) SAG vs SLG and (B) LAG vs LLG. Each bar represents the intersection size, with significance levels denoted by asterisks: a single asterisk (*) for p-values less than 0.05, double asterisks (**) for p-values less than 0.01, and triple asterisks (***) for p-values less than 0.001. SLG, solvent-treated larvae group; SAG, solvent-treated adult group; LLG, LCY-treated larvae group; LAG, LCY-treated adult group.

Figure S6

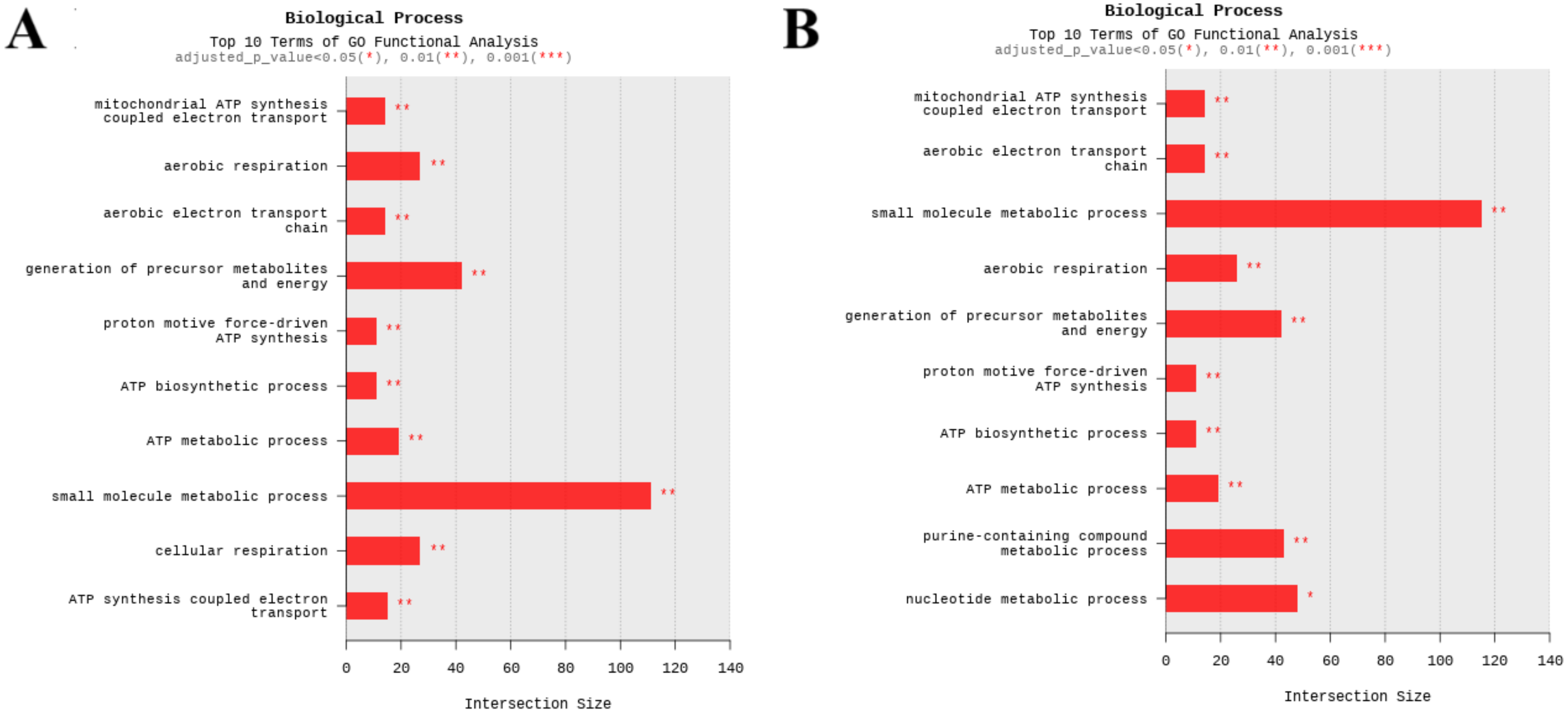

Figure S6: Top 10 biological processes terms of Gene Ontology (GO) functional analysis. (A) SAG vs SLG and (B) LAG vs LLG. Each bar represents the intersection size, with significance levels denoted by asterisks: a single asterisk (*) for p-values less than 0.05, double asterisks (**) for p-values less than 0.01, and triple asterisks (***) for p-values less than 0.001. SLG, solvent-treated larvae group; SAG, solvent-treated adult group; LLG, LCY-treated larvae group; LAG, LCY-treated adult group.

Figure S7

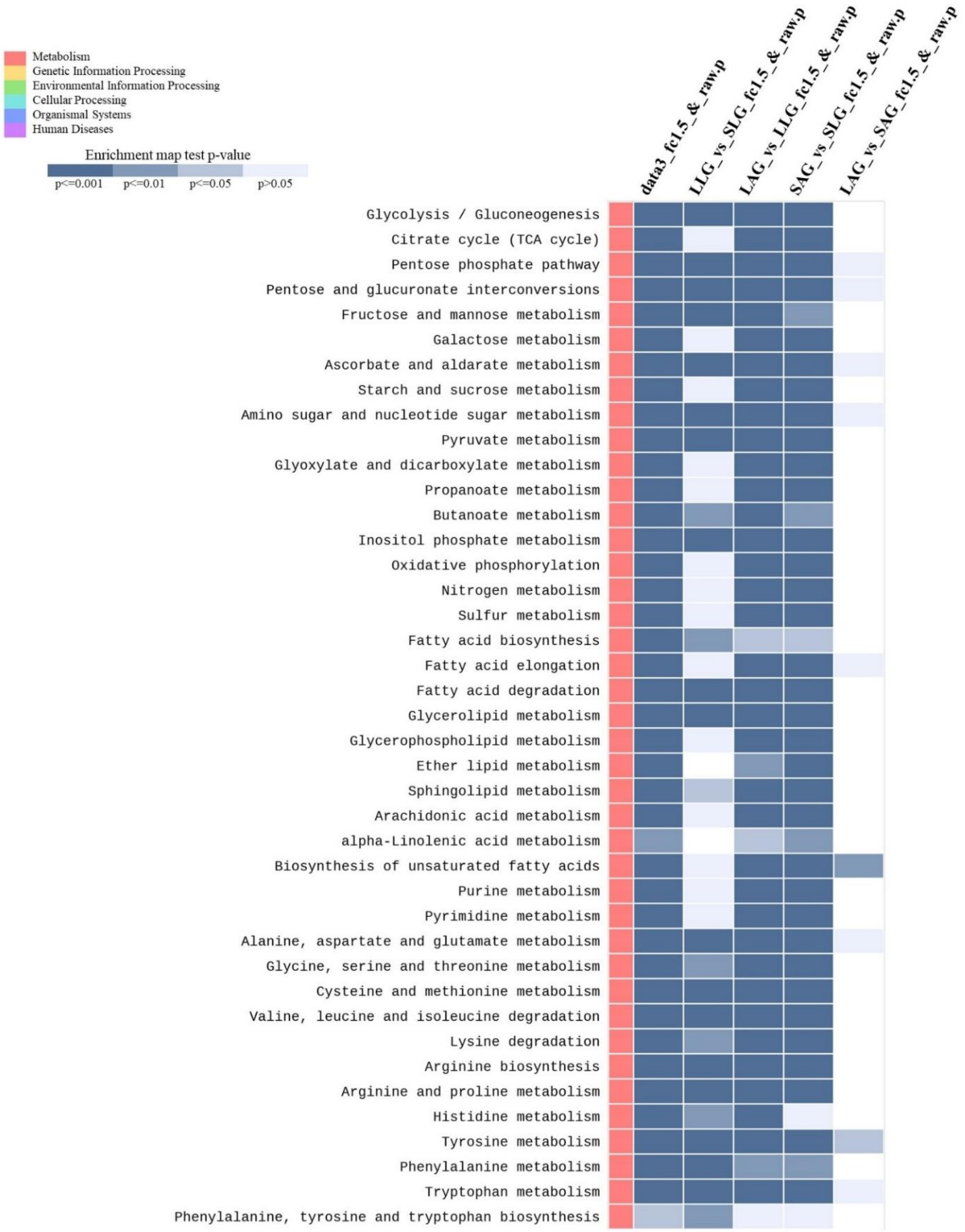

Metabolism
Genetic Information Processing
Environmental Information Processing
Cellular Processing
Organismal Systems
Human Diseases
Enrichment map test p-value
p<=0.001
p<=0.01
p<=0.05
p>0.05
data3_fc1.5_&_raw.p
LLG_vs_SLG_fc1.5_&_raw.p
LAG_vs_LLG_fc1.5_&_raw.p
SAG_vs_SLG_fc1.5_&_raw.p
LAG_vs_SAG_fc1.5_&_raw.p
Glycolysis / Gluconeogenesis
Citrate cycle (TCA cycle)
Pentose phosphate pathway
Pentose and glucuronate interconversions
Fructose and mannose metabolism
Galactose metabolism
Ascorbate and aldarate metabolism
Starch and sucrose metabolism
Amino sugar and nucleotide sugar metabolism
Pyruvate metabolism
Glyoxylate and dicarboxylate metabolism
Propanoate metabolism
Butanoate metabolism
Inositol phosphate metabolism
Oxidative phosphorylation
Nitrogen metabolism
Sulfur metabolism
Fatty acid biosynthesis
Fatty acid elongation
Fatty acid degradation
Glycerolipid metabolism
Glycerophospholipid metabolism
Ether lipid metabolism
Sphingolipid metabolism
Arachidonic acid metabolism
alpha-Linolenic acid metabolism
Biosynthesis of unsaturated fatty acids
Purine metabolism
Pyrimidine metabolism
Alanine, aspartate and glutamate metabolism
Glycine, serine and threonine metabolism
Cysteine and methionine metabolism
Valine, leucine and isoleucine degradation
Lysine degradation
Arginine biosynthesis
Arginine and proline metabolism
Histidine metabolism
Tyrosine metabolism
Phenylalanine metabolism
Tryptophan metabolism
Phenylalanine, tyrosine and tryptophan biosynthesis

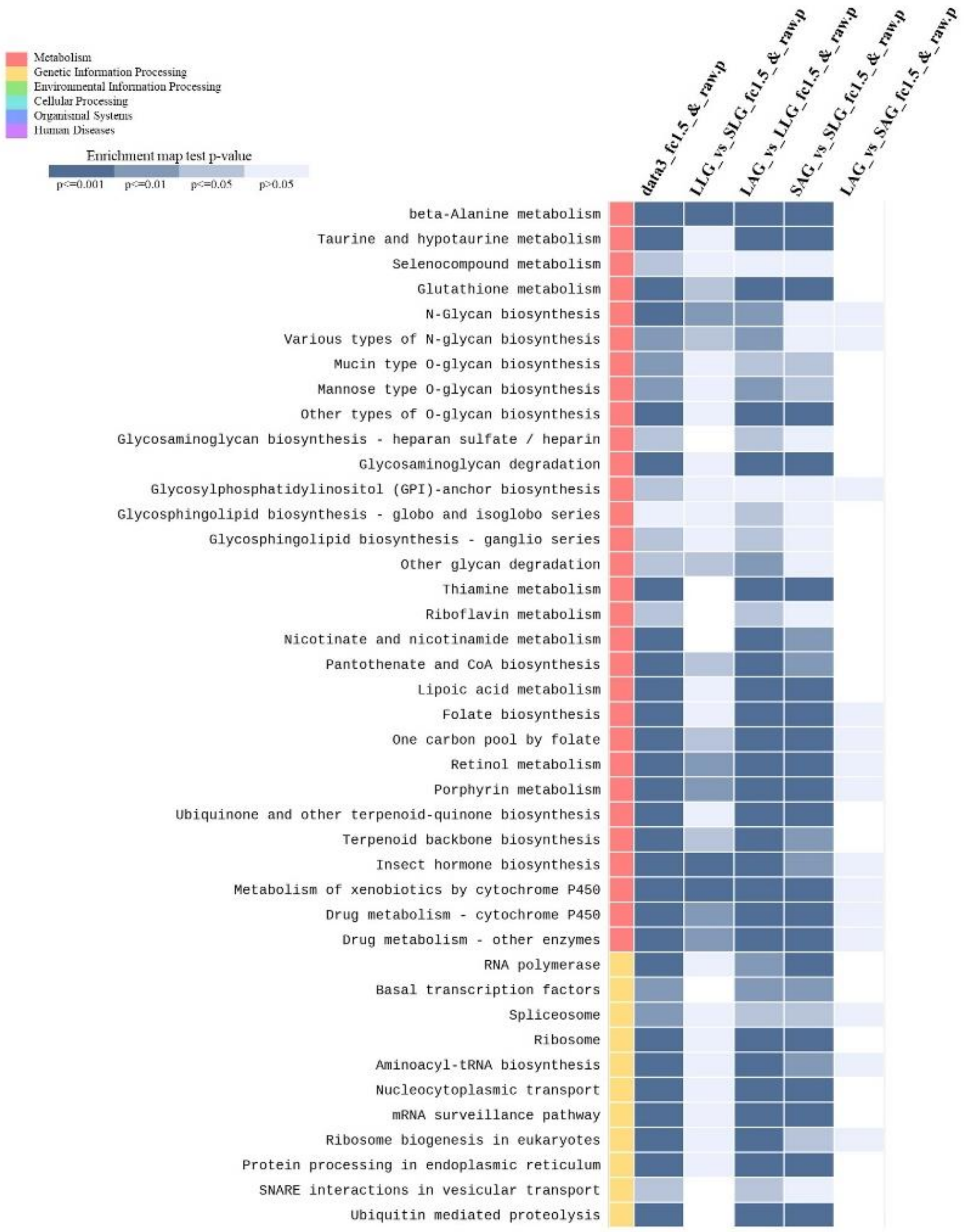

Metabolism
Genetic Information Processing
Environmental Information Processing
Cellular Processing
Organismal Systems
Human Diseases
Enrichment map test p-value
p<=0.001
p<=0.01
p<=0.05
p>0.05
data3_fc1.5_&_raw.p
LLG_vs_SLG_fc1.5_&_raw.p
LAG_vs_LLG_fc1.5_&_raw.p
SAG_vs_SLG_fc1.5_&_raw.p
LAG_vs_SAG_fc1.5_&_raw.p
beta-Alanine metabolism
Taurine and hypotaurine metabolism
Selenocompound metabolism
Glutathione metabolism
N-Glycan biosynthesis
Various types of N-glycan biosynthesis
Mucin type O-glycan biosynthesis
Mannose type O-glycan biosynthesis
Other types of O-glycan biosynthesis
Glycosaminoglycan biosynthesis - heparan sulfate / heparin
Glycosaminoglycan degradation
Glycosylphosphatidylinositol (GPI)-anchor biosynthesis
Glycosphingolipid biosynthesis - globo and isoglobo series
Glycosphingolipid biosynthesis - ganglio series
Other glycan degradation
Thiamine metabolism
Riboflavin metabolism
Nicotinate and nicotinamide metabolism
Pantothenate and CoA biosynthesis
Lipoic acid metabolism
Folate biosynthesis
One carbon pool by folate
Retinol metabolism
Porphyrin metabolism
Ubiquinone and other terpenoid-quinone biosynthesis
Terpenoid backbone biosynthesis
Insect hormone biosynthesis
Metabolism of xenobiotics by cytochrome P450
Drug metabolism - cytochrome P450
Drug metabolism - other enzymes
RNA polymerase
Basal transcription factors
Spliceosome
Ribosome
Aminoacyl-tRNA biosynthesis
Nucleocytoplasmic transport
mRNA surveillance pathway
Ribosome biogenesis in eukaryotes
Protein processing in endoplasmic reticulum
SNARE interactions in vesicular transport
Ubiquitin mediated proteolysis

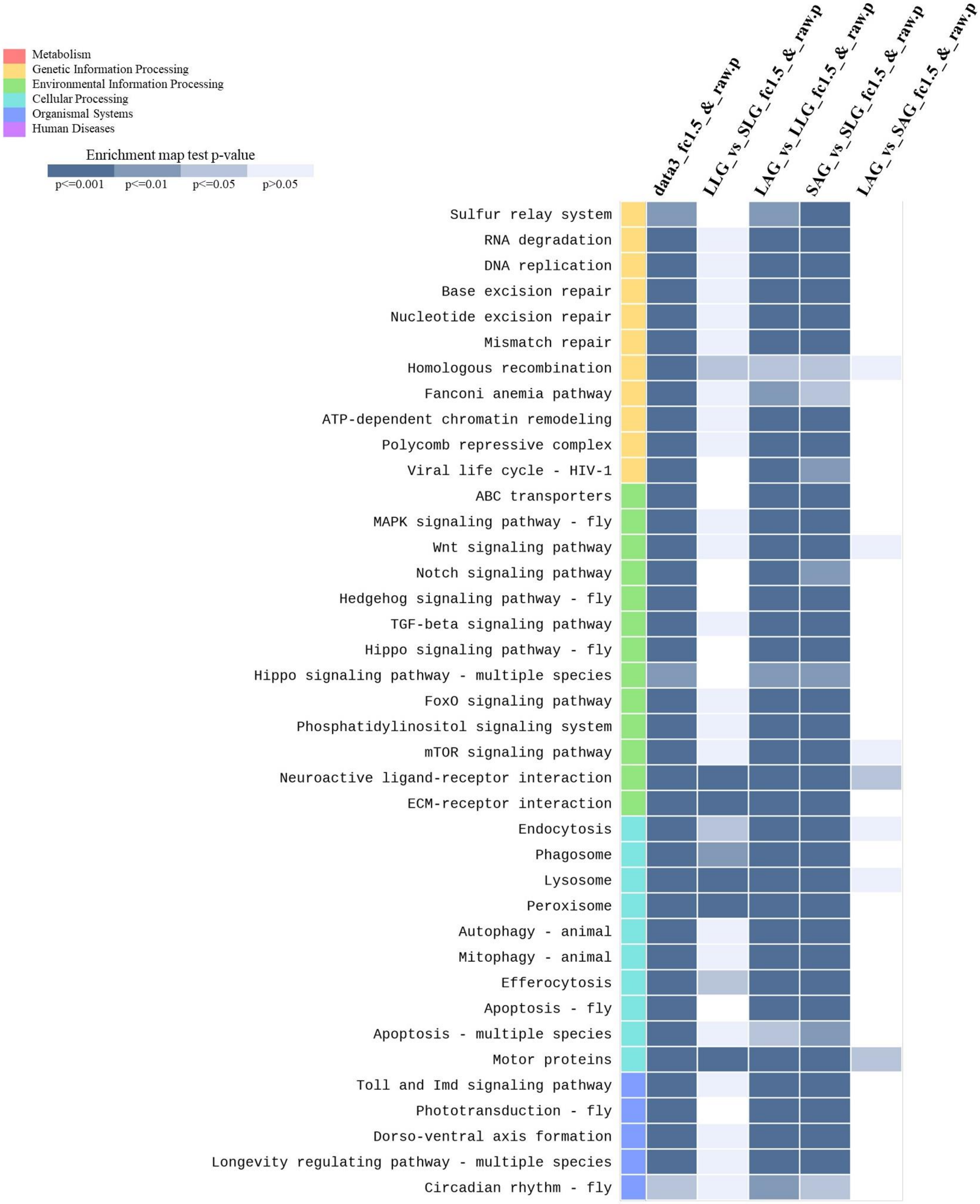

Metabolism
Genetic Information Processing
Environmental Information Processing
Cellular Processing
Organismal Systems
Human Diseases
Enrichment map test p-value
p<=0.001
p<=0.01
p<=0.05
p>0.05
data3_fc1.5_&_raw.p
LLG_vs_SLG_fc1.5_&_raw.p
LAG_vs_LLG_fc1.5_&_raw.p
SAG_vs_SLG_fc1.5_&_raw.p
LAG_vs_SAG_fc1.5_&_raw.p
Sulfur relay system
RNA degradation
DNA replication
Base excision repair
Nucleotide excision repair
Mismatch repair
Homologous recombination
Fanconi anemia pathway
ATP-dependent chromatin remodeling
Polycomb repressive complex
Viral life cycle - HIV-1
ABC transporters
MAPK signaling pathway - fly
Wnt signaling pathway
Notch signaling pathway
Hedgehog signaling pathway - fly
TGF-beta signaling pathway
Hippo signaling pathway - fly
Hippo signaling pathway - multiple species
FoxO signaling pathway
Phosphatidylinositol signaling system
mTOR signaling pathway
Neuroactive ligand-receptor interaction
ECM-receptor interaction
Endocytosis
Phagosome
Lysosome
Peroxisome
Autophagy - animal
Mitophagy - animal
Efferocytosis
Apoptosis - fly
Apoptosis - multiple species
Motor proteins
Toll and Imd signaling pathway
Phototransduction - fly
Dorso-ventral axis formation
Longevity regulating pathway - multiple species
Circadian rhythm - fly

Figure S7: Comparative transcriptomic analysis of honeybee biological responses to lambda-cyhalothrin in larvae and adults. The heatmap shows the enrichment of various biological pathways across four treatment comparisons: SAG vs. SLG, LAG vs. LLG, LLG vs. SLG, and LAG vs. SAG. Pathways are categorized into different biological processes, indicated by different colors: Metabolism (red), Genetic Information Processing (orange), Environmental Information Processing (green), Cellular Processing (blue), Organismal Systems (purple), and Human Diseases (pink). The intensity of the blue color in each cell represents the significance level (p-value) of the enrichment test, with darker shades indicating more significant enrichment ($p \leq 0.001$) and lighter shades indicating less significant enrichment ($p > 0.05$). SLG, solvent-treated larvae group; SAG, solvent-treated adult group; LLG, LCY-treated larvae group; LAG, LCY-treated adult group.

Figure S8

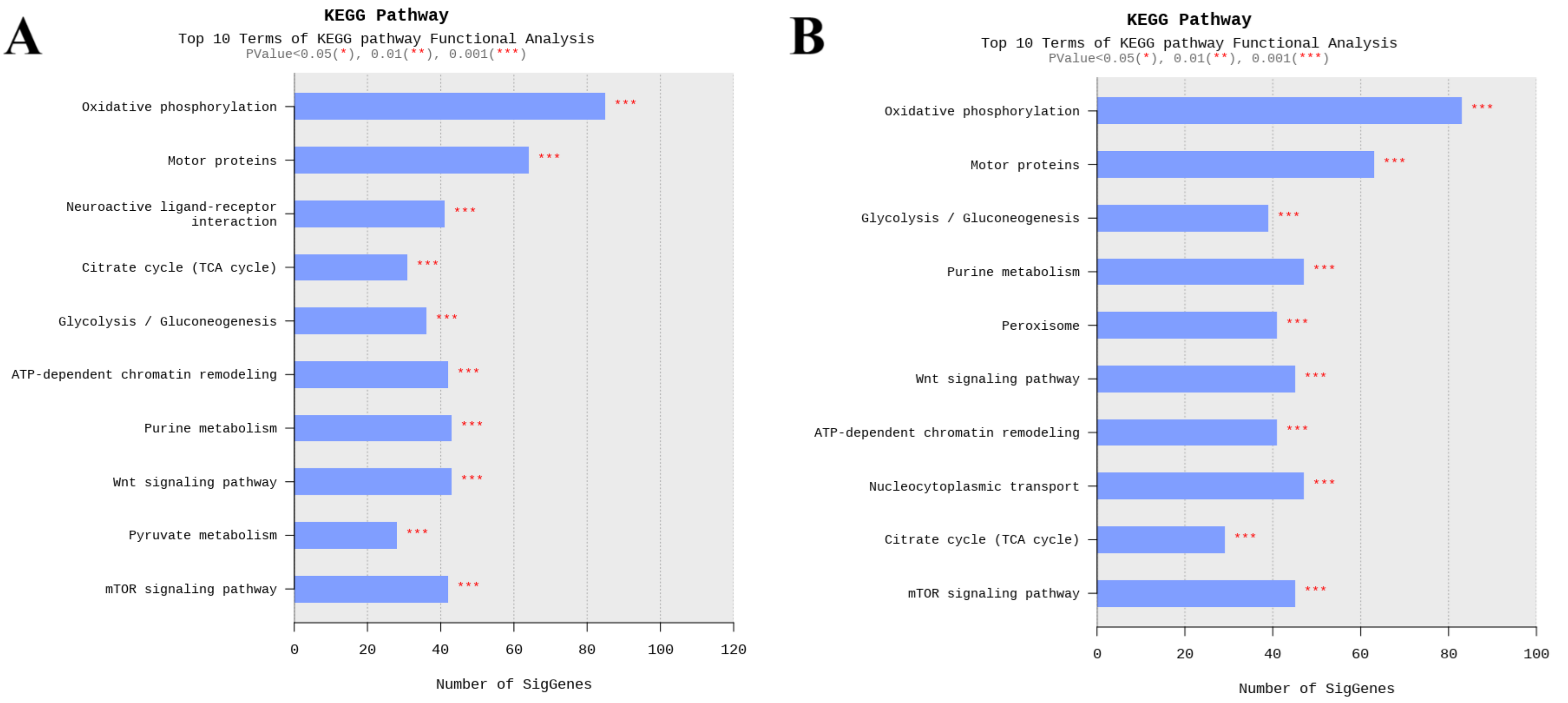

Figure S8. Top 10 KEGG pathways in different transcriptome comparisons. (A) SAG vs SLG and (B) LAG vs LLG. SLG, solvent-treated larvae group; SAG, solvent-treated adult group; LLG, LCY-treated larvae group; LAG, LCY-treated adult groups. * $p < 0.05$, ** $p < 0.01$, *** $p < 0.001$.

Figure S9

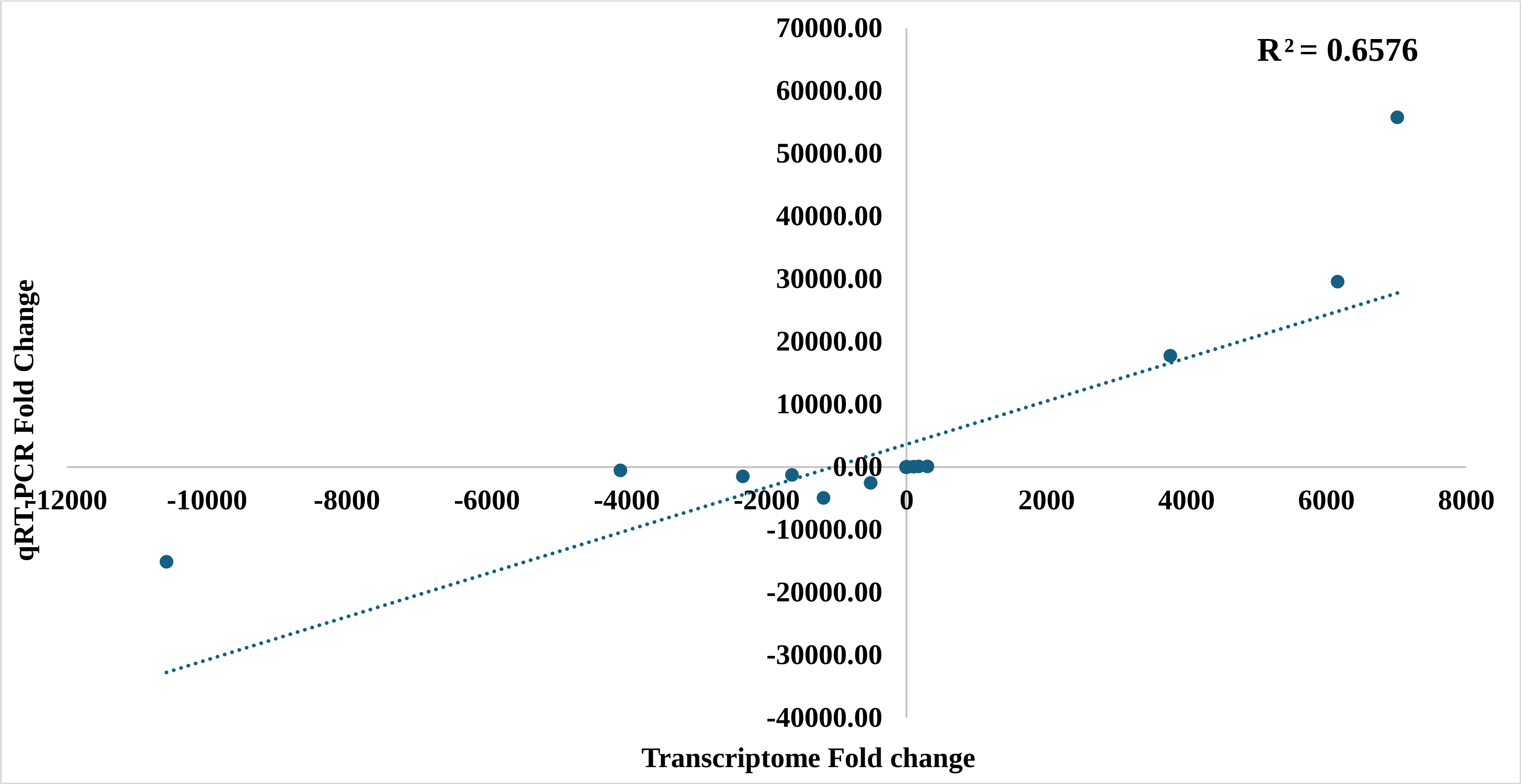

Figure S9. Correlation between transcriptome and qRT-PCR fold changes. A scatter plot showing the relationship between gene expression fold changes obtained from RNA-Seq (x-axis) and qRT-PCR (y-axis) analyses. The dotted line represents the linear regression fit ($R^2 = 0.6576$).

Tables

Table S1. List of Oligo Primers used in the qRTPCR

Housekeeping genes

Gene Symbol	Orientation	Oligo sequence (3’ → 5’)
RPL13a	Forward	TGGCCATTTACTTGGTCGTT
	Reverse	GAGCACGGAAATGAAATGG
GAPDH	Forward	CACCTTCTGCAAAATTATGGCG
	Reverse	ACCTTTGCCAAGTCTAACTGTTAA
RPS5	Forward	GATGTTTCTCCGTTACGAGGAGT
	Reverse	GAGTTCATCGGCTAAACATTCGG

SAG vs SLG

Gene Symbol	Orientation	Oligo sequence (3' → 5')
Troponin C type 3b	Forward	CCATTCGGATCCGGGGAAAT
	Reverse	CTGTGTCCGTGTCCTCTTCC
Melittin	Forward	TTACATCTATGCGGCCCCTG
	Reverse	CAATTCCCGCTTCCGGATCT
Flightin	Forward	GTGGAGTGGAGTGGCTATCG
	Reverse	ATATCCCATGGCGCTGGTTC
Hexamerin 110	Forward	ACAACAAGCAGGACAACAGG
	Reverse	ACCAAGTCCGTTAGAAAGACGA
Silk fibronin 4	Forward	GATCCCATCCATACTCGCGG
	Reverse	GTCTCCACCTCTTCCCTTGC
Larva-specific very high-density lipoprotein	Forward	AACGCCTCAATCAGAACGGT
	Reverse	GGATCTACCGAGGTGGCAAG

LAG vs LLG

Gene Symbol	Orientation	Oligo sequence (3' → 5')
Cuticular protein	Forward	AACACCTACCCCTTCTCCAAC
	Reverse	CCGATGGTTTGCGATAAAGAGG
D-galactonate transporter	Forward	TTCATGTGTACGTTCGGCCA
	Reverse	GTAAATCACTTGTGCACAGACACA
Alpha glucosidase 2	Forward	CGGCCAAAACAACAAGGGTT
	Reverse	AATCCATGAAACTGCGGGGA
Hex70c	Forward	TGGACCGATTTGCCGTAACA
	Reverse	TGACGTCGTGAACGGTGATT
DNA ligase 1, Transcript variant X2	Forward	ATCGCGAGCAACGTTCAAAA
	Reverse	ACTTTTGTCCGCGTCTGTTG
Odorant binding protein 13	Forward	TTTGCCTTTTGCCTCGTTGG
	Reverse	TTTCTTCGGCGCAAACTGAC

LLG vs SLG

Gene Symbol	Orientation	Oligo sequence (3’ → 5’)
Peroxidase	Forward	GCCCGGAAAATGAAGACGTG
	Reverse	AATAGGCGATGCCGGTTTCT
Zinc carboxypeptidase	Forward	AGGGGCAGTTCAAGGCTTAC
	Reverse	CTTGGGATGTGAAGCCCCAT
Cuticular protein 14	Forward	GGTGTCCGTGTGACAGAGAG
	Reverse	ACGTCGCTGTCGTACAAAGT
Telomerase reverse transcriptase	Forward	CGTTCAGATACAGCATTGTGTCA
	Reverse	CGCATTGAGAGATGTTTCAGAAGA
Origin recognition complex subunit 5	Forward	GGATGATTGCTATCTTTTGTGCCA
	Reverse	ACACATAGATGGAATCTGTGAGAGT
Yellow-g	Forward	CCACGAGGGCCCAGATATTC
	Reverse	AGTCGAGGATGCCCAAAGTG

LAG vs SAG

Gene Symbol	Orientation	Oligo sequence (3' → 5')
Glucose dehydrogenase [FAD, quinone], transcript variant X2	Forward	CGCCGCTGTATGGATGGATT
	Reverse	GACGGATGGACGAGCTTTCA
Ciliogenesis-associated TTC17-interactiing protein	Forward	TGGTCATTTCGGTGGGTCAA
	Reverse	TCTGTCCTCTTTTCTTCTAGGCAA
Cytochrome b561 domain-containing protein 2	Forward	TGCGTACCATCGGTGAAACA
	Reverse	CGGCATCGATGAGTGTCGTA
Elongation of very long chain fatty acids protein 1	Forward	CGGTGGTGTTCGTGTTGAGA
	Reverse	CGTGGACACGTGATGGTACA
Kinesin-like protein KIF9	Forward	ACGACTAACCAACGAAGTTAATGTT
	Reverse	AGCCTTGGAAATATTCATCAGCG
DNAJ homolog subfamily C member 18-like	Forward	GAAGAAGGAACAAGTGCAAGTTCA
	Reverse	GGAACATGTGCCCAATATTTTCCT

Table S2: List of DEGs for GO_MF category in LLG compared to SLG

Term name	DEGs	p-value
structural constituent of cuticle	**CPR14, CPR13, CPR2, CPR1, LOC102653832, CPR17, LOC724624, CPR21, CPR3,** LOC102654371, CPR5	$2.65x10^{-06}$
sulfate transmembrane transporter activity	**LOC413816, LOC410507, LOC413934, LOC724952**	$3.34x10^{-04}$
secondary active sulfate transmembrane transporter activity	**LOC413816, LOC410507, LOC413934, LOC724952**	$3.34x10^{-04}$
monooxygenase activity	**LOC725159, LOC552418, NOS, LOC408452, Cyp314a1, LOC724175, LOC726418, LOC550965, LOC551179, LOC408650, LOC724860,** Ho	$7.10x10^{-04}$
sulfur compound transmembrane transporter activity	**LOC413816, LOC410507, LOC413934, LOC724952**	$9.70x10^{-04}$
oxidoreductase activity	**LOC725159, LOC406147, LOC552418, LOC552600, LOC409066, LOC413924, LOC552771, LOC411140, LOC406081, LOC551533, LOC550687, LOC411202, NOS, LOC408452, LOC412305, Cyp314a1, LOC724175, LOC726418, LOC412815, LOC552712, LOC550965, LOC551179, Cryl1, LOC551968, LOC408650, LOC724860, LOC725400, LOC551103, LOC409712,** LOC411649, LOC411983, LOC100302106, Ho	$1.53x10^{-03}$
FMN binding	**LOC409066, LOC552771, LOC551533, NOS**	$8.80x10^{-03}$
neuromedin U receptor activity	**LOC411672, LOC726393,** LOC726755	$1.21x10^{-02}$
oxidoreductase activity, acting on paired donors, with incorporation or reduction of molecular oxygen	**LOC725159, LOC552418, NOS, LOC408452, Cyp314a1, LOC724175, LOC726418, LOC550965, LOC551179, LOC724860,** Ho	$1.59x10^{-02}$
neuropeptide receptor activity	**SIFR, LOC411672, LOC726393,** LOC726755	$3.19x10^{-02}$

The table presents the Gene ontology source (MF), term name, associated DEGs, and p-values. DEGs shown in bold are upregulated, while those in normal text are downregulated in LCY-treated larvae group (LLG) compared to solvent-treated larvae group (SLG). The DEGs in each GO term are displayed from highest upregulation to highest downregulation.

Table S3: List of DEGs for GO_MF category in LAG compared to SAG

Term name	DEGs	p-value
tetrapyrrole binding	**LOC724654, LOC408452,** LOC551179	$3.36x10^{-02}$
G protein-coupled peptide receptor activity	**LOC411672, Akhr**	$3.36x10^{-02}$
peptide receptor activity	**LOC411672, Akhr**	$3.36x10^{-02}$
heme binding	**LOC724654, LOC408452,** LOC551179	$3.36x10^{-02}$
flavin adenine dinucleotide binding	**LOC410747, LOC410745,** LOC551044	$3.36x10^{-02}$
oxidoreductase activity	**LOC410747, LOC410745, LOC408452,** LOC551044, LOC551179, LOC100578936	$3.36x10^{-02}$
oxidoreductase activity, acting on CH-OH group of donors	**LOC410747, LOC410745,** LOC551044	$3.36x10^{-02}$
monoamine transmembrane transporter activity	LOC409073	$3.43x10^{-02}$
methylenetetrahydrofolate reductase (NAD(P)H) activity	LOC100578936	$3.43x10^{-02}$

The table presents the Gene ontology source (MF), term name, associated DEGs, and p-values. DEGs shown in bold are upregulated, while those in normal text are downregulated in LCY-treated adult group (LAG) compared to solvent-treated adult group (SAG). The DEGs in each GO term are displayed from highest upregulation to highest downregulation.

Table S4: List of DEGs for GO_CC category in LAG compared to LLG

Term name	DEGs	p-value
mitochondrion	**CytC, LOC100578782, LOC552610, LOC551541, Uqcr11, LOC410557, LOC409292, LOC726120, LOC102654955, LOC408837, LOC726316, LOC725253, LOC552128, LOC411677, LOC413340, LOC551861, LOC726747, LOC727483, LOC413517, LOC724827, LOC409473, LOC100302106, LOC410022, LOC551757, LOC409586, Ndufb2, LOC727599, Cox6b1, LOC725881, LOC724264, Ndufs5, LOC725315, LOC551660, LOC725797, Ant, LOC551042, LOC408511, LOC412396, LOC726902, LOC552009, LOC408446, LOC726617, LOC100576960, LOC725566, LOC410791, LOC727026, LOC411924, LOC726239, LOC726061, LOC551325, LOC725712, LOC552152, LOC551158, LOC412984, Coq7, LOC551329, LOC726731, LOC100577341, LOC411448, LOC551939, LOC413781, LOC408548, LOC552354, LOC410612, LOC102654007, LOC408968, LOC726498, LOC724790, LOC727014, LOC410856, LOC413762, LOC413438, LOC411351, LOC412409, LOC413774, LOC725705, LOC412082, LOC724988, LOC551337, LOC552802, LOC552644, LOC551492, LOC550915, LOC725854, LOC413228, LOC552635, LOC411304, LOC411142, LOC408569, LOC725240, LOC408734, LOC552526, LOC551523, LOC413186, LOC413879, LOC408352, LOC413878, LOC100576140,** LOC410308, LOC725527, LOC551710, LOC410325, LOC410410, LOC411790, LOC409667, LOC408539, LOC100576847, LOC412308, LOC102655740	$1.25x10^{-12}$
cytoplasm	**CytC, LOC107964319, LOC100578782, LOC552610, LOC551541, Uqcr11, LOC410557, LOC409292, LOC726120, sGC-alpha1, LOC102654955, LOC408837, LOC726316, Ef-1a-f1, LOC725253, LOC552128, LOC411677, LOC413340, LOC551861, LOC726747, LOC727483, LOC413517, LOC724827, LOC409473, LOC100302106, Gycbeta1, LOC410022, LOC408614, LOC410371, LOC551757, LOC409586, LOC551631, Ndufb2, LOC727599, Cox6b1, LOC725881, LOC724264, Ndufs5, LOC725315, TpnT, LOC551660, LOC725797, TpnI, Ant, LOC551042, LOC408511, LOC412396, LOC726902, LOC552009, LOC413139, LOC102655754, LOC408446, LOC726617, LOC100576960, LOC725566, LOC410791, LOC727026, LOC411924, LOC726239, LOC409299, LOC726061, LOC102654353, LOC551325, LOC725712, LOC552152, LOC551158, LOC412984, Coq7, LOC551329, LOC726731, LOC100576735,**	$1.34x10^{-07}$

LOC100577341, LOC411448, LOC412886, LOC551939, Syt1, LOC413781, LOC408548, LOC100578168, LOC552519, LOC552354, LOC552181, LOC410612, LOC102654007, LOC408968, LOC726498, Glob1, LOC724790, Cry2, LOC727014, LOC410856, LOC413762, LOC413438, LOC411351, LOC100578006, LOC412409, LOC552745, LOC413774, LOC725977, LOC100576099, LOC100579024, LOC725705, LOC551978, LOC412082, LOC724988, LOC413889, LOC551337, LOC551216, LOC724241, LOC552705, LOC724496, LOC552802, LOC552644, LOC551961, LOC107964213, LOC551492, LOC550915, LOC725854, LOC413228, LOC552635, LOC726815, LOC411304, LOC411142, LOC408569, LOC725240, LOC726164, LOC408734, LOC411654, LOC100577378, LOC725134, LOC551354, LOC727186, LOC726894, LOC552526, LOC411459, LOC409055, LOC100577548, LOC551523, LOC725194, LOC551477, LOC551580, LOC102655898, LOC413186, LOC410922, LOC413879, LOC552533, LOC412710, LOC408352, LOC412119, LOC552256, LOC727300, LOC552377, LOC550686, LOC550700, LOC550975, LOC726205, LOC726609, LOC409015, LOC409487, LOC725680, LOC409934, LOC413878, LOC102655877, LOC412085, LOC100576140, LOC551101, LOC726176, LOC725742, LOC410889, LOC410298, LOC552260, LOC411237, LOC410348, LOC408392, sGCbeta-3, LOC551057, LOC725135, LOC411595, LOC550673, LOC411870, LOC551408, LOC550806, LOC552766, LOC724262, LOC409134, LOC551436, LOC551420, LOC410308, LOC551184, LOC412842, LOC409724, LOC550694, LOC410207, LOC551578, LOC725018, LOC552118, LOC725817, LOC551802, LOC725358, LOC408418, LOC725527, LOC410533, LOC408751, LOC552540, LOC410510, LOC409165, LOC409301, LOC413299, LOC551535, LOC413614, LOC409321, LOC726301, LOC412169, LOC412511, LOC724741, LOC411765, LOC409581, LOC726419, LOC551438, LOC408301, Gpdh, LOC551472, LOC552560, LOC406152, LOC409543, LOC410869, LOC551710, LOC411533, EF1a-F2, LOC552346, LOC102653815, LOC411799, LOC408388, LOC100576432, LOC410808, Ripk5, LOC408687, LOC100576667, LOC550798, LOC411923, nanos, LOC410325, LOC410410, LOC409809, LOC552363, Arp1, LOC411790, LOC412823, LOC411088, LOC409485, LOC413742, LOC724947, LOC725789, LOC550827, LOC413613, LOC408969, LOC726887, LOC412608, LOC550716, LOC408808, LOC412097, LOC409313, LOC409667, LOC409576, LOC551282, LOC408539, LOC100576847,

	LOC412308, LOC551466, LOC100576957, PRF, LOC409520, LOC102655740, LOC409023, LOC411378, LOC551986, LOC408987, LOC412544, LOC408650, LOC411147, LOC552313, LOC409435, LOC410994, Hex110	
mitochondrial inner membrane	**LOC100578782, LOC552610, LOC551541, Uqcr11, LOC410557, LOC726120, LOC102654955, LOC408837, LOC726316, LOC725253, LOC411677, LOC413340, LOC551861, LOC726747, LOC727483, LOC413517, LOC409473, LOC551757, Ndufb2, LOC727599, LOC725881, LOC724264, Ant, LOC551042, LOC412396, LOC726902, LOC552009, LOC725566, LOC725712, Coq7, LOC413781, LOC408548, LOC102654007, LOC408968, LOC726498, LOC412409, LOC725705, LOC412082, LOC551337, LOC408569, LOC408734, LOC413186,** LOC725527, LOC411790	$1.20x10^{-05}$
organelle inner membrane	**LOC100578782, LOC552610, LOC551541, Uqcr11, LOC410557, LOC726120, LOC102654955, LOC408837, LOC726316, LOC725253, LOC411677, LOC413340, LOC551861, LOC726747, LOC727483, LOC413517, LOC409473, LOC551757, Ndufb2, LOC727599, LOC725881, LOC724264, Ant, LOC551042, LOC412396, LOC726902, LOC552009, LOC725566, LOC725712, Coq7, LOC413781, LOC408548, LOC102654007, LOC408968, LOC726498, LOC412409, LOC725705, LOC412082, LOC551337, LOC408569, LOC408734, LOC413186,** LOC725527, LOC411790	$1.20x10^{-05}$
mitochondrial envelope	**CytC, LOC100578782, LOC552610, LOC551541, Uqcr11, LOC410557, LOC726120, LOC102654955, LOC408837, LOC726316, LOC725253, LOC411677, LOC413340, LOC551861, LOC726747, LOC727483, LOC413517, LOC409473, LOC551757, Ndufb2, LOC727599, LOC725881, LOC724264, Ant, LOC551042, LOC408511, LOC412396, LOC726902, LOC552009, LOC725566, LOC410791, LOC727026, LOC726061, LOC551325, LOC725712, Coq7, LOC413781, LOC408548, LOC552354, LOC102654007, LOC408968, LOC726498, LOC412409, LOC725705, LOC412082, LOC551337, LOC550915, LOC411142, LOC408569, LOC408734, LOC551523, LOC413186,** LOC725527, LOC411790, LOC409667, LOC408539, LOC102655740	$1.20x10^{-05}$

organelle envelope	**CytC, LOC100578782, LOC552610, LOC551541, Uqcr11, LOC410557, LOC726120, LOC102654955, LOC408837, LOC726316, LOC725253, LOC411677, LOC413340, LOC551861, LOC726747, LOC727483, LOC413517, LOC409473, LOC551757, Ndufb2, LOC727599, LOC725881, LOC724264, Ant, LOC551042, LOC408511, LOC412396, LOC726902, LOC552009, LOC725566, LOC410791, LOC727026, LOC726061, LOC551325, LOC725712, Coq7, LOC413781, LOC408548, LOC552354, LOC102654007, LOC408968, LOC726498, LOC412409, LOC725705, LOC412082, LOC551337, LOC550915, LOC411142, LOC408569, LOC408734, LOC551523, LOC413186,** LOC412464, LOC725527, LOC411918, LOC410343, LOC413675, LOC411790, LOC409667, LOC408539, LOC102655740	3.75x10^{-05}
envelope	**CytC, LOC100578782, LOC552610, LOC551541, Uqcr11, LOC410557, LOC726120, LOC102654955, LOC408837, LOC726316, LOC725253, LOC411677, LOC413340, LOC551861, LOC726747, LOC727483, LOC413517, LOC409473, LOC551757, Ndufb2, LOC727599, LOC725881, LOC724264, Ant, LOC551042, LOC408511, LOC412396, LOC726902, LOC552009, LOC725566, LOC410791, LOC727026, LOC726061, LOC551325, LOC725712, Coq7, LOC413781, LOC408548, LOC552354, LOC102654007, LOC408968, LOC726498, LOC412409, LOC725705, LOC412082, LOC551337, LOC550915, LOC411142, LOC408569, LOC408734, LOC551523, LOC413186,** LOC412464, LOC725527, LOC411918, LOC410343, LOC413675, LOC411790, LOC409667, LOC408539, LOC102655740	3.75x10^{-05}
mitochondrial membrane	**LOC100578782, LOC552610, LOC551541, Uqcr11, LOC410557, LOC726120, LOC102654955, LOC408837, LOC726316, LOC725253, LOC411677, LOC413340, LOC551861, LOC726747, LOC727483, LOC413517, LOC409473, LOC551757, Ndufb2, LOC727599, LOC725881, LOC724264, Ant, LOC551042, LOC412396, LOC726902, LOC552009, LOC725566, LOC410791, LOC551325, LOC725712, Coq7, LOC413781, LOC408548, LOC102654007, LOC408968, LOC726498, LOC412409, LOC725705, LOC412082, LOC551337, LOC550915, LOC411142,**	2.68x10^{-04}

	**LOC408569, LOC408734, LOC413186,** LOC725527, LOC411790, LOC408539, LOC102655740	
organelle membrane	**LOC100578782, LOC552610, LOC551541, Uqcr11, LOC410557, LOC726120, LOC102654955, LOC408837, LOC726316, LOC725253, LOC411677, LOC413340, LOC551861, LOC726747, LOC727483, LOC413517, LOC409473, LOC551757, Ndufb2, LOC727599, LOC725881, LOC724264, Ant, LOC551042, LOC412396, LOC726902, LOC552009, LOC725566, LOC410791, LOC102654353, LOC551325, LOC725712, Coq7, LOC100576735, LOC413781, LOC408548, LOC100578168, LOC102654007, LOC408968, LOC726498, LOC412409, LOC725977, LOC100576099, LOC725705, LOC412082, LOC551337, LOC552705, LOC551961, LOC550915, LOC726815, LOC411142, LOC408569, LOC726164, LOC408734, LOC727186, LOC726894, LOC411459, LOC409055, LOC551477, LOC102655898, LOC413186, LOC552533, LOC552256, LOC552377, LOC550975, LOC409934, LOC102655877, LOC412085, LOC725742, LOC410889, LOC410298,** LOC725135, LOC411595, LOC551408, LOC550806, LOC724262, LOC551420, LOC551578, LOC725527, LOC724741, LOC726419, LOC408301, LOC406152, LOC410869, LOC413675, LOC552346, LOC102653815, LOC411799, LOC100576667, LOC411790, LOC724947, LOC725789, LOC413613, LOC550716, LOC408539, LOC551466, LOC100576957, LOC102655740, LOC411147, LOC552313	$7.36x10^{-03}$
respirasome	**CytC, LOC100578782, LOC552610, LOC551541, Uqcr11, LOC408837, LOC726316, LOC413340, LOC726747, LOC409473, LOC551757, Ndufb2, LOC727599, Cox6b1, LOC551042, LOC412396, LOC409549, LOC725712,** LOC725527, LOC100576847	$7.36x10^{-03}$

The table presents the Gene ontology source (CC), term name, associated DEGs, and p-values. DEGs shown in bold are upregulated, while those in normal text are downregulated in LCY-treated adult group (LAG) compared to LCY-treated larvae group (LLG). The DEGs in each GO term are displayed from highest upregulation to highest downregulation.

Table S5: List of DEGs for GO_CC category in SAG compared to SLG

Term name	Differentially Expressed Genes	P-Value
mitochondrion	**CytC, LOC552610, LOC551541, Uqcr11, LOC408837, LOC410557, LOC409292, LOC726316, LOC726120, LOC102654955, LOC727483, LOC725253, LOC552128, LOC413340, LOC100578782, LOC413517, LOC410022, LOC409473, Ndufs5, LOC726747, LOC551861, LOC411677, LOC724827, LOC551757, LOC409586, LOC725797, LOC724264, LOC727599, LOC551042, LOC725881, LOC551660, LOC552009, Cox6b1, LOC412396, Ndufb2, LOC725315, LOC725566, LOC408446, Ant, LOC100302106, LOC726902, LOC408511, LOC726239, LOC727026, LOC100576960, LOC552152, LOC725712, LOC551325, LOC410791, LOC411924, LOC551158, LOC412984, LOC726617, LOC726061, LOC726731, LOC413781, LOC727014, LOC551939, LOC102654007, LOC408548, LOC408968, Coq7, LOC411448, LOC410612, LOC413186, LOC411351, LOC410856, LOC724790, LOC412409, LOC413762, LOC552354, LOC413438, LOC725854, LOC551329, LOC550915, LOC412082, LOC413774, LOC100577341, LOC725705, LOC726498, LOC551337, LOC552526, LOC552644, LOC552802, LOC411142, LOC408352, LOC411304, LOC412796, LOC408569, LOC551523, LOC413228, LOC413879, LOC552635, LOC724988, LOC725240, LOC408734, LOC413878, LOC551492, LOC725105,** LOC551710, LOC410325, LOC725527, LOC408539, LOC411790, LOC409667, LOC410410, LOC102655740, LOC412308, LOC100576847	$7.95x10^{-13}$
mitochondrial inner membrane	**LOC552610, LOC551541, Uqcr11, LOC408837, LOC410557, LOC726316, LOC726120, LOC102654955, LOC727483, LOC725253, LOC413340, LOC100578782, LOC413517, LOC409473, LOC726747, LOC551861, LOC411677, LOC551757, LOC724264, LOC727599, LOC551042, LOC725881, LOC552009, LOC412396, Ndufb2, LOC725566, Ant, LOC726902, LOC725712, LOC413781, LOC102654007, LOC408548, LOC408968, Coq7, LOC413186, LOC412409, LOC412082, LOC725705, LOC726498, LOC551337, LOC408569, LOC408734, LOC725105,** LOC725527, LOC411790	$3.40x10^{-06}$

organelle inner membrane	**LOC552610, LOC551541, Uqcr11, LOC408837, LOC410557, LOC726316, LOC726120, LOC102654955, LOC727483, LOC725253, LOC413340, LOC100578782, LOC413517, LOC409473, LOC726747, LOC551861, LOC411677, LOC551757, LOC724264, LOC727599, LOC551042, LOC725881, LOC552009, LOC412396, Ndufb2, LOC725566, Ant, LOC726902, LOC725712, LOC413781, LOC102654007, LOC408548, LOC408968, Coq7, LOC413186, LOC412409, LOC412082, LOC725705, LOC726498, LOC551337, LOC408569, LOC408734, LOC725105,** LOC725527, LOC411790	$3.40x10^{-06}$
mitochondrial envelope	**CytC, LOC552610, LOC551541, Uqcr11, LOC408837, LOC410557, LOC726316, LOC726120, LOC102654955, LOC727483, LOC725253, LOC413340, LOC100578782, LOC413517, LOC409473, LOC726747, LOC551861, LOC411677, LOC551757, LOC724264, LOC727599, LOC551042, LOC725881, LOC552009, LOC412396, Ndufb2, LOC725566, Ant, LOC726902, LOC408511, LOC727026, LOC725712, LOC551325, LOC410791, LOC726061, LOC413781, LOC102654007, LOC408548, LOC408968, Coq7, LOC413186, LOC412409, LOC552354, LOC550915, LOC412082, LOC725705, LOC726498, LOC551337, LOC411142, LOC408569, LOC551523, LOC408734, LOC725105,** LOC725527, LOC408539, LOC411790, LOC409667, LOC102655740	$3.40x10^{-06}$
organelle envelope	**CytC, LOC552610, LOC551541, Uqcr11, LOC408837, LOC410557, LOC726316, LOC726120, LOC102654955, LOC727483, LOC725253, LOC413340, LOC100578782, LOC413517, LOC409473, LOC726747, LOC551861, LOC411677, LOC551757, LOC724264, LOC727599, LOC551042, LOC725881, LOC552009, LOC412396, Ndufb2, LOC725566, Ant, LOC726902, LOC408511, LOC727026, LOC725712, LOC551325, LOC410791, LOC726061, LOC413781, LOC102654007, LOC408548, LOC408968, Coq7, LOC413186, LOC412409, LOC552354, LOC550915, LOC412082, LOC725705, LOC726498, LOC551337, LOC411142, LOC408569, LOC551523, LOC408734, LOC725105,** LOC410972, LOC410343, LOC725527, LOC408539, LOC412464, LOC411790, LOC413675, LOC411918, LOC409667, LOC102655740	$4.71x10^{-06}$
envelope	**CytC, LOC552610, LOC551541, Uqcr11, LOC408837, LOC410557, LOC726316, LOC726120, LOC102654955, LOC727483, LOC725253, LOC413340, LOC100578782, LOC413517, LOC409473, LOC726747, LOC551861, LOC411677, LOC551757, LOC724264, LOC727599, LOC551042, LOC725881, LOC552009, LOC412396, Ndufb2, LOC725566, Ant, LOC726902, LOC408511, LOC727026, LOC725712, LOC551325, LOC410791, LOC726061, LOC413781, LOC102654007, LOC408548,**	$4.71x10^{-06}$

	**LOC408968, Coq7, LOC413186, LOC412409, LOC552354, LOC550915, LOC412082, LOC725705, LOC726498, LOC551337, LOC411142, LOC408569, LOC551523, LOC408734, LOC725105,** LOC410972, LOC410343, LOC725527, LOC408539, LOC412464, LOC411790, LOC413675, LOC411918, LOC409667, LOC102655740	
cytoplasm	**CytC, LOC107964319, LOC552610, LOC551541, Uqcr11, sGC-alpha1, LOC408837, LOC410557, LOC409292, LOC726316, TpnT, LOC726120, LOC102654955, LOC727483, LOC725253, LOC552128, LOC413340, LOC100578782, Gycbeta1, LOC413517, LOC410022, LOC409473, Ndufs5, Ef-1a-f1, LOC726747, LOC551861, LOC411677, LOC724827, LOC551757, LOC409586, LOC725797, LOC724264, LOC727599, LOC410371, LOC551042, LOC725881, TpnI, LOC551631, LOC551660, LOC408614, LOC552009, Cox6b1, LOC412396, Ndufb2, LOC725566, LOC408446, Ant, LOC100302106, Glob1, LOC102654353, LOC726902, LOC408511, LOC726239, LOC727026, LOC100576960, LOC413139, LOC552152, LOC725712, LOC409299, LOC551325, LOC410791, LOC411924, LOC102655754, LOC551158, LOC100576735, LOC412886, LOC412984, LOC100577548, LOC726617, Syt1, Cry2, LOC726061, LOC726731, LOC413781, LOC410996, LOC727014, LOC551939, LOC102654007, LOC408548, LOC408968, Coq7, LOC411448, LOC552519, LOC410612, LOC413186, LOC411351, LOC410856, LOC100578006, LOC724790, LOC412409, LOC413762, LOC552354, LOC413438, LOC725854, LOC551329, LOC550915, LOC412082, LOC413774, LOC552745, LOC100577341, LOC725705, LOC551216, LOC551978, LOC551961, LOC551354, LOC724241, LOC726498, LOC551337, LOC726164, LOC552526, LOC552644, LOC552181, LOC725977, LOC409055, LOC411459, LOC725680, LOC552802, LOC411142, LOC724496, LOC409905, LOC413889, LOC552014, LOC100579024, LOC552705, LOC408352, LOC411304, LOC552377, LOC413601, LOC107964213, LOC412796, LOC102655877, LOC408569, LOC551523, LOC413228, LOC726176, LOC725194, LOC550686, LOC413879, LOC552635, LOC724988, LOC726894, LOC552533, LOC411654, LOC725240, LOC100577378, LOC726815, LOC102655898, LOC551580, LOC551477, LOC409487, LOC409934, LOC412119, LOC412710, LOC408734, LOC727300, LOC724188, LOC550700, LOC552001, LOC409015, LOC726205, LOC725742, LOC413878, LOC410922, LOC102656006, LOC551492, LOC408391, LOC102656491, LOC725105, LOC726609, LOC552256**, LOC552540, LOC726132, LOC551184, LOC410533, LOC412169,	6.89×10^{-05}

	LOC410614, LOC409718, LOC551408, LOC550673, LOC724505, LOC551436, LOC552118, LOC551710, LOC552140, LOC411870, LOC410486, LOC413299, LOC551420, LOC551771, LOC409321, LOC550694, Gpdh, LOC551472, LOC409301, LOC552346, LOC551802, LOC411533, LOC411970, LOC408751, LOC412511, LOC552363, LOC410869, LOC102653815, LOC411765, LOC725817, LOC411799, LOC413614, LOC410325, LOC550798, LOC551438, LOC552766, LOC551578, LOC408687, LOC725018, LOC725527, LOC408539, LOC726546, LOC726419, LOC410808, LOC100576432, LOC408301, LOC412608, LOC408388, LOC550716, LOC724741, EF1a-F2, LOC411790, LOC409543, LOC100576667, LOC409313, Arp1, LOC551466, LOC552560, LOC551282, LOC408808, Ripk5, LOC413613, LOC409809, LOC409485, LOC413742, LOC412823, LOC725789, LOC551986, LOC411088, nanos, LOC412097, LOC409576, LOC550827, LOC411378, LOC409667, LOC724947, LOC410410, LOC409023, LOC102655740, PRF, LOC726887, LOC411147, LOC412544, LOC408650, LOC412308, LOC100576847, LOC409520, LOC408987, LOC409435, LOC552313, LOC410994, Hex110.	
mitochondrial membrane	**LOC552610, LOC551541, Uqcr11, LOC408837, LOC410557, LOC726316, LOC726120, LOC102654955, LOC727483, LOC725253, LOC413340, LOC100578782, LOC413517, LOC409473, LOC726747, LOC551861, LOC411677, LOC551757, LOC724264, LOC727599, LOC551042, LOC725881, LOC552009, LOC412396, Ndufb2, LOC725566, Ant, LOC726902, LOC725712, LOC551325, LOC410791, LOC413781, LOC102654007, LOC408548, LOC408968, Coq7, LOC413186, LOC412409, LOC550915, LOC412082, LOC725705, LOC726498, LOC551337, LOC411142, LOC408569, LOC408734, LOC725105,** LOC725527, LOC408539, LOC411790, LOC102655740	$8.24x10^{-05}$
respirasome	**CytC, LOC552610, LOC551541, Uqcr11, LOC408837, LOC726316, LOC413340, LOC100578782, LOC409473, LOC726747, LOC551757, LOC727599, LOC551042, Cox6b1, LOC412396, Ndufb2, LOC409549, LOC725712,** LOC725527, LOC100576847	$6.93x10^{-03}$
respiratory chain complex	**LOC552610, LOC551541, Uqcr11, LOC408837, LOC726316, LOC413340, LOC100578782, LOC726747, LOC551757, LOC551042, Cox6b1, LOC412396, Ndufb2, LOC409549, LOC725712,** LOC100576847	$7.72x10^{-03}$

The table presents the Gene ontology source (CC), term name, associated DEGs, and p-values. DEGs shown in bold are upregulated, while those in normal text are downregulated in solvent-treated adult group (SAG) compared to solvent-treated larvae group (SLG). The DEGs in each GO term are displayed from highest upregulation to highest downregulation.

Table S6: List of DEGs for GO_MF category in LAG compared to LLG

Term name	DEGs	p-value
structural constituent of cuticle	**CPR19, CPR6, CPR5, LOC724624, CPR14, LOC727578, CPR27, LOC726725, LOC107964828, CPR3, LOC726950, CPR11, LOC552217,** LOC726451, CPR16, LOC102654371, CPR2, CPR12, LOC102653832, CPR13, CPR1, CPR21	$3.33x10^{-05}$
transmembrane transporter activity	**Melt, LOC412925, LOC724608, LOC550691, LOC725346, LOC725165, LOC552610, LOC551541, LOC551604, LCCH3, Hiscl1, Uqcr11, LOC410557, LOC726120, LOC102654955, ATP5G2, LOC551680, LOC408837, LOC726316, LOC412777, LOC412220, LOC411411, LOC409236, LOC551861, LOC726747, LOC727483, LOC413517, LOC411732, LOC724827, LOC552682, LOC551766, Eaat-2, Gat-1B, LOC406124, LOC410371, GluCl, LOC408909, LOC552699, LOC725881, LOC724264, LOC725315, LOC551660, LOC551782, LOC102653800, LOC408525, Ant, LOC412396, LOC100578557, LOC409114, Gat-a, LOC408769, LOC409919, Nmdar1, LOC410788, LOC410791, LOC551454, nAChRa7, LOC551325, LOC552792, LOC551167, LOC411764, LOC409715, pHCl, LOC409056, LOC725219, LOC409073, LOC410805, nAChRa8, LOC410803, LOC410612, LOC412431, LOC552149, LOC100578899, nAChRa2, LOC100578218, nAChRa6, LOC409646, LOC413816, LOC551337, Para, LOC726423, LOC410278, LOC412007, LOC100577376, LOC413959, LOC411052, LOC552727, LOC551961, LOC550915, LOC551883, LOC409932, LOC409924, LOC409565, LOC552744, LOC409055, LOC409897, LOC410626, LOC411036, LOC552704, LOC552476, LOC552708, LOC408402, LOC412830, LOC551093, LOC551936, LOC413844, LOC726721, LOC409074,** LOC408456, LOC413551, LOC551894, LOC412056, LOC412678, LOC725527, LOC409424, LOC411729, LOC552653, LOC552144, LOC726724, LOC411997, LOC408517, LOC551863, LOC410296, LOC410915, SsRbeta, LOC410507, LOC412741, LOC551553, LOC411790, LOC410705, LOC100578207, LOC408478, nAChRb2, LOC410062, LOC413259, LOC551263, LOC413739, LOC552592, LOC412430, LOC408634, LOC551806, LOC408841, Dat, LOC550918, LOC411935, LOC724952, LOC408539, LOC409467, LOC408777, LOC408467, LOC102655740, LOC410578, LOC100578810, LOC551845, LOC414051, LOC408828, LOC726412, LOC727346, LOC412764, LOC410967, nAChRa9, LOC726762, LOC725462, LOC725922	$5.34x10^{-05}$

transporter activity	**Melt, Vg, LOC412925, LOC724608, LOC550691, LOC408696, LOC725346, LOC725165, LOC552610, LOC551541, LOC551604, LCCH3, Hiscl1, Uqcr11, LOC410557, LOC726120, LOC102654955, ATP5G2, LOC551680, LOC408837, LOC726316, LOC412777, LOC412220, LOC411411, LOC409236, LOC551861, LOC726747, LOC727483, LOC413517, LOC411732, LOC724827, LOC552682, LOC551766, Eaat-2, Gat-1B, LOC406124, LOC410371, GluCl, LOC726793, LOC408909, LOC552699, LOC725881, LOC724264, LOC725315, LOC551660, LOC551782, LOC102653800, LOC408525, Ant, LOC412396, LOC100578557, LOC409114, Gat-a, LOC408769, LOC409919, Nmdar1, LOC410788, LOC410791, LOC551454, nAChRa7, LOC551325, LOC552792, LOC551167, LOC411764, LOC409715, pHCl, LOC409056, LOC725219, LOC409073, LOC410805, nAChRa8, LOC410803, LOC410612, LOC412431, LOC552149, LOC100578899, nAChRa2, LOC100578218, nAChRa6, LOC409646, LOC413816, LOC551337, Para, LOC726423, LOC410278, LOC412007, LOC409192, LOC100577376, LOC413959, LOC411052, LOC552727, LOC551961, LOC550915, LOC551883, LOC409932, LOC409924, LOC409565, LOC552744, LOC409055, LOC409897, LOC410626, LOC411036, LOC552704, LOC552476, LOC552708, LOC408402, LOC412830, LOC551093, LOC551936, LOC413844, LOC726721, LOC409074,** LOC408456, LOC413551, LOC551894, LOC412056, LOC412678, LOC725527, LOC410533, LOC409424, LOC411729, LOC552653, LOC552144, LOC726724, LOC411997, LOC408517, LOC551863, LOC410296, LOC410915, SsRbeta, LOC410507, LOC412741, LOC551553, LOC411790, LOC410705, LOC100578207, LOC408478, nAChRb2, LOC410062, LOC413259, LOC551263, LOC413739, LOC552592, LOC412430, LOC408634, LOC551806, LOC408841, Dat, LOC550918, LOC411935, LOC724952, LOC411955, LOC408539, LOC409467, LOC408777, LOC408467, LOC102655740, LOC410578, LOC100578810, LOC551845, LOC414051, LOC551250, LOC408828, LOC726412, LOC727346, LOC412764, LOC410967, nAChRa9, LOC726762, LOC725462, LOC725922, Vhdl	5.34×10^{-05}
electron transfer activity	**CytC, LOC552610, LOC551541, LOC413605, Uqcr11, LOC408837, LOC726316, LOC411411, LOC726747, LOC724827, LOC408909, LOC725881, LOC724264, LOC725315, LOC551660, LOC412396,**	1.55×10^{-02}

	**LOC409549, LOC552835, LOC100578218, LOC552105,** LOC410308, LOC552343, LOC725527, LOC551710	
oxidoreductase activity	**TyHyd, LOC552610, LOC726441, LOC551541, Uqcr11, LOC551039, LOC406081, LOC551527, LOC409292, LOC408837, LOC410492, LOC726316, LOC413478, LOC552128, LOC411411, LOC413891, CYP6AQ1, LOC726747, LOC412163, LOC724827, LOC100302106, LOC408909, LOC409793, LOC551078, LOC727599, LOC725881, LOC724264, LOC408950, LOC725315, LOC551660, LOC410396, LOC551103, LOC412396, LOC409549, Sod2, LOC725159, LOC552429, LOC552835, LOC551545, LOC410828, LOC410122, LOC726260, LOC552316, Tpx-3, Coq7, LOC724791, LOC408452, LOC410120, LOC726337, LOC412986, LOC410023, LOC552679, LOC100578218, LOC409253, LOC409682, LOC409642, LOC107963973, LOC411189, LOC552635, LOC411304, Tbh, LOC408734, LOC725400, LOC552130, LOC410763, LOC408643, LOC411649, Sod1, LOC725325, LOC724451, LOC413141, LOC413098, LOC724560, LOC107965322, LOC411983, LOC411014,** LOC550673, LOC552732, PPO, LOC408398, LOC552343, LOC411863, LOC411202, LOC410207, LOC413567, LOC409773, LOC725527, LOC551535, LOC412548, LOC552692, LOC409712, LOC724860, LOC726418, LOC413740, LOC413356, Gpdh, LOC413924, Pten, LOC410617, LOC102653815, LOC551197, LOC409158, LOC412815, LOC412305, LOC410325, LOC725274, LOC725287, LOC410254, LOC409847, LOC550687, LOC408859, LOC551849, LOC408567, LOC552771, LOC409360, LOC409736, LOC411378, LOC410422, LOC412209, LOC551044, LOC408650, LOC409066, LOC725967, LOC724348, LOC406147, LOC411140, LOC411893, LOC409207, Cyp314a1, LOC552418, LOC551179, LOC724175, LOC550965	1.78×10^{-02}
passive transmembrane transporter activity	**Melt, LOC724608, LOC725165, LOC551604, LCCH3, Hiscl1, LOC551680, LOC409236, LOC727483, LOC411732, LOC552682, LOC551766, LOC406124, GluCl, LOC552699, LOC551782, LOC102653800, LOC408525, LOC409114, Nmdar1, LOC410788, LOC551454, nAChRa7, LOC551325, LOC552792, LOC411764, pHCl, LOC725219, LOC410805, nAChRa8, LOC552149, LOC100578899,**	1.78×10^{-02}

	**nAChRa2, nAChRa6, Para, LOC726423, LOC100577376, LOC551883, LOC411036, LOC412830,** LOC551894, LOC726724, LOC410915, SsRbeta, LOC100578207, nAChRb2, LOC413259, LOC551806, LOC408841, LOC408777, LOC408467, LOC551845, LOC727346, nAChRa9	
channel activity	**Melt, LOC724608, LOC725165, LOC551604, LCCH3, Hiscl1, LOC551680, LOC409236, LOC727483, LOC411732, LOC552682, LOC551766, LOC406124, GluCl, LOC552699, LOC551782, LOC102653800, LOC408525, LOC409114, Nmdar1, LOC410788, LOC551454, nAChRa7, LOC551325, LOC552792, LOC411764, pHCl, LOC725219, LOC410805, nAChRa8, LOC552149, LOC100578899, nAChRa2, nAChRa6, Para, LOC726423, LOC100577376, LOC551883, LOC411036, LOC412830,** LOC551894, LOC726724, LOC410915, SsRbeta, LOC100578207, nAChRb2, LOC413259, LOC551806, LOC408841, LOC408777, LOC408467, LOC551845, LOC727346, nAChRa9	$1.78x10^{-02}$
active transmembrane transporter activity	**LOC725346, LOC552610, LOC551541, Uqcr11, LOC408837, LOC726316, LOC412777, LOC411411, LOC726747, LOC413517, LOC724827, Eaat-2, Gat-1B, LOC410371, LOC408909, LOC725881, LOC724264, LOC725315, LOC551660, Ant, LOC412396, Gat-a, LOC408769, LOC409919, LOC551167, LOC409715, LOC409056, LOC409073, LOC100578218, LOC413816, LOC551337, LOC413959, LOC551961, LOC552744, LOC409055, LOC409897, LOC552476, LOC551093, LOC413844, LOC726721, LOC409074,** LOC725527, LOC411729, LOC411997, LOC410507, LOC412741, Dat, LOC550918, LOC724952, LOC410578, LOC414051, LOC410967	$1.99x10^{-02}$
structural molecule activity	**CPR19, CPR6, CPR5, LOC724624, CPR14, LOC727578, CPR27, LOC726725, LOC724629, LOC107964828, LOC100578551, CPR3, LOC726950, CPR11, LOC724263, LOC411924, LOC725659, LOC408675, LOC411103, LOC724164, LOC725291, LOC551158, LOC724744, LOC552097, LOC412984, LOC551584, LOC100577702, LOC724125, LOC100578560, LOC412886, LOC551939, LOC411862, LOC725062, LOC724790, LOC409951, LOC412367, LOC100578704, LOC413928, LOC412549, LOC413774, LOC412473, LOC552676, LOC552628, LOC725866, LOC725854, Rpl39, LOC552217, LOC724708, LOC552526,**	$2.19x10^{-02}$

	**LOC409723, LOC100577548, LOC411618, LOC724467, LOC552616, LOC552593, LOC726151, LOC551867, LOC552345,** LOC411595, LOC550806, LOC552766, RpL32, LOC413365, LOC726451, LOC412464, LOC551779, LOC725308, LOC410343, LOC413675, LOC552850, CPR16, LOC408388, LOC102654371, LOC550827, LOC550716, LOC725450, CPR2, LOC726468, CPR12, LOC102653832, LOC410994, CPR13, CPR1, CPR21	
NAD binding	**LOC409292, LOC552128, LOC413891, LOC410122, LOC409682, LOC725056,** LOC410234, LOC411917, LOC413728, LOC413356, Gpdh, LOC413924, LOC412846, LOC409158, LOC410325, LOC552095, LOC725967	2.35×10^{-02}

The table presents the Gene ontology source (MF), term name, associated DEGs, and p-values. DEGs shown in bold are upregulated, while those in normal text are downregulated in LCY-treated adult group (LAG) compared to LCY-treated larvae group (LLG). The DEGs in each GO term are displayed from highest upregulation to highest downregulation.

Table S7: List of DEGs for GO_MF category in SAG compared to SLG

Term name	Differentially Expressed Genes	P-Value
transporter activity	**Melt, Vg, LOC412925, LOC725165, LOC408696, LOC552610, LOC551541, LOC725346, Uqcr11, LOC551604, LCCH3, Hiscl1, ATP5G2, LOC408837, LOC410557, LOC412777, LOC412220, LOC726316, LOC726120, LOC102654955, LOC727483, LOC411411, LOC409236, Eaat-2, LOC413517, LOC552682, LOC726747, LOC551766, Gat-1B, LOC551861, LOC724827, LOC412007, LOC408992, LOC409646, LOC552699, LOC406124, LOC408909, LOC411732, LOC724264, GluCl, LOC410371, LOC725881, LOC551680, Amel_8916, LOC551660, LOC552792, LOC412396, LOC725315, LOC102653800, Ant, LOC409114, LOC551782, Gat-a, Nmdar1, LOC726793, LOC409073, LOC410788, LOC408769, LOC100578557, LOC551454, nAChRa7, LOC409919, LOC551325, LOC410791, LOC413816, LOC410803, LOC409924, LOC551167, LOC410805, nAChRb1, LOC409636, nAChRa8, LOC408525, LOC409715, LOC410278, LOC100578218, LOC410612, pHCl, LOC409056, LOC411764, Para, LOC409192, LOC550915, LOC409565, nAChRa2, LOC552149, LOC551961, LOC725219, LOC413959, LOC409932, LOC551936, LOC410566, LOC412431, LOC551337, LOC551093, LOC409055, LOC551883, LOC411036, LOC552727, LOC408402, LOC410626, LOC411052, LOC100578899, LOC552476, LOC409897, LOC413020, LOC410997, LOC552720, LOC412830, LOC726423, LOC409899, LOC552704, LOC409666, LOC409074, LOC552744, LOC413934, nAChRa6, LOC552001, LOC552410, LOC409865, LOC726721, LOC413689, LOC552708,** LOC410062, LOC410533, LOC551894, LOC411403, LOC726513, LOC552552, LOC411807, LOC412741, LOC409424, LOC552640, LOC551806, LOC552653, LOC725527, LOC412056, LOC408539, LOC411997, LOC552144, LOC726724, LOC408456, LOC411790, LOC412430, LOC724952, LOC413739, LOC410915, LOC411935, LOC552592, LOC410578, LOC408517, LOC551263, LOC551553, LOC550918, LOC408828, LOC408478, LOC551250, LOC413259, LOC409467, nAChRb2, LOC411955, LOC102655740, LOC408634, LOC100578207, LOC408467, LOC551845, LOC414051, LOC727346, LOC408777, LOC726762, LOC100578810, LOC412764, LOC726412, LOC410967, nAChRa9, LOC725462, LOC725922, Vhdl	2.51×10^{-07}
	LOC725922, LOC725462, nAChRa9, LOC410967, LOC726412, LOC412764, LOC100578810, LOC726762, LOC408777, LOC727346, LOC414051, **Melt, LOC412925, LOC725165, LOC552610, LOC551541, LOC725346, Uqcr11, LOC551604, LCCH3, Hiscl1, ATP5G2, LOC408837, LOC410557, LOC412777, LOC412220, LOC726316, LOC726120, LOC102654955,**	

transmembrane transporter activity	**LOC727483, LOC411411, LOC409236, Eaat-2, LOC413517, LOC552682, LOC726747, LOC551766, Gat-1B, LOC551861, LOC724827, LOC412007, LOC409646, LOC552699, LOC406124, LOC408909, LOC411732, LOC724264, GluCl, LOC410371, LOC725881, LOC551680, Amel_8916, LOC551660, LOC552792, LOC412396, LOC725315, LOC102653800, Ant, LOC409114, LOC551782, Gat-a, Nmdar1, LOC409073, LOC410788, LOC408769, LOC100578557, LOC551454, nAChRa7, LOC409919, LOC551325, LOC410791, LOC413816, LOC410803, LOC409924, LOC551167, LOC410805, nAChRb1, LOC409636, nAChRa8, LOC408525, LOC409715, LOC410278, LOC100578218, LOC410612, pHCl, LOC409056, LOC411764, Para, LOC409192, LOC550915, LOC409565, nAChRa2, LOC552149, LOC551961, LOC725219, LOC413959, LOC409932, LOC551936, LOC410566, LOC412431, LOC551337, LOC551093, LOC409055, LOC551883, LOC411036, LOC552727, LOC408402, LOC410626, LOC411052, LOC100578899, LOC552476, LOC409897, LOC413020, LOC410997, LOC552720, LOC412830, LOC726423, LOC409899, LOC552704, LOC409666, LOC409074, LOC552744, LOC413934, nAChRa6, LOC552001, LOC552410, LOC726721, LOC413689, LOC552708,** LOC410062, LOC551894, LOC411403, LOC726513, LOC552552, LOC411807, LOC412741, LOC409424, LOC552640, LOC551806, LOC552653, LOC725527, LOC412056, LOC408539, LOC411997, LOC552144, LOC726724, LOC408456, LOC411790, LOC412430, LOC724952, LOC413739, LOC410915, LOC411935, LOC552592, LOC410578, LOC408517, LOC551263, LOC551553, LOC550918, LOC408828, LOC408478, LOC413259, LOC409467, nAChRb2, LOC102655740, LOC408634, LOC100578207, LOC408467, LOC551845, LOC414051, LOC727346, LOC408777, LOC726762, LOC100578810, LOC412764, LOC726412, LOC410967, nAChRa9, LOC725462, LOC725922	$1.27x10^{-06}$
structural constituent of cuticle	**CPR14, CPR19, LOC724624, CPR6, CPR5, LOC727578, CPR27, CPR3, LOC726725, LOC107964828, CPR11, CPR17,** CPR2, CPR4, LOC102653832, CPR16, CPR13, LOC102654371, CPR1, CPR21	$3.49x10^{-04}$
monoatomic ion transmembrane transporter activity	**LOC552610, LOC551541, Uqcr11, LCCH3, Hiscl1, ATP5G2, LOC408837, LOC410557, LOC412777, LOC726316, LOC726120, LOC102654955, LOC727483, LOC409236, LOC552682, LOC726747, LOC551766, LOC551861, LOC552699, LOC406124, LOC411732, GluCl, LOC410371, LOC551680, Amel_8916, LOC552792, LOC412396, LOC102653800, LOC409114, LOC551782, Nmdar1, LOC410788, LOC551454, nAChRa7, LOC551325, LOC410803, LOC410805, nAChRb1, nAChRa8, LOC408525, LOC409715, pHCl, LOC411764, Para, LOC550915, nAChRa2, LOC551961,**	$3.28x10^{-03}$

	**LOC725219, LOC551093, LOC409055, LOC551883, LOC411036, LOC100578899, LOC552476, LOC413020, LOC552720, LOC412830, LOC726423, LOC552704, LOC409074, nAChRa6, LOC552410, LOC413689,** LOC551894, LOC411403, LOC552552, LOC411807, LOC725527, LOC408539, LOC726724, nAChRb2, LOC100578207, LOC408467, LOC551845, LOC727346, LOC408777, nAChRa9	
passive transmembrane transporter activity	**Melt, LOC725165, LOC551604, LCCH3, Hiscl1, LOC727483, LOC409236, LOC552682, LOC551766, LOC552699, LOC406124, LOC411732, GluCl, LOC551680, Amel_8916, LOC552792, LOC102653800, LOC409114, LOC551782, Nmdar1, LOC410788, LOC551454, nAChRa7, LOC551325, LOC410805, nAChRb1, nAChRa8, LOC408525, pHCl, LOC411764, Para, nAChRa2, LOC552149, LOC725219, LOC551883, LOC411036, LOC100578899, LOC413020, LOC412830, LOC726423, nAChRa6,** LOC551894, LOC552552, LOC552640, LOC551806, LOC726724, LOC410915, LOC413259, nAChRb2, LOC100578207, LOC408467, LOC551845, LOC727346, LOC408777, nAChRa9	$3.28x10^{-03}$
proton transmembrane transporter activity	**LOC552610, LOC551541, Uqcr11, ATP5G2, LOC408837, LOC410557, LOC726316, LOC726120, LOC102654955, LOC727483, LOC409236, LOC552682, LOC726747, LOC551766, LOC551861, LOC552699, LOC412396, LOC409114, LOC551961, LOC551093, LOC409055, LOC552476, LOC552720, LOC409074, LOC552410,** LOC725527	$3.28x10^{-03}$
active transmembrane transporter activity	**LOC552610, LOC551541, LOC725346, Uqcr11, LOC408837, LOC412777, LOC726316, LOC411411, Eaat-2, LOC413517, LOC726747, Gat-1B, LOC724827, LOC408909, LOC724264, LOC410371, LOC725881, LOC551660, LOC412396, LOC725315, Ant, Gat-a, LOC409073, LOC408769, LOC409919, LOC413816, LOC551167, LOC409715, LOC100578218, LOC409056, LOC551961, LOC413959, LOC551337, LOC551093, LOC409055, LOC552476, LOC409897, LOC552720, LOC409666, LOC409074, LOC552744, LOC413934, LOC552410, LOC726721, LOC413689,** LOC726513, LOC412741, LOC725527, LOC411997, LOC724952, LOC410578, LOC550918, LOC414051, LOC410967	$3.28x10^{-03}$
	**Melt, LOC725165, LOC551604, LCCH3, Hiscl1, LOC727483, LOC409236, LOC552682, LOC551766, LOC552699, LOC406124, LOC411732, GluCl, LOC551680, Amel_8916, LOC552792, LOC102653800, LOC409114, LOC551782, Nmdar1, LOC410788, LOC551454, nAChRa7,**	$3.28x10^{-03}$

channel activity	**LOC551325, LOC410805, nAChRb1, nAChRa8, LOC408525, pHCl, LOC411764, Para, nAChRa2, LOC552149, LOC725219, LOC551883, LOC411036, LOC100578899, LOC413020, LOC412830, LOC726423, nAChRa6,** LOC551894, LOC552552, LOC552640, LOC551806, LOC726724, LOC410915, LOC413259, nAChRb2, LOC100578207, LOC408467, LOC551845, LOC727346, LOC408777, nAChRa9	
inorganic molecular entity transmembrane transporter activity	**LOC552610, LOC551541, Uqcr11, LOC551604, LCCH3, Hiscl1, ATP5G2, LOC408837, LOC410557, LOC412777, LOC726316, LOC726120, LOC102654955, LOC727483, LOC409236, LOC413517, LOC552682, LOC726747, LOC551766, LOC551861, LOC552699, LOC406124, LOC411732, GluCl, LOC410371, LOC551680, LOC412396, LOC409114, LOC551782, LOC551454, LOC413816, LOC410805, LOC409715, pHCl, LOC409056, LOC411764, Para, LOC551961, LOC725219, LOC551093, LOC409055, LOC551883, LOC411036, LOC100578899, LOC552476, LOC413020, LOC552720, LOC412830, LOC552704, LOC409074, LOC413934, LOC552410, LOC413689**, LOC411403, LOC411807, LOC552640, LOC725527, LOC724952, LOC100578207, LOC408467, LOC551845, LOC408777	$3.76x10^{-03}$
GTPase activity	**LOC408971, Ef-1a-f1, LOC410943, LOC725453, LOC412886, LOC552419, LOC100577548, LOC410996, LOC413034, LOC411351, LOC413438, LOC724762, LOC408328, LOC100578704, LOC550886, LOC408522, LOC725337, LOC411252, LOC107965449, LOC552730, LOC408556, LOC411085,** LOC409481, LOC409126, LOC410614, LOC724366, LOC726027, LOC411134, LOC409863, LOC102654987, LOC100577580, LOC552625, LOC413614, LOC552766, LOC409342, LOC102656587, LOC413827, LOC408388, EF1a-F2, LOC412030, LOC724594, LOC410280, LOC100577870, LOC552042, LOC411642, LOC550827, LOC410906, LOC552138, LOC411226, LOC726887, LOC411704, LOC409633, LOC410994	$1.01x10^{-02}$

The table presents the Gene ontology source (MF), term name, associated DEGs, and p-values. DEGs shown in bold are upregulated, while those in normal text are downregulated in solvent-treated adult group (SAG) compared to solvent-treated larvae group (SLG). The DEGs in each GO term are displayed from highest upregulation to highest downregulation.

Table S8: List of DEGs for GO_BP category in LAG compared to LLG

Term name	DEGs	p-value
mitochondrial ATP synthesis coupled electron transport	**LOC100578782, LOC552610, LOC551541, Uqcr11, LOC408837, LOC726316, LOC413340, LOC726747, LOC409473, LOC551757, LOC409586, LOC725881, LOC412396, LOC725712**	5.17x10^{-03}
aerobic electron transport chain	**LOC100578782, LOC552610, LOC551541, Uqcr11, LOC408837, LOC726316, LOC413340, LOC726747, LOC409473, LOC551757, LOC409586, LOC725881, LOC412396, LOC725712**	5.83x10^{-03}
small molecule metabolic process	**TyHyd, LOC726463, LOC408868, LOC408432, LOC551527, LOC410557, LOC726120, sGC-alpha1, LOC102654955, ATP5G2, LOC410059, LOC409236, LOC551861, LOC727483, LOC412541, LOC552682, Gycbeta1, LOC551766, LOC551631, LOC552699, LOC408950, LOC551103, LOC409114, LOC411576, LOC102655754, LOC409299, LOC410828, LOC410122, LOC552316, LOC409063, Ac3, Coq7, LOC724791, LOC410120, Tpi, LOC410639, LOC550885, LOC410612, LOC410638, LOC409963, LOC725204, LOC408818, LOC408441, LOC551154, LOC408474, LOC412082, LOC552644, LOC411189, LOC726754, LOC411916, LOC411771, LOC727293, LOC100577378, LOC725400, LOC410539, LOC551676, LOC551523, LOC410948, LOC726262, LOC411633, LOC725325, LOC724571, LOC551093, LOC550686, LOC552086, LOC726205, LOC409487, LOC102656439, LOC412069, LOC411014,** sGCbeta-3, LOC410234, LOC550673, GlnS, LOC724552, LOC551785, LOC551447, LOC410207, LOC413702, LOC552205, LOC725018, LOC409773, LOC725817, LOC552148, LOC412548, LOC552692, LOC551835, LOC413924, LOC409846, LOC551593, LOC409444, LOC550767, LOC408509, LOC412460, LOC412815, LOC410325, LOC725031, LOC409179, LOC411088, LOC551448, LOC551143, LOC408859, LOC412619, LOC413789, LOC551282, LOC412876, LOC724909, LOC409023, LOC410422, LOC551986, LOC550828, LOC408650, LOC411796, LOC409066, LOC412467	5.83x10^{-03}
aerobic respiration	**LOC100578782, LOC552610, LOC551541, Uqcr11, LOC409292, LOC410059, LOC408837, LOC726316, LOC552128, LOC413340, LOC726747, LOC409473, LOC551757, LOC409586, LOC725881, LOC724264, LOC408950, LOC410396, LOC412396, LOC409549,**	5.98x10^{-03}

	**LOC408446, LOC725566, LOC725712, LOC408734, LOC550686, LOC411014**	
generation of precursor metabolites and energy	**CytC, LOC100578782, LOC552610, LOC551541, Uqcr11, LOC409292, LOC410059, LOC408837, LOC726316, LOC552128, LOC413340, LOC726747, LOC409473, LOC551757, LOC409586, LOC727599, LOC725881, LOC724264, LOC408950, LOC410396, LOC725797, LOC412396, LOC409549, LOC411576, LOC102655754, LOC408446, LOC725566, LOC410122, LOC725712, Tpi, LOC408818, LOC551154, LOC552105, LOC107964213, LOC408734, LOC725325, LOC550686, LOC411014,** LOC552343, LOC551785, LOC409773, LOC550767	$5.98x10^{-03}$
proton motive force-driven ATP synthesis	**LOC410557, LOC726120, LOC102654955, ATP5G2, LOC409236, LOC551861, LOC727483, LOC552682, LOC551766, LOC552699, LOC409114**	$5.98x10^{-03}$
ATP biosynthetic process	**LOC410557, LOC726120, LOC102654955, ATP5G2, LOC409236, LOC551861, LOC727483, LOC552682, LOC551766, LOC552699, LOC409114**	$5.98x10^{-03}$
ATP metabolic process	**LOC410557, LOC726120, LOC102654955, ATP5G2, LOC409236, LOC551861, LOC727483, LOC552682, LOC551766, LOC552699, LOC409114, LOC411576, LOC102655754, LOC410122, Tpi, LOC408818, LOC551154, LOC551523, LOC551093**	$6.78x10^{-03}$
purine-containing compound metabolic process	**LOC410557, LOC726120, sGC-alpha1, LOC102654955, ATP5G2, LOC409236, LOC551861, LOC727483, LOC552682, Gycbeta1, LOC551766, LOC551631, LOC552699, LOC551103, LOC409114, LOC411576, LOC102655754, LOC409299, LOC410122, LOC552316, Ac3, Tpi, LOC550885, LOC408818, LOC408441, LOC551154, LOC727293, LOC551523, LOC726262, LOC725325, LOC551093, LOC550686, LOC409487, LOC102656439,** sGCbeta-3, LOC550673, LOC551785, LOC725817, LOC409846, LOC409444, LOC550767, LOC412619, LOC412467	$8.50x10^{-03}$
nucleotide metabolic process	**LOC410557, LOC726120, sGC-alpha1, LOC102654955, ATP5G2, LOC409236, LOC551861, LOC727483, LOC552682, Gycbeta1, LOC551766, LOC551631, LOC552699, LOC551103, LOC409114,**	$1.10x10^{-02}$

		**LOC411576, LOC102655754, LOC409299, LOC410122, LOC552316, Ac3, Tpi, LOC550885, LOC408818, LOC551154, LOC408474, LOC410539, LOC551523, LOC726262, LOC725325, LOC724571, LOC551093, LOC550686, LOC726205, LOC409487,** sGCbeta-3, LOC550673, LOC551785, LOC725817, LOC552148, LOC551835, LOC409846, LOC409444, LOC550767, LOC551448, LOC412619, LOC551986, LOC412467	

The table presents the Gene ontology source (BP), term name, associated DEGs, and p-values. DEGs shown in bold are upregulated, while those in normal text are downregulated in LCY-treated adult group (LAG) compared to LCY-treated larvae group (LLG). The DEGs in each GO term are displayed from highest upregulation to highest downregulation.

Table S9: List of DEGs for GO_BP category in SAG compared to SLG

Term name	Differentially Expressed Genes	P-Value
mitochondrial ATP synthesis coupled electron transport	**LOC552610, LOC551541, Uqcr11, LOC408837, LOC726316, LOC413340, LOC100578782, LOC409473, LOC726747, LOC551757, LOC409586, LOC725881, LOC412396, LOC725712**	$2.40x10^{-03}$
aerobic respiration	**LOC552610, LOC551541, Uqcr11, LOC408837, LOC410059, LOC409292, LOC726316, LOC552128, LOC413340, LOC100578782, LOC409473, LOC726747, LOC408950, LOC551757, LOC409586, LOC724264, LOC725881, LOC412396, LOC725566, LOC409549, LOC408446, LOC410396, LOC725712, LOC411014, LOC550686, LOC408734, Pcl**	$2.40x10^{-03}$
generation of precursor metabolites and energy	**CytC, LOC552610, LOC551541, Uqcr11, LOC408837, LOC410059, LOC409292, LOC726316, LOC552128, LOC413340, LOC100578782, LOC409473, LOC726747, LOC408950, LOC551757, LOC409586, LOC725797, LOC724264, LOC727599, LOC725881, LOC412396, LOC725566, LOC409549, LOC408446, LOC410396, LOC411576, Tpi, LOC410122, LOC725712, LOC102655754, LOC408818, LOC551154, LOC725325, LOC411014, LOC107964213, LOC552105, LOC550686, LOC552712, LOC408734, Pcl,** LOC409773, LOC550767	$3.09x10^{-03}$
monoatomic ion transmembrane transport	**LOC411664, LCCH3, Hiscl1, ATP5G2, LOC726268, LOC551766, LOC406124, LOC411732, GluCl, LOC551680, Amel_8916, LOC409114, LOC102656594, Nmdar1, LOC551454, nAChRa7, LOC551325, LOC410805, nAChRb1, nAChRa8, LOC408525, pHCl, Para, nAChRa2, LOC551961, LOC725219, LOC551093, LOC409055, LOC551883, LOC411036, LOC100578899, LOC552476, LOC413020, LOC552720, LOC412830, LOC726423, LOC409074, nAChRa6, LOC552410,** LOC411403, LOC102654905, LOC725645, nAChRb2, Tsf1, LOC551845, LOC408777, nAChRa9	$6.71x10^{-03}$
ATP metabolic process	**ATP5G2, LOC410557, LOC726120, LOC102654955, LOC727483, LOC409236, LOC552682, LOC551766, LOC551861, LOC552699, LOC409114, LOC411576, Tpi, LOC410122, LOC102655754, LOC408818, LOC551154, LOC551093, LOC551523**	$4.17x10^{-03}$
aerobic electron transport chain	**LOC552610, LOC551541, Uqcr11, LOC408837, LOC726316, LOC413340, LOC100578782, LOC409473, LOC726747, LOC551757, LOC409586, LOC725881, LOC412396, LOC725712**	$3.09x10^{-03}$
proton motive force- driven ATP synthesis	**ATP5G2, LOC410557, LOC726120, LOC102654955, LOC727483, LOC409236, LOC552682, LOC551766, LOC551861, LOC552699, LOC409114**	$4.17x10^{-03}$

ATP biosynthetic process	**ATP5G2, LOC410557, LOC726120, LOC102654955, LOC727483, LOC409236, LOC552682, LOC551766, LOC551861, LOC552699, LOC409114**	$4.17x10^{-03}$
cellular respiration	**LOC552610, LOC551541, Uqcr11, LOC408837, LOC410059, LOC409292, LOC726316, LOC552128, LOC413340, LOC100578782, LOC409473, LOC726747, LOC408950, LOC551757, LOC409586, LOC724264, LOC725881, LOC412396, LOC725566, LOC409549, LOC408446, LOC410396, LOC725712, LOC411014, LOC550686, LOC408734, Pcl**	$4.51x10^{-03}$
ATP synthesis coupled electron transport	**LOC552610, LOC551541, Uqcr11, LOC408837, LOC726316, LOC413340, LOC100578782, LOC409473, LOC726747, LOC551757, LOC409586, LOC724264, LOC725881, LOC412396, LOC725712**	$6.71x10^{-03}$

The table presents the Gene ontology source (BP), term name, associated DEGs, and p-values. DEGs shown in bold are upregulated, while those in normal text are downregulated in solvent-treated adult group (SAG) compared to solvent-treated larvae group (SLG). The DEGs in each GO term are displayed from highest upregulation to highest downregulation.

Table S10: List of significant DEGs for each enriched KEGG pathway in LLG compared to SLG.

KEGG pathway	DEGs	p-value	Bonferroni	FDR
Neuroactive ligand-receptor interaction	**LOC113219265, LOC409626, LOC409143, LOC410894, nAChRa9, SIFR, LOC411672, LOC410140, LOC726953, LOC413829, LOC411760, LOC726393, LOC724473,** Dop1, LOC726755	5.38×10^{-11}	5.92×10^{-09}	5.92×10^{-09}
Motor proteins	**LOC410996, LOC100577548, LOC411814, TpnT, LOC408583, LOC552070, LOC410851, LOC725593, LOC408414, LOC409869,** LOC725469, LOC100578990, LOC100578575, LOC113218800, LOC410994, LOC551109, LOC413205	2.89×10^{-09}	3.17×10^{-07}	1.59×10^{-07}
Ascorbate and aldarate metabolism	**LOC408559, LOC411140, LOC550687, LOC411202, LOC411021, LOC725997, LOC408788, LOC408650,** LOC413043	6.93×10^{-09}	7.62×10^{-07}	2.54×10^{-07}
Tyrosine metabolism	**LOC410638, LOC725204, LOC551465, LOC727622, LOC552210, LOC412675, LOC725400,** LOC410639	9.35×10^{-09}	1.03×10^{-06}	2.57×10^{-07}
Tryptophan metabolism	**LOC408559, LOC410638, LOC411200, LOC411140, LOC412569, LOC550687, LOC409150, LOC724811,** LOC410639	1.69×10^{-08}	1.86×10^{-06}	3.72×10^{-07}
Pentose and glucuronate interconversions	**LOC411100, LOC411202, LOC411021, LOC725997, LOC412231, Cryl1, LOC551968, LOC408788,** LOC413043	5.39×10^{-08}	5.93×10^{-06}	9.88×10^{-07}
Glycolysis / Gluconeogenesis	**LOC408559, LOC413924, LOC411140, LOC550687, LOC411202, LOC550785, LOC408818, LOC551103, Tpi,** LOC100577002, LOC724619	1.05×10^{-07}	1.15×10^{-05}	1.64×10^{-06}
Phenylalanine metabolism	**LOC410638, LOC725204, LOC727622, LOC412675, LOC725400,** LOC410639	1.25×10^{-07}	1.38×10^{-05}	1.72×10^{-06}
Valine, leucine and isoleucine degradation	**LOC408559, LOC411140, LOC550687, LOC409150, LOC408291, LOC409157, LOC408288, LOC409712, LOC408955**	5.90×10^{-07}	6.49×10^{-05}	7.21×10^{-06}
Fatty acid degradation	**LOC408559, LOC411140, LOC550687, LOC409150, LOC408291, LOC551837, LOC409712, LOC409515**	7.75×10^{-07}	8.52×10^{-05}	8.52×10^{-06}

The table presents the KEGG pathways, associated DEGs, p-values, Bonferroni corrections, and false discovery rates (FDR). DEGs shown in bold are upregulated, while those in normal text are downregulated in LCY-treated larvae group (LLG) compared to solvent-treated larvae group (SLG). The DEGs in each KEGG pathway are displayed from highest upregulation to highest downregulation.

Table S11: List of significant DEGs for each enriched KEGG pathway in LAG compared to SAG.

KEGG pathway	DEGs	p-value	Bonferroni	FDR
Biosynthesis of unsaturated fatty acids	**LOC727166,** LOC724226, LOC100578829	$3.41x10^{-03}$	$1.02x10^{-01}$	$1.02x10^{-01}$
Neuroactive ligand-receptor interaction	**LOC411672, nAChRa9, Akhr**	$1.40x10^{-02}$	$4.21x10^{-01}$	$2.11x10^{-01}$
Tyrosine metabolism	**LOC551465,** LOC410948	$4.15x10^{-02}$	1.00	$3.01x10^{-01}$
Motor proteins	LOC100578129, LOC551109, LOC102655879	$4.19x10^{-02}$	1.00	$3.01x10^{-01}$

The table presents the KEGG pathways, associated DEGs, p-values, Bonferroni corrections, and false discovery rates (FDR). DEGs shown in bold are upregulated, while those in normal text are downregulated in LCY-treated adult group (LAG) compared to solvent-treated adult group (SAG). The DEGs in each KEGG pathway are displayed from highest upregulation to highest downregulation.

Table S12: List of significant DEGs for each enriched KEGG pathway in SAG compared to SLG.

KEGG Pathway	Differentially Expressed Genes	PValue	Bonferroni	FDR
**Oxidative phosphorylation**	**CytC, LOC552610, Uqcr11, LOC413605, ATP5G2, LOC408837, LOC410557, LOC726316, LOC726120, LOC102654955, LOC411183, LOC727483, LOC411411, LOC726042, LOC727049, LOC550667, LOC409236, LOC413340, LOC100578782, LOC551169, LOC408477, LOC409473, LOC409930, LOC412328, LOC552682, Ndufs5, LOC413891, LOC726747, LOC551766, Cox6c, LOC551861, LOC724827, LOC551866, LOC551757, LOC551078, LOC552699, LOC408909, LOC409793, LOC409586, LOC725797, LOC727199, LOC724264, LOC552671, LOC413014, LOC727599, LOC408367, LOC551042, LOC725881, LOC552424, LOC552809, LOC551660, LOC409103, Cox6b1, LOC726297, LOC412396, LOC724719, Ndufb2, LOC725315, LOC725566, LOC409549, LOC409114, LOC100302106, LOC102654169, LOC102653862, Ndufs1, LOC727026, LOC725712, LOC412810, LOC100578006, LOC113219091, LOC411892, LOC551961, LOC551093, LOC409055, LOC552476, LOC409420, LOC552720, LOC409074, LOC408734, LOC552410,** LOC410617, LOC725527, LOC551917, LOC100576847	5.59 X 10^{-61}	7.54 X 10^{-59}	7.54 X 10^{-59}
**Motor proteins**	**LOC411316, LOC409843, TpnT, LOC410489, TpnI, LOC408583, LOC408414, LOC725479, LOC411814, LOC409937, LOC550973, LOC412886, LOC100577548, LOC413837, LOC410996, LOC411197, LOC724291, LOC102656311, LOC410851, LOC726044, LOC100578704, LOC726015, LOC552299, LOC726176, LOC409530, LOC408444, LOC412262, LOC551652, LOC413097, LOC411894**, LOC410614, LOC100576290, LOC726456, LOC551438, LOC552766, LOC410960, LOC408388, LOC552070, Arp1, LOC726683, LOC724859, LOC100578990, LOC100577649, LOC100578129, LOC550827, LOC412638, LOC411226, LOC409869, LOC725239, LOC412092, LOC552512, LOC725593, LOC551109, LOC413205, LOC726506, LOC113218800, LOC551711, LOC411018, LOC552508, LOC724193, LOC725469, LOC410994	4.30 X 10^{-26}	5.81 X 10^{-24}	2.90 X 10^{-24}
**Neuroactive ligand receptor interaction**	**LOC113219265, LOC552301, LOC410894, LOC410979, LOC726935, LCCH3, LOC409626, LOC408534, LOC406124, LOC411220, Amel_8916, LOC409143, LOC113218647, Nmdar1, DopR2, 5-HT2beta, LOC726953, LOC411420, SIFR, 5-HT1, Crzr, LOC413997, 5-HT2alpha, LOC412818, LOC411611, Dop1, LOC100578877, LOC412299, LOC410140, Dop3, LOC412830,** LOC552552, LOC724495,	6.09 X 10^{-23}	8.22 X 10^{-21}	2.74 X 10^{-21}

	mGlutR1, LOC413164, LOC413829, LOC411760, LOC113218562, Akhr, nAChRa9, LOC726755, LOC100576135, LOC100576637			
**Citrate cycle (TCA cycle)**	**LOC551039, PDHB, LOC410059, LOC409292, LOC552128, LOC550667, LOC551169, LOC408950, LOC408286, LOC551103, LOC724321, LOC551631, LOC725566, LOC409549, LOC408446, LOC409155, LOC551958, LOC410396, LOC100576646, LOC551403, LOC413768, LOC411014, LOC550686, LOC412522, LOC408734, Pcl,** LOC100578735, LOC409485, LOC412876, LOC551917, LOC412843	1.61×10^{-20}	2.17×10^{-18}	5.43×10^{-19}
**Glycolysis / Gluconeogenesis**	**LOC551039, PDHB, LOC551005, LOC100577002, LOC551103, LOC724724, LOC551631, LOC551927, LOC552736, LOC411576, LOC100576646, LOC552678, LOC552007, Tpi, LOC410122, LOC102655754, LOC409751, LOC408818, LOC551154, LOC550785, LOC724619, LOC409624, LOC552086, LOC412522, LOC411202, LOC725455**, LOC409773, LOC413336, LOC412362, LOC725351, LOC100578735, LOC727456, LOC408559, LOC411140, LOC411188, LOC412843	2.87×10^{-20}	3.88×10^{-18}	7.76×10^{-19}
**Purine metabolism**	**Adk1, sGC-alpha1, Gycbeta1, LOC408699, LOC552216, LOC413048, LOC409299, LOC410539, LOC552316, LOC102653763, Ac3, LOC408441, LOC724131, LOC726069, LOC724389, LOC411288, LOC410676, LOC726444, LOC551373, LOC726514, LOC552514, LOC413601, LOC551523, LOC552583, LOC727293, LOC102656439, LOC410630, LOC726205,** LOC412573, LOC409444, LOC550673, LOC410482, LOC414008, LOC552535, LOC724933, LOC409846, LOC412362, LOC551770, LOC724603, LOC725673, LOC552657, LOC552840, LOC412619, LOC10057767	9.91×10^{-20}	1.34×10^{-17}	2.23×10^{-18}
**ATP-dependent chromatin remodeling**	**LOC551521, LOC100578006, LOC552481, LOC100576784, LOC551914, LOC550814,** LOC410033, LOC412742, LOC100578927, LOC552582, LOC413341, LOC724678, LOC413318, LOC411629, LOC726619, LOC408891, LOC411279, LOC551881, LOC411727, LOC413181, LOC552200, LOC726816, LOC412077, LOC725795, LOC100576104, LOC725308, LOC409501, LOC552094, LOC410502, LOC724311, LOC551071, LOC413793, Arp1, LOC412046, LOC100576266, Brd7, LOC107963990, LOC552824, LOC551779, LOC411503, LOC552031, LOC725450	1.85×10^{-19}	2.50×10^{-17}	3.57×10^{-18}
**Wnt signaling pathway**	**LOC724471, Camkii, LOC552419, LOC107963993, LOC409791, LOC100579005, LOC408996, LOC725511, Pkc, LOC408278,** LOC408890, LOC409321, LOC413169, LOC726280, arm, LOC409050, LOC408791, LOC552545, LOC410129, LOC410808,	1.56×10^{-17}	2.11×10^{-15}	2.63×10^{-16}

	LOC413183, LOC726671, LOC413501, LOC413616, LOC410724, LOC724997, LOC551517, LOC551021, LOC409158, LOC724501, LOC410956, LOC551508, LOC552138, LOC725720, LOC100576247, LOC552805, LOC552340, LOC411919, LOC413502, LOC724378, LOC551124, LOC410190, LOC413500.			
**Pyruvate metabolism**	**LOC724904, LOC551039, PDHB, LOC408950, LOC551103, LOC724321, LOC551631, LOC551927, LOC100576646, LOC552639, LOC410042, LOC552007, LOC409682, LOC411014, LOC409624, LOC552286, LOC412522, LOC552568, LOC411202,** LOC409773, LOC411813, LOC100578735, LOC412876, LOC726218, LOC408559, LOC411140, LOC411188, LOC412843	5.25×10^{-15}	7.09×10^{-13}	7.88×10^{-14}
**mTOR signaling pathway**	**LOC100578725, LOC411892, LOC551961, LOC725511, LOC551093, LOC409055, Pkc, LOC552476, LOC409577, LOC552720, LOC727159, LOC409036, LOC412531, LOC725584, LOC409317, LOC727552,** LOC552718, InR-2, LOC724472, LOC725647, LOC410201, LOC409725, LOC413430, LOC409050, LOC552381, LOC551512, Pten, LOC550857, LOC413501, LOC408577, LOC551021, LOC409702, LOC411935, LOC725789, LOC412031, LOC551198, LOC411297, LOC100576247, LOC411919, LOC413502, LOC551124, LOC413500	1.30×10^{-14}	1.75×10^{-12}	1.75×10^{-13}

The table presents the KEGG pathways, associated DEGs, p-values, Bonferroni corrections, and false discovery rates (FDR). DEGs shown in bold are upregulated, while those in normal text are downregulated in solvent-treated adult group (SAG) compared to solvent-treated larvae group (SLG). The DEGs in each KEGG pathway are displayed from highest upregulation to highest downregulation.

Table S13: List of significant DEGs for each enriched KEGG pathway in LAG compared to LLG.

KEGG pathway	DEGs	p-value	Bonferroni	FDR
Oxidative phosphorylation	**CytC, LOC100578782, LOC552610, LOC413605, LOC727049, Uqcr11, LOC410557, LOC550667, LOC726120, LOC102654955, ATP5G2, LOC408837, LOC726316, LOC726042, LOC413340, LOC411183, LOC411411, LOC409236, LOC726222, Cox6c, LOC413891, LOC551861, LOC726747, LOC408477, LOC727483, LOC551169, LOC724827, LOC409473, LOC100302106, LOC552682, LOC551766, LOC412328, LOC409930, LOC551866, LOC551757, LOC727199, LOC408367, LOC409103, LOC409586, LOC413014, Ndufb2, LOC408909, LOC409793, LOC551078, LOC727599, Cox6b1, LOC552699, LOC725881, LOC724264, Ndufs5, LOC725315, LOC724719, LOC552424, LOC551660, LOC726297, LOC725797, LOC552671, LOC102654169, LOC551042, LOC552809, LOC412396, LOC409549, LOC409114, LOC725566, LOC727026, LOC102653862, Ndufs1, LOC725712, LOC113219091, LOC100578006, LOC412810, LOC551961, LOC408734, LOC411892, LOC409055, LOC552476, LOC551093, LOC409420, LOC409074,** LOC725527, LOC410617, LOC100576847, LOC551917	1.77×10^{-56}	2.37×10^{-54}	2.37×10^{-54}
Motor proteins	**LOC411316, LOC409843, LOC410489, LOC410959, TpnT, LOC725479, TpnI, LOC411639, LOC409937, LOC550973, LOC408414, LOC408583, LOC412886, LOC413837, LOC726044, LOC100578704, LOC724291, LOC726015, LOC411814, LOC102656311, LOC100577548, LOC552299, LOC413097, LOC408444, LOC410851, LOC409676, LOC100578575, LOC726176,** LOC552766, LOC411769, LOC411602, LOC726456, LOC725068, LOC100576290, LOC412267, LOC409581, LOC410960, LOC551438, LOC726683, LOC100577649, LOC725239, LOC408388, LOC411226, Arp1, LOC412638, LOC550827, LOC724859, LOC100578129, LOC552070, LOC113218800, LOC412092, LOC409869, LOC552512, LOC725593, LOC411018,	2.59×10^{-25}	3.47×10^{-23}	1.74×10^{-23}

	LOC726506, LOC551109, LOC551711, LOC552508, LOC724193, LOC102655879, LOC410994, LOC725469			
Glycolysis / Gluconeogenesis	**PDHB, LOC100577002, LOC551039, LOC551005, LOC551927, LOC551631, LOC724724, LOC551103, LOC552736, LOC100576646, LOC411576, LOC102655754, LOC552007, LOC552678, LOC410122, LOC724619, Tpi, LOC408818, LOC551154, LOC409751, LOC725455, LOC411189, LOC550785, LOC409624, LOC412522, LOC552086,** LOC411202, LOC409773, LOC413336, LOC413924, LOC100578735, LOC725351, LOC412362, LOC550687, LOC727456, LOC412843, LOC411188, LOC411140, LOC408559	$1.26x10^{-23}$	$1.69x10^{-21}$	$5.65x10^{-22}$
Purine metabolism	**Adk1, sGC-alpha1, Gycbeta1, LOC102653763, LOC408699, LOC552216, LOC409299, LOC552316, Ac3, LOC411288, LOC724131, LOC408441, LOC724389, LOC410676, LOC410630, LOC413048, LOC726444, LOC727293, LOC410539, LOC551523, LOC413274, LOC726514, LOC726262, LOC551373, LOC724571, LOC726205, LOC102656439, LOC552583, LOC552514,** sGCbeta-3, LOC550673, LOC724718, LOC552535, LOC410482, LOC551770, LOC409846, LOC409444, LOC725673, LOC724603, LOC552657, LOC552840, LOC412362, LOC551448, LOC724933, LOC412619, LOC100577675, LOC412467	$3.30x10^{-22}$	$4.43x10^{-20}$	$1.11x10^{-20}$
Peroxisome	**LOC412541, Sod2, LOC102654488, LOC552429, LOC100576735, LOC412986, LOC113218958, LOC100578814, LOC107965313, LOC410288, Sod1, LOC552256, LOC100577607, LOC724560, LOC411983, LOC725343, LOC410889,** LOC724262, LOC724718, LOC725187, LOC100578624, LOC725018, LOC724570, LOC552692, LOC727505, LOC102655950, LOC100576895, LOC412341, LOC552495, LOC413491, LOC551241, LOC408904, LOC409986, LOC724933, LOC552738, LOC552110, LOC727257, LOC552757, LOC552771, Cat, LOC100578329	$5.28x10^{-19}$	$7.08x10^{-17}$	$1.42x10^{-17}$
Wnt signaling pathway	**LOC724471, Camkii, LOC107963993, LOC552419, LOC725511, LOC100579005, Pkc, LOC409791, LOC408996,** LOC413501, LOC413169, LOC411917, LOC727294,	$7.75x10^{-19}$	$1.04x10^{-16}$	$1.73x10^{-17}$

	LOC409767, LOC725720, LOC410724, LOC724997, LOC552545, LOC409050, LOC409321, LOC726280, LOC552374, LOC410129, LOC409001, LOC413616, LOC724501, LOC413183, LOC726671, LOC410808, LOC551021, LOC410956, LOC551517, LOC409158, LOC100576247, LOC408791, LOC551508, LOC552340, LOC552138, LOC410190, LOC552805, LOC411919, LOC724378, LOC413502, LOC413500, LOC551124			
ATP-dependent chromatin remodeling	**LOC551521, LOC100578006, LOC551914, LOC552481, LOC100576784, LOC726923, LOC107963965, LOC550814,** LOC726619, LOC411279, LOC408891, LOC413341, LOC411629, LOC100578927, LOC412077, LOC411727, LOC551881, LOC100576104, LOC726816, LOC552200, LOC551071, LOC410502, LOC413181, LOC725795, LOC107963990, LOC724519, LOC552094, LOC100576266, LOC551779, LOC725308, LOC409501, LOC724311, Brd7, LOC552824, LOC411503, LOC412046, Arp1, LOC413793, LOC552031, LOC725450, LOC726468	$5.95x10^{-18}$	$7.98x10^{-16}$	$1.14x10^{-16}$
Nucleocytoplasmic transport	**Ef-1a-f1, LOC102656258, LOC413378, LOC412950, LOC725134,** LOC413940, LOC410864, LOC727447, LOC409169, LOC551725, LOC412835, LOC551707, LOC413365, LOC409866, LOC100578879, LOC412464, LOC408625, LOC726159, LOC411909, LOC550834, LOC725396, LOC409229, LOC412511, LOC409308, LOC413343, LOC411918, LOC410343, LOC100577501, LOC409543, LOC408842, LOC726354, LOC413173, LOC413675, LOC727561, EF1a-F2, LOC412584, LOC410280, LOC413636, LOC411865, LOC100576146, LOC725332, LOC413146, LOC412817, LOC413018, LOC413344, LOC552567, LOC410447	$1.93x10^{-17}$	$2.58x10^{-15}$	$3.23x10^{-16}$
Citrate cycle (TCA cycle)	**PDHB, LOC551039, LOC550667, LOC409292, LOC410059, LOC552128, LOC551169, LOC724321, LOC551631, LOC408950, LOC410396, LOC408286, LOC551103, LOC409155, LOC409549, LOC551958, LOC100576646, LOC408446, LOC725566, LOC551403, LOC408734, LOC412522, LOC550686, LOC411014,** LOC100578735, LOC409485, LOC412876, LOC551917, LOC412843	$2.36x10^{-17}$	$3.16x10^{-15}$	$3.51x10^{-16}$

mTOR signaling pathway	**LOC100578725, LOC725511, LOC412531, Pkc, LOC551961, LOC411892, LOC409577, LOC727552, LOC727159, LOC409036, LOC409055, LOC552476, LOC725584, LOC551093, LOC410345,** LOC413501, LOC409523, LOC724408, LOC413365, LOC409725, LOC409925, LOC552718, InR-2, LOC413430, LOC409050, LOC412031, LOC552374, LOC725855, LOC550857, Pten, LOC551021, LOC552381, LOC100576247, LOC410201, LOC551512, LOC409702, LOC408577, LOC551198, LOC725789, LOC411935, LOC411297, LOC411919, LOC413502, LOC413500, LOC551124	1.52×10^{-16}	2.03×10^{-14}	1.88×10^{-15}

The table presents the KEGG pathways, associated DEGs, p-values, Bonferroni corrections, and false discovery rates (FDR). DEGs shown in bold are upregulated, while those in normal text are downregulated in LCY-treated adult group (LAG) compared to LCY-treated larvae group (LLG). The DEGs in each KEGG pathway are displayed from highest upregulation to highest downregulation.

Table S14. Summary of qRT-PCR validation results for selected differentially expressed genes across treatment comparisons.

Comparison	Gene Name	Mean FC	S.D.	S.E.M.	p-value
SAG vs SLG	*TpnCIIIb*	29531	20937	6979	< 0.0001
SAG vs SLG	*Melt*	17718	11391	3797	< 0.0001
SAG vs SLG	*LOC725364*	55761	37783	12594	< 0.0001
SAG vs SLG	*Hex110*	-560.9	393.1	131	< 0.0001
SAG vs SLG	*Fibroin4*	-4957	4905	1635	< 0.0001
SAG vs SLG	*Vhdl*	-1506	1498	499.2	< 0.0001
LAG vs LLG	*CutP*	33.94	0.8	0.27	< 0.0001
LAG vs LLG	*D-GalT*	60.42	3.15	1.05	< 0.0001
LAG vs LLG	*AGLU2*	81.75	9.35	3.12	< 0.0001
LAG vs LLG	*Hex70c*	-15149	3428	1143	< 0.0001
LAG vs LLG	*LIG1-X2*	-1273	641	213.7	< 0.0001
LAG vs LLG	*OBP13*	-2527	111.2	37.07	< 0.0001
LLG vs SLG	*POX*	2.73	0.61	0.2	< 0.0001
LLG vs SLG	*ZnCPT*	1.18	0.29	0.1	ns
LLG vs SLG	*CutP14*	1.27	0.99	0.33	ns
LLG vs SLG	*TERT*	-1.65	0.25	0.08	< 0.05
LLG vs SLG	*ORC5*	-1.71	0.31	0.1	< 0.05
LLG vs SLG	*Y-G*	-2.62	1.56	0.52	ns
LAG vs SAG	*GDH-FQ-X2*	1.07	0.65	0.22	ns
LAG vs SAG	*cili-TTC17*	1.61	0.43	0.14	< 0.05
LAG vs SAG	*CYB561D2*	1.54	0.42	0.14	< 0.05
LAG vs SAG	*ELOVL1*	-3.79	0.99	0.33	< 0.0001
LAG vs SAG	*KIF9*	-53.41	20.07	6.69	< 0.0001
LAG vs SAG	*DNAJC18L*	-3	0.78	0.26	< 0.005

Statistical analysis was conducted using the Mann–Whitney U test. Mean fold change (Mean FC), standard deviation (S.D.), standard error of the mean (S.E.M.), and corresponding p-values are shown.
